# Supplementary material for: Successfully implementing and embedding guidelines to improve the nutrition and growth of preterm infants in neonatal intensive care: a prospective interventional study
Source: BMJ Open. 2017 Dec 6;7(12):e017727. doi: 10.1136/bmjopen-2017-017727 (PMC5728292; doi:10.1136/bmjopen-2017-017727)
Supplement: Supplementary file 1 [file bmjopen-2017-017727supp001.pdf]

# Guidelines for the Nutritional Care of Infants in the Neonatal Unit

**Version: 1.0**

**Issued: December 2011**

**Review date: December 2014**

**Author:** **Dr Alison Leaf**

The procedural aspects of this guideline can be found in the document entitled:-

Guideline Proforma **- Guidelines for the Nutritional Care of Infants in the Neonatal Unit**

University Hospital Southampton NHS
NHS Foundation Trust

## Contents


| Paragraph | | Page |
|---|---|---|
| | **Executive Summary** | 3 |
| 1. | Introduction | 4 |
| 2. | Definitions | 5 |
| 3. | Roles and Responsibilities | 6 |
| 4. | Related Trust Documents | 7 |
| 5. | Guideline | |
| | **1. Aims and objectives; audit points** | 8 |
| | **2. Assessment and monitoring** | |
| | i. Initial assessment: growth / risk category / screen | 9 |
| | ii. On-going assessment: growth / biochemistry / nutrition screening / nutrition team | 10 |
| | **3. Nutrient requirements** | 11 |
| | **4. Standard nutritional support of preterm and sick infants** | 12 |
| | a. Overview by risk group | 12 |
| | b. Parenteral nutrition | 13 |
| | i. Indications for PN | 13 |
| | ii. Starting PN | 13 |
| | iii. Stock PN solutions | 13 |
| | iv. Pharmacy-manufactured ('bespoke') PN | 13 |
| | v. Reducing PN as feeds increase | 14 |
| | vi. Peripheral PN | 14 |
| | vii. Cautions | 14 |
| | c. Enteral nutrition | 15 |
| | i. Starting feeds | 15 |
| | ii. Choice of milk | 15 |
| | iii. Advancing feeds | 15 |
| | iv. Routine nutritional supplements | 16 |
| | v. Nutrition at discharge | 16 |
| | **5. Management of common gut and feeding problems** | 17 |
| | a. Feed intolerance / gastric residuals | 17 |
| | b. Abdominal distension | 17 |
| | c. Suspected Necrotising Enterocolitis (NEC) | 17 |
| | d. Suspected Gastro-oesophageal reflux (GOR) | 17 |
| | e. Suspected Food Protein Intolerance | 17 |
| | **6. Management of babies with surgical bowel problems** | 18 |
| | **7. Flow Charts** | 20 |
| | a. Starting and Increasing Feeds – High Risk Infants | 20 |
| | b. Starting and Increasing Feeds – Moderate Risk Infants | 21 |
| | c. Management of common feed related problems | 22 |
| | d. Choice of milk | 23 |
| | **8. Tables** | 24 |
| | a. Starting and increasing feeds | 24 |
| | b. Nutrient content of commonly used products | 25 |
| | **9. Supporting Information** | 26 |
| 6. | References | 32 |
| **Appendices** | | |
| Appendix 1 | Nutrient Requirements of preterm infants | 35 |
| Appendix 2 | Neonatal Nutrition Screening Tool | 36 |

University Hospital Southampton NHS
NHS Foundation Trust

# Executive Summary

Good nutrition is important at all stages of life. Babies are born at a time of rapid growth and formation of body tissues and organs, yet immature metabolism means they are unable to cope with either excess or lack of nutrients. Detail in both the quantity and quality of nutrients is critically important.

There is good evidence that mother's breast milk confers many advantages to baby, mother and to the formation of the parental bond. As well as containing just the right nutrients for human development, breast milk contains many factors which promote immune function and enable healthy intestinal development. Breast milk and breast-feeding should be encouraged in almost all situations.

Preterm infants and those with congenital abnormalities or metabolic disorders may require nutrient supplements or special feeds, and may require a period of intravenous nutrition until the gut is able to support their needs.

Measuring growth and monitoring biochemical well-being is crucial to optimising nutrition in high risk individuals.

These guidelines aim to provide both practical and theoretical guidance for the optimal nutrition of sick and preterm infants in the NNU at Southampton.

# 1. Introduction

- Good early growth is essential for long term health and well-being of all babies.
- Achieving recommended nutrient intake in very low birth-weight and sick infants is difficult particularly in the first weeks of life and development of a significant nutrient deficit is common. It is then very difficult to 'catch up'.
- Protein intake is particularly difficult to achieve.
- These guidelines aim to support decision-making such that nutrient delivery can be optimised. Close monitoring of intakes, biochemical status and growth is essential to monitor how well this is achieved.
- ***Every feed and every day is important – being aware of daily intake of key nutrients is the first step to improving growth and development***
- SENNAT (Southampton Electronic Neonatal Nutrition Assessment Tool) has been developed to help us all measure and monitor nutrient intakes and growth

**These guidelines are based on recommendations of:**

- Enteral nutrient supply for preterm infants: commentary from the European Society of Paediatric Gastroenterology, Journal of Pediatric Gastroenterology and Nutrition 2010[1]

- Nutrition of the Preterm Infant: Scientific basis and Practical Guidelines (second edition). Tsang RC, Uauy R, Koletzko B, Zlotkin S. Digital Educational Publishing 2005[2]

- Guidelines on Paediatric Parenteral Nutrition of the European Society of Paediatric Gastroenterology, Hepatology and Nutrition (ESPGHAN) and the European Society for Clinical Nutrition and Metabolism (ESPEN), Supported by the European Society of Paediatric Research (ESPR), Journal of Pediatric Gastroenterology and Nutrition 2005[3]

- Vermont Oxford Network 'Potentially Better Practices (PBPs) for Nutrition' as laid out in Pediatrics, 2003[4]

- Management and support of infant feeding in maternity facilities. Infant and young child feeding : model chapter for textbooks for medical students and allied health professionals., World Health Organisation 2009[5]

- Optimal feeding of low-birth-weight infants, World Health Organisation, 2006[6]

- UNICEF Baby Friendly Initiative, http://www.unicef.org.uk/babyfriendly

## 2. Definitions

| | |
|---|---|
| AREDF | Absent or Reversed End Diastolic Flow (in umbilical artery, seen on antenatal scans) |
| AXR | Abdominal X-Ray |
| BMF | Breast Milk Fortifier |
| CPAP | Continuous Positive Airways Pressure |
| D/C | Discharge |
| DBM | Donor Breast Milk |
| DH | Department of Health |
| ELBW | Extremely Low Birth Weight (birth weight <1000g) |
| FBC | Full Blood Count |
| g | grams |
| IU | International Units |
| IUGR | Intrauterine Growth Restriction |
| IV | Intravenous |
| kcal | kilocalories |
| kg | kilogram |
| LBW | Low Birth Weight (birth weight <2500g) |
| LFT | Liver Function Tests |
| MBM | Maternal Breast Milk |
| mg | milligram |
| ml | millilitre |
| mmol | millimole |
| NBM | Nil By Mouth |
| NEC | Necrotising Enterocolitis |
| NICU | Neonatal Intensive Care Unit |
| NNU | Neonatal Unit |
| PBP | Potentially Better Practice |
| PDA | Patent Ductus Arteriosus |
| PDF | Post Discharge Formula |
| PN | Parenteral Nutrition |
| RCT | Randomised Controlled Trial |
| SD | Standard Deviation |
| TAT | Trans-anastamotic Tube |
| TPN | Total Parenteral Nutrition |
| U&E | Urea and Electrolytes |
| VLBW | Very Low Birth Weight (birth weight <1500g) |
| VON | Vermont Oxford Network |


Disclaimer: It is your responsibility to check against StaffNet that this printout is the most recent issue of this document

## 3. Roles and Responsibilities

**BREAST-FEEDING AND LACTATION SUPPORT**

- All staff: awareness of Trust Policy and NNU Guidelines
- 'Breast-feeding babes' – Lead Sandy Jackson: expert guidance for mothers breast-feeding on the post-natal wards
- NNU lactation support team – Lead Jess Macfarlane: expert guidance for mothers breast-feeding and/or expressing milk in NNU

**PARENTERAL NUTRITION**

- All staff: awareness of need for PN in high risk infants
- Nursing staff: awareness of location of 'stock' PN in NNU and knowledge and skills for PN administration appropriate to nursing skill level
- Medical staff: awareness of PN supplies available and how to prescribe; awareness of potential complications of PN and how to avoid
- Pharmacists: Amanda Bevan and Zoe Lansdowne: expertise in detailed composition of PN solutions and provision of PN in different situations on NNU

**ENTERAL NUTRITION**

- All staff: support for mothers in choice of feeding
- All staff: awareness of choices for enteral nutrition: maternal breast milk / breast-feeding; donor breast milk / milk bank; standard infant formula; formulas for preterm infants; special formulas for infants with specific gut or feeding problems
- Neonatal Dietitian (Anita Emm): expert knowledge of composition of breast milk and alternatives and guidance on making appropriate choices
- Surgical team: expert knowledge on potential feeding challenges in infants with congenital or acquired abnormalities of the gut, particularly following surgery.

**FEEDING DIFFICULTIES**

- All staff: awareness of common feeding difficulties of preterm infants and those with neurological complications
- Speech and language therapist: expert knowledge of structure and function of upper gastro- intestinal tract and how to optimise feeding potential of vulnerable babies

**GROWTH MONITORING**

- All staff: Awareness of importance of making accurate and regular measurements and plotting them on appropriate charts to monitor growth
- Nursing staff: Weigh babies at intervals as indicated by clinical condition (ideally three times per week)
- Medical and Nursing staff: Measure head circumference and length at intervals as indicated by clinical conditions (ideally head circumference at least weekly and length at least fortnightly)
- Medical and Nursing staff: Plot growth measurements on appropriate chart weekly (provided competent to do so)

**SPECIAL CASES**

- Neonatal Nutrition Team: Will review high risk or complex patients on weekly nutrition ward round

University Hospital Southampton NHS
NHS Foundation Trust

## 4. Related Trust Documents

Donor Breast Milk Guideline (to be found at: http://staffnet/TrustDocsMedia/DeptDivSpecific/DivC/WomenNewborn/NeonatalUnit/NeonatalGuidelines/DonerBreastMilkGuideline/DonorBreastMilkGuideline.doc)

Breastfeeding care pathway (on Neonatal Unit Guidelines on Unit Desktop PCs)

Vitamins and supplements guideline (on Neonatal Unit Guidelines on Unit Desktop PCs)

Parenteral Nutrition Guidebook, 4th edition (Hard copies in nurseries on Neonatal Unit)

Princess Anne Breastfeeding Policy (to be found at http://staffnet/TrustDocsMedia/DeptDivSpecific/DivC/WomenNewborn/Obstetrics/ObstetricClinicalGuidelines/BreastfeedingTermInfantsGuideline/BreastfeedingTermInfantsGuideline.doc)

Neonatal Unit Breastfeeding and Formula Feeding Guideline (currently being written)

Neonatal Surgical Clinical Aids (to be found at: http://staffnet/Departments/DivisionC/Womenandnewborn/Neonatalservices/Neonatalsurgery/Neonatalsurgeryclinicalaids/Neonatalsurgeryclinicalaids.aspx)

Central Venous Access Guideline (currently being written)

Naso/Orogastric Tubes in Neonates - the safe placement of: Guidelines (to be found at:http://staffnet/TrustDocsMedia/DeptDivSpecific/DivC/WomenNewborn/NeonatalUnit/NeonatalGuidelines/NasoOrogastricTubesinNeonates-thesafeplace/NasoOrogastricTubesinNeonates-thesafeplacementofGuidelines.DOC)

# 5. Guideline Information

## 1. AIMS AND OBJECTIVES

- To optimise use of breast milk and breast-feeding
- To achieve recommended nutrient intakes
- To achieve postnatal growth and body composition approximating fetal growth.
- To reduce the risk of nutritional deficiency states such as late anaemia of prematurity or metabolic bone disease.
- To reduce the risk of feeding related morbidities such as NEC or cholestasis
- To optimise long term neurodevelopmental outcome.

## KEY PRINCIPLES

- All babies should be measured and have nutritional risk assessment on admission, and weekly during their stay
- Nutrition support should be started early: PN for high risk; enteral feeds for lower risk
- Mother's breast milk is the feed of first choice
- Feed tolerance should be assessed regularly and managed according to algorithms
- Protein intake should be documented and optimised in preterm infants
- High risk babies should be seen each week by the Nutrition Team
- Nutrition and feeding should be discussed in Discharge Planning and documented in the notes

## AUDIT POINTS

- Use of Nutrition Screening Tool, on all NNU admissions (100%)
- Use of growth charts on all NNU admissions (100%)
    - Weight and Head Circumference plot weekly; length plot 2-weekly
- Lactation advice and support by 6 hours for all mothers of VLBW infants
    - 100% - unless mother too ill
- Breastfeeding rates at discharge
- Protein and energy intakes as recommended by Tsang 2005
- Use of nutritional supplements according to Guidelines
- Documentation of Nutrition Plan at discharge (100%)

University Hospital Southampton NHS
NHS Foundation Trust

## 2. ASSESSMENT AND MONITORING

### (i) INITIAL ASSESSMENT

#### a. Growth Measurement

All infants should have weight, length and head circumference measured and plotted on the appropriate growth chart at admission. This information, together with other risk factors detailed below, will identify the degree of 'nutritional risk' – ie risk of becoming malnourished or developing nutrition and feeding related problems. Infants with multiple risk factors should be classified according to their highest individual risk factor. This will guide nutritional care and allow subsequent progress to be monitored.

#### b. Risk assessment – identify level of risk for nutrition and / or feeding-related problems

High risk

- Preterm <28 weeks
- ELBW < 1000g
- Severe IUGR (weight < 2$^{nd}$ centile with AREDF) <35 weeks
- Infant establishing feeds after episode of NEC or GI perforation
- Infants with severe congenital GI malformation: e.g. gastroschisis
- Severe Perinatal hypoxia / ischaemia

Moderate risk

- Preterm 28-31$^{+6}$ weeks, otherwise well
- VLBW 1000 – 1500g
- Moderate IUGR (weight < 9$^{th}$ centile with AREDF) <35 weeks
- Baby on inotropes
- Baby on indomethacin/ibuprofen (NB avoid concomitant treatment with steroids)
- Baby >1500g with illness or congenital anomaly which may compromise feeding
- Symptomatic polycythaemia, with PCV $\geq$ 70%

Low risk

- Preterm 32-36$^{+6}$ weeks, otherwise well
- AREDF / IUGR $\geq$35 weeks
- Term Infants >37 weeks

(ii) ON-GOING ASSESSMENT AND MONITORING

a. GROWTH

i. Weight should be measured at least twice a week, and plotted on CLOSE MONITORING WHO growth chart weekly. More frequent weights required for some babies should be plotted on a daily weight chart

ii. Head circumference should be measured and plotted weekly

iii. Length should be measured and plotted within the first week, and every 2 weeks thereafter.

iv. If a baby is too sick to be weighed and measured so cannot be plotted, mark the bottom of the growth chart at date with a triangle (△) at the day's date.

v. Targets for weight – changes in weight in the early days of life usually reflect fluid balance: aim for weight loss of no more than 10% from birth weight. Once baby is stable and growing, aim for gain of 15-20 grams/kg/day

vi. Head circumference and length: normally expect increase of 0.75 cm/week

b. BIOCHEMISTRY

i. **First week of PN**:
- Full TPN Profile daily (FULL IP MG on eQuest, this includes U&E's, Calcium, magnesium phosphate and LFTs)
- FBC twice weekly

ii. **Second and subsequent week of PN**:
- Full TPN Profile and FBC twice weekly if stable (daily if still unstable)

iii. **Triglycerides** should be measured weekly (ideally Mondays)when on IV lipid

iv. **If on PN for longer than 1 month, then Trace elements (Zn, Cu, Se, Mn – use special blood bottle in Dr's Office) and Vitamins (A, D and E) should be measured monthly. Consider measuring Iron status and clotting**

v. **When on enteral feeds:**
- Infants in the High and Medium risk categories need weekly FBC, U&Es, LFTs and Bone profiles once they are off PN and fully enterally fed. This can be extended to once fortnightly when babies are moved into Special Care.

c. SCREENING

i. A Neonatal Nutrition Screening form should be completed on admission and on Sunday/Monday when the baby has been weighed and measured each week on all babies to identify those requiring nutrition team review

d. NUTRITION TEAM REVIEW

i. Nutrition ward rounds take place on Tuesday mornings from 0900-1100. Nutrition team will see all 'high-risk' babies, and any others identified by nutritional screening on Sunday/Mondays.


Disclaimer: It is your responsibility to check against StaffNet that this printout is the most recent issue of this document

## 3. NUTRIENT REQUIREMENTS

Nutrient requirement for Term and Preterm infants in the first weeks of life are summarised below. The figures shown below are based on the parenteral requirements for the first week, and the enteral requirements for the subsequent weeks (for a full list of parenteral and enteral requirements see Appendix 1).

Term infants – based on intake in 150 ml/kg breast milk; preterm infants based on recommendations in Tsang 2005 unless otherwise stated.

There are no specific guidelines for those babies born over 1.5kg and under term weight (2.5 kg) but it can be anticipated that their nutritional needs will be between those of preterm infants and term infants. Nutritional support should therefore aim to deliver nutrient intakes in this area.

It should be noted that these are just recommendations, and some infants may require more of certain nutrients such as Sodium and Potassium as dictated by the results of blood tests.

| **Nutrient Unit/kg/day** | **Term infant** | **Preterm VLBW 1000-1500g 1st week (parenteral)** | **Preterm VLBW 1000-1500g After 1st week (enteral)** | **Preterm ELBW < 1000g 1st week (parenteral)** | **Preterm ELBW < 1000g After 1st week (enteral)** |
|---|---|---|---|---|---|
| Energy (kcal) | 100 | 60-70 | 110-130 | 75-85 | 130-150 |
| Protein (g) | 1.5-2.1 | 3.5 | 3.4-4.2 | 3.5 | 3.8-4.4 |
| Nitrogen (g) | 0.24-0.34 | 0.56 | 0.54-0.61 | 0.56 | 0.61-0.70 |
| Sodium (mmol) | 1.4 | 2.0-5.0 | 3.0-7.0 | 2.0-5.0 | 3.0-7.0 |
| Potassium (mmol) | 2.0 | 0-2.0 | 2.0-3.0 | 0-2.0 | 2.0-3.0 |
| Calcium (mmol) | 1.25 | 1.5 | 2.5-5.5 | 1.5 | 2.5-5.5 |
| Phosphate (mmol) | 1.3 | 1.5-1.9 | 1.9-4.5 | 1.5-1.9 | 1.9-4.5 |
| Vitamin D IU* | 340 | 40-160 | 800-1000 | 40-160 | 800-1000 |
| Vitamin A IU** | 1150 | 700-1500 | 700-1500 | 700-1500 | 700-1500 |
| Iron (umol) | 17.9 | 0 | 35.8-71.6 | 0 | 35.8-71.6 |

*Vitamin D = dose quoted is total daily dose; ESPGHAN 2010 recommendation for enteral dose for preterm infants; term infants DH Dietary Reference Values 1991 (340 IU = 8.5 mcg Vit D)

**Vitamin A = dose quoted is total daily dose; term infants DH Dietary Reference Values 1991 (1150 IU = 350 mcg of Vitamin A retinol equivalent)

## 4. STANDARD NUTRITION SUPPORT –

**(a) OVERVIEW -** GETTING STARTED - EARLY TPN AND TROPHIC MILK FEEDS

**HIGH RISK / MEDIUM RISK (see flow charts for high [A] and medium risk preterm infants [B])**

- Aim to introduce milk feeds gradually while maintaining calorie and nutrient intake with PN
- Before starting or increasing milk ensure baby is clinically stable and abdomen soft
- Ensure mother has lactation support to start expressing (see breastfeeding care pathway)

**High risk preterm (<28 weeks; <1000g; severe IUGR/AREDFV <35 weeks)**

| | |
|---|---|
| Day 1 | Start Stock Preterm PN at 60-90 ml/kg/day via UVC or long line, as soon as possible unless baby very unstable. Give fresh colostrum as mouth care or as trophic feeds |
| Day 2-3 | Start trophic feeds: MBM 1 ml/kg 2-4 hourly (if no MBM can use DBM- see choice of milk chart); |
| Day 3-7 | Change to Stock Preterm + Sodium PN when 6% weight loss from birthweight [7], additional sodium required, or by day 5, whichever soonest Increase milk by 10-20 ml/kg/day as tolerated (see table); Aim to decrease PN flow rates with feeds only once baby on total fluids of 180ml/kg/day |

**Moderate risk preterm (28-31$^{+6}$ weeks; 1000g <1500g; mod IUGR/AREDFV < 35 weeks)**

| | |
|---|---|
| Day 1 | Start Stock preterm PN at 60-90 ml/kg/day via UVC or long line as soon as possible; if no central access consider peripheral PN |
| Day 1-2 | Start colostrum/milk 1 ml/kg 2 hourly ('see choice of milk' chart) |
| Day 3-7 | Change to Stock Preterm + Sodium PN when 6% weight loss, or by day 5, whichever is sooner. Aim to decrease PN flow rates with feeds only once baby on total fluids of 180ml/kg/day Increase milk by 20-30 ml/kg/day according to clinical condition and tolerance; |

**High / moderate risk term or near-term infants**

All high/moderate risk babies should have a plan for nutrition support on admission and periods greater than 48 hours without protein and micronutrients should be unusual

**Low risk**

| | |
|---|---|
| Day 1 | Commence milk feeds 30-60 ml/kg/day, supplemented by IV fluids if necessary |
| Day 2-7 | Increase milk feeds by 30 ml/kg/day as tolerated |

**NOTES**

- If severely unwell or acidotic, PN may need to be delayed (though contains acetate)
- Babies with HIE undergoing therapeutic hypothermia, may tolerate trophic milk feeds
- For babies with surgical problems , see 'surgical guidelines' – section 6

## 4. (b) PARENTERAL NUTRITION

### i) Indications for PN

- High or Moderate risk infants as described above
- Infants who are NBM and unlikely to achieve adequate milk intake in the next 5 days
- Infants who are not tolerating feeds such that they cannot take full feed volumes for 5 consecutive days

### ii) Starting PN

- In high and moderate risk infants PN should be started as soon as possible as delay can result in significant and cumulative nutrient deficits.
- Birth weight ≤1500g – start as soon as possible after birth
    - Ideally within 6 hours
- Birth weight >1500g – if enteral feeding contra-indicated, start PN by
    - 48 hours in 1500-2500g
    - 72 hours in 2500-3500g if NBM
- Central line insertion (UVC or peripherally inserted central venous line) should be a priority for high and moderate risk infants
- If feeds are stopped on high or medium risk infant for any reason, re-stat PN

### iii) Stock PN

- Infants should be started on Stock PN in the first instance as detailed below:
    - Preterm PN – For preterm infants (<37/40 gestation) where additional sodium is not indicated (ie until 6% weight loss, or day 5 of life)
    - Preterm + Sodium PN- For preterm infants (<37/40 gestation) requiring maintenance sodium. **This should be the PN of choice for the majority of preterm infants after the first few days following birth**, as it contains more protein.
    - Term PN – for Term infants (≥37 weeks gestation) at any point after birth.
- Stock PN comprises an aqueous solution (glucose, amino acids, electrolytes and trace elements) and a lipid solution (**which contains both fat- and water-soluble vitamins**). For adequate nutrition it is **important that the lipid is always given with the aqueous solution** at all times (except when well advanced on enteral feeds - see below).

### iv) Pharmacy made ('bespoke') PN

- Neither PN alone nor unfortified full breast milk feeds fully meet the nutritional needs of preterm infants, so the period when a preterm infant transitions from PN to milk feeds is when they are at highest risk of poor nutrient intakes.
- Stock PN is designed to give the maximum possible nutrition at 130ml/kg/day. **Therefore, pharmacy can make bespoke PN, which provides more nutrition in a smaller volume, should be used whenever a preterm infant is receiving less than 130ml/kg/day of Stock PN**. This will occur whenever a preterm infant is increasing on enteral feeds, is fluid restricted, or receiving other infusions
- Bespoke PN may also be appropriate where infants have electrolyte requirements than cannot be met with Stock PN

**v) Reducing PN as enteral feeds increase**

- **Only once the infant is receiving 180ml/kg/day total fluids should the PN solution be decreased as enteral feeds increase** (unless there is a clinical decision to restrict fluids).
- Once the infant is on 90ml/kg/day enteral feeds, the rate of lipid infusion should be halved, and then stopped when the infant reaches 135ml/kg/day enteral feeds (beware with pharmacy made TPN as this reduction in lipid may have already been done as part of the prescription). Any shortfall in total fluid volume due to the reduction in lipid should be made up by increasing the aqueous PN solution, to allow maximum protein to be delivered to the infant (though do not go above the maximum prescribed rate). This is important when infants are on Stock PN, but for those on bespoke PN, the reduction in lipid may have already been done/accounted for by the pharmacists when the PN was prescribed so may not be necessary (check with the pharmacists first). **Remember that once the lipid is stopped, vitamin intake will be inadequate until Abidec is started.**

**vi) Peripheral PN**

- PN should ideally be given via a central line. However, there are occasions in high nutritional risk infants with difficult access where the benefits of giving PN peripherally may outweigh the risks. Such decisions should be made by the Consultant responsible for the patient.

**vii) Cautions on PN**

SEPSIS - may affect lipid metabolism; measure triglycerides and if >2.8mmol/L consider reducing or stopping IV lipid for 12-24 hours in severely septicaemic baby (remember to restart/increase lipid when sepsis has resolved)

THROMBOCYTOPENIA – high concentration of polyunsaturated fats may impair platelet adhesion: reduce lipid to 1-2 g/kg/day if platelets <50.

CHOLESTATIC JAUNDICE – total and prolonged PN increases the risk, so try to give some enteral feed if at all possible; other risk factors include IUGR, sepsis and short bowel syndrome. Lipid solutions containing fish oil (eg SMOF) can reduce or reverse cholestasis, and should be considered in high risk babies if on PN for 4 weeks or more. Alternate day lipid may also be indicated in this situation, or if altered liver function - discuss with the pharmacists.

## 4 (c) ENTERAL NUTRITION

i. **Starting feeds** – see section 4(a) for guidance. Before starting feeds ensure baby is clinically stable and abdomen soft. In high-risk infants trophic feeding should be started within the first 72 hours if at all possible to minimise intestinal mucosal atrophy, and continued until ready to progress.

ii. **Choice of milk** – Mother's breast milk is almost always the feed of first choice, unless contraindicated by maternal illness or drugs. If no maternal milk available pasteurised donor breast may be used for high risk babies (parental consent required) in accordance with the DBM guideline. Preterm formula (LBW/Aptamil Preterm) is indicated for infants with gestation <34 weeks, or birth weight <1800 grams; Post discharge formula (Nutriprem 2) is indicated for preterm infants either as sole diet or in addition to breast-feeding from around 36 weeks (or at discharge) up to 6 months corrected. (see Flow Chart D)

iii. **Advancing feeds** – see section 4 (a) for guidance on volumes

- Before starting or increasing milk ensure baby is clinically stable and abdomen soft. Small gastric residuals can be tolerated if baby well. Passage of meconium and then changing stools is an important indication of gut motility. Glycerine suppositories may be useful if no stool passed for 48 hours.
- Feeds can be increased by 10-20ml/kg/day in high-risk, 20-30ml/kg/day in moderate risk and 30 ml/kg/day in low risk babies
- Test for residuals 4-6 hourly
- If baby vomits, or has residuals >25% of the previous 4 hours total feed volume and persisting or increasing examine and assess baby and refer to flow chart C

**iv. Nutritional supplements**

- BREAST MILK FORTIFIER (BMF, see high risk and moderate risk flow charts A and B) - 'multi-component' fortifier provides additional calories (carbohydrate), protein (cows' milk based), minerals and vitamins in a powder which is added to mother's breast milk. It should be more or less routine for babies with birth weight <1500g to receive fortifier once they have tolerated 150 mls/kg/day of MBM for 24 hours, unless significant gut or renal compromise. Blood Urea and albumin levels provide useful markers of protein status. In general, give ½ strength for 24-48 hours and then increase to full strength (2.2g sachet to 50 mls MBM), though it may be preferable to increase the fortifier by ¼s in high risk infants. For some extremely high risk infants it may be prudent to start fortifier when on 120-135 mls/kg/day of MBM and increase strength more gradually as PN is gradually reduced, in order to ensure the baby will be able to achieve enteral nutrient targets before stopping PN.

- Vitamins and Iron – breast milk provides insufficient vitamins (particularly vitamin A and D) for preterm infants, and virtually no iron. Abidec (multivitamins) and Sytron (iron) should be started according to NNU guideline
- Electrolytes and minerals
  - Small doses should be given as boluses, as scheduled on drug chart
  - Sodium : aim to maintain serum sodium 135-145 mmol/L
    If on > 4 mmol/kg/day, add to daily feeds in milk kitchen; if < 4 mmol/kg/day, give as divided bolus drugs (ideally as a four times daily regimen)
  - Phosphate: content of BM is low. Aim to maintain serum inorganic phosphate levels greater than 1.8mmo/L. Usually given as Potassium Acid Phosphate 0.5-2mmol/kg/day. If required as outpatient, may be preferable to use BMF

**v. Nutrition at discharge**

It is important to start discharge-planning well in advance. Breast-feeding at discharge is the preferred goal for all infants. However for preterm infants nutritional supplementation will be required. For those not being breast fed advice has to be given on choice of formula, so for all infants a pre-discharge nutrition assessment should be made and plan documented.

MUM PLANNING TO BREAST FEED

- Ensure lactation support is on-going re feeding technique
- Discuss with Out-reach sister re support at home
- **All preterm infants (<35 weeks) should have Abidec (1 ml) and Sytron (1 ml) daily**
- Assess growth
  - If growth has been good and weight, length and HC are no more than 0.67 SD (ie one centile line) below birth levels, then assess weight gain after 48 hours. If satisfactory can go home breast-feeding
  - If baby has had significant post-natal growth restriction and is >1.33 SD below birth (2 centile lines), discuss with Nutrition team / Dietician and consider discharge on BMF, with Outreach Support
  - For those with modest growth restriction, i.e. between one and two centile line drop, review overall pattern of growth and consider requesting nutrition review and Outreach support.

MUM PLANNING TO FORMULA FEED

- Babies <34 weeks gestation, with birthweight <2kg can be considered for discharge on Post-Discharge Formula (PDF) – 'Nutriprem 2'. This should be continued until 3 to 6 months corrected age.
- ELBW and VLBW babies who have been on LBW formula should be changed to PDF at approximately 36 weeks corrected age, or when beginning to take most feeds by bottle. For those who have had severe extra-uterine growth restriction, continuing with LBW formula to 40 weeks corrected age may be appropriate.
- Babies discharged on PDF should have Abidec 0.6 ml, but not Sytron.
- If changing to term formula, prescribe Abidec 1 ml (continue until at least one year post term) and Sytron1ml (continue until 6 month post term)

SOLIDS **–** can be introduced at 5-8 months REAL AGE (ie not corrected for prematurity)

## 5. MANAGEMENT OF COMMON GUT AND FEEDING PROBLEMS – see flow chart C

a. **Gastric aspirates / residuals** – preterm infants have immature gut motility, and aspirates/residuals and small vomits are not uncommon. Dark green bile stained aspirates, particularly in association with abdominal distension and / or tenderness are a cause for concern. However small milky / yellow aspirates up to 2-3 mls are frequently normal. They can be replaced, and feeds continued.

b. **Abdominal distension** – this is another common feature in preterm infants, due to poor gut motility. It tends to be more common in babies on nasal CPAP, with high volumes of air flowing into the upper airway and oesophagus. Tenderness, or systemic symptoms and signs such as apnoea, tachycardia or temperature instability should raise concern. If baby is otherwise well, a small glycerine suppository may help to stimulate peristalsis, and enable feeds to be continued.

c. **Suspected NEC** – classical features are blood and mucous in stools, bile stained aspirates and abdominal tenderness. Systemic signs such as tachycardia and hypotension occur in severe NEC. X-ray might show intramural gas (‘pneumatosis coli’), dilated loops of bowel, free air, or a ‘gas-less’ bowel. In suspected NEC feeds should be stopped, and urgent attention paid to supporting ventilation, circulation and fluid balance.

d. **Suspected GOR** – mild milk reflux is common in newborn babies, including those born preterm and is usually self-limiting. It is rarely the cause of significant cardio-respiratory disturbance. However, apnoea and bradycardia are common in preterm babies and may occur in association with feeds. Try to avoid using gaviscon in babies who are having fortified MBM as the milk becomes excessively thick.

e. **Suspected Food Protein Intolerance** – food protein (e.g. cow’s milk protein) intolerance can occur in young infants either breast fed or formula fed. Symptoms may include severe regurgitation, vomiting, constipation, peri-anal rash, blood in stools and iron deficiency anaemia. Non-intestinal features may include skin rash – atopic eczema, and colic. If this is thought to be the cause of symptoms, it is recommended that cow’s milk protein be excluded from diet. If breast feeding, mother should exclude both cows’ milk and egg products from her diet for two weeks, while continuing to breast feed. Formula fed infants should be tried on amino acid formula. If improvement is seen, a staged reintroduction should be carried out. If no improvement is seen on definite exclusion diet, food protein intolerance is unlikely. If exclusion diet is difficult to maintain, a trial of amino-acid formula may be breast fed infants. See review by Vandenplas et al.[8]

## 6. MANAGEMENT OF BABIES WITH SURGICAL BOWEL CONDITIONS WHICH MAY COMPROMISE NUTRITION

Information has been extracted from the NEONATAL SURGERY CLINICAL AIDS on SUHTranet:

(http://staffnet/Departments/DivisionC/Womenandnewborn/Neonatalservices/Neonatalsurgery/Neonatalsurgeryclinicalaids/Anorectalmalformations.aspx)

This website should be checked to ensure that the most up to date version of the guidance is used.

### GASTROSCHISIS

All babies with gastroschisis will require TPN.

For those treated with a Medicina Silo insertion at the cot-side a percutaneous long line should be sited on the Neonatal Unit but line insertion should ideally be delayed until after gut manipulation has ceased, i.e. once the silo has been removed and the defect closed, to reduce the chance of line colonisation. The median time to closure is 4 days. If it is felt that TPN should be commenced before this time then this can be given via peripheral cannula. In babies in whom it is thought there may be a delay in defect closure it may be better to proceed with line insertion prior to closure. As some gastroschisis babies may go on to have intestinal failure and require long term central venous access, central lines should only be inserted by staff with considerable experience of line insertion so as to avoid loss of suitable veins.

If the baby is taken to theatre for primary closure or surgical silo creation a percutaneous long line can be inserted in theatre at the time if someone with the appropriate expertise is available.

Duration of TPN may vary from 10 days to 6 weeks with a mean of 3 weeks. In rare cases gut function may be impaired for many months.

### DUODENAL ATRESIA

A trans-anastamotic tube (TAT) can be placed during surgery, which allows feeding into the jejunum. A naso/orogastric tube will also be required for gastric decompression. Usually a 6Fr enteral feeding tube is placed nasojejunally and an 8Fr nasogastric tube placed down the other nostril. In preterm babies this may produce problems due to obstruction to both nostrils. In this situation it may be better to pass an orogastric 8Fr tube and leave one nostril patent.

Poor duodenal contractility may delay normal oral feeding for as long as 3 weeks. This may be overcome by transanastamotic feeding although there is evidence that this may delay eventual oral feeding. It is NOT usually necessary to place a long line or commence TPN because of the use of TAT feeding. Duration of admission is about 7 - 10 days but may be longer if motility is very delayed.

### EXOMPHALOS

Nutritional support: Most babies who have undergone primary closure will tolerate enteral feeding soon and not need TPN. Most babies with a silo will require a long line and TPN

**MECONIUM ILEUS**

Feeding may start when gut recovery from surgery allows. Usually start on MBM or standard formula feed grading up slowly. Feed may need to change to hydrolysed formula if weight gain inadequate on breast milk or standard formula. Occasionally TPN is needed.

80-90% of babies with MI are deficient in pancreatic enzymes, and supplementation with 'Creon®' may be required. Further details are provided in Surgical Clinical Aids and treatment will usually be guided by advice from the CF team

**OESOPHAGEAL ATRESIA and TRACHEO-OESOPHAGEAL FISTULA**

A trans-anastomotic tube (TAT) nasogastric tube will be placed at time of surgery and feeding usually commences via the TAT at 48hrs post-op. If the TAT falls out do not re-pass as this may perforate the anastomosis. Consult the surgical team immediately.

Oral feeding normally starts between 3 and 5 days post-op at the discretion of the surgical team.

Gastro-oesophageal reflux prophylaxis: some surgeons use ranitidine post-op for 3 - 6 months. Others do not.


Disclaimer: It is your responsibility to check against StaffNet that this printout is the most recent issue of this document

## 7. FLOW CHARTS

### a. Starting and Increasing Feeds- HIGH RISK INFANTS

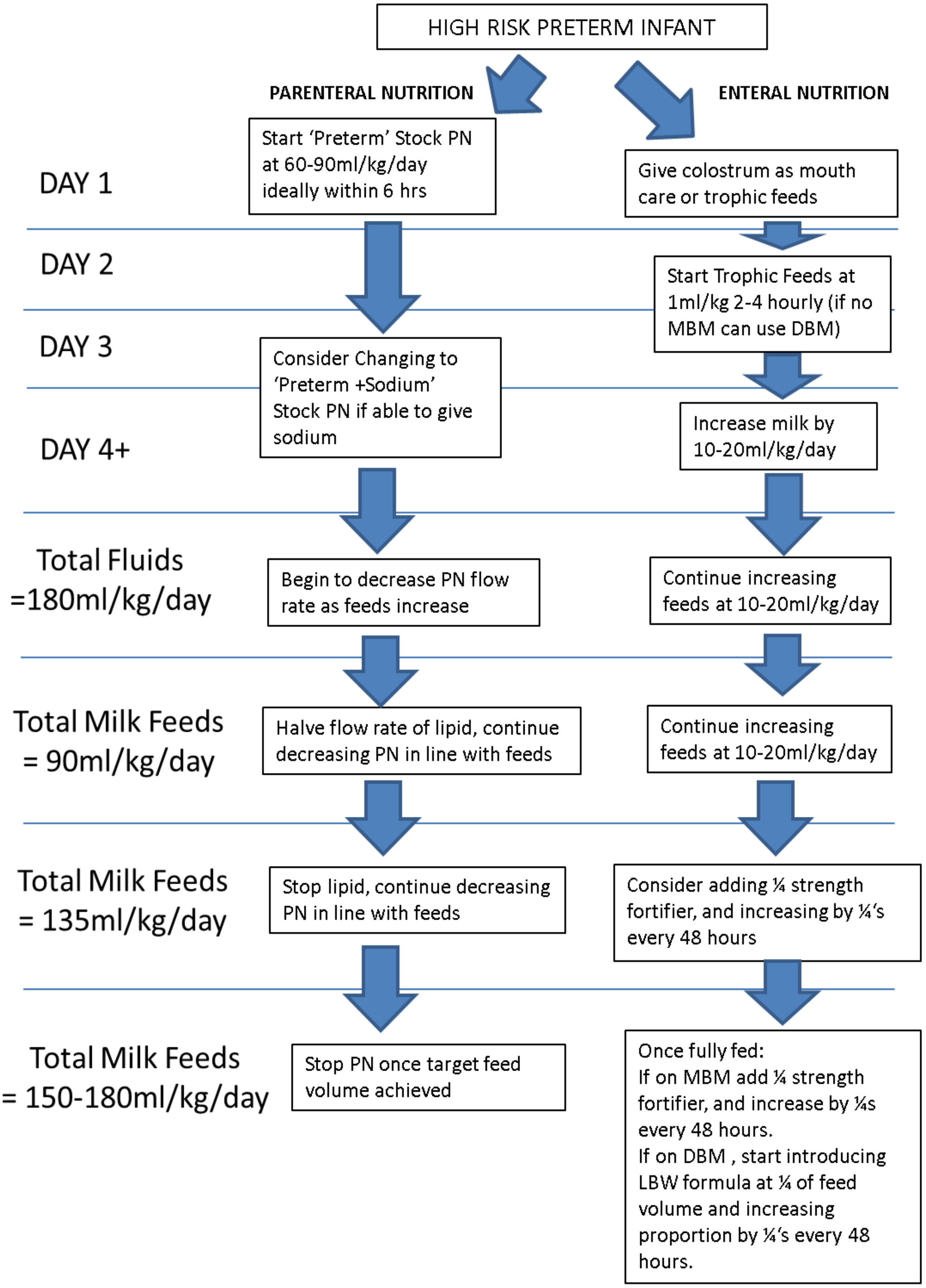



Disclaimer: It is your responsibility to check against StaffNet that this printout is the most recent issue of this document

b. **Starting and Increasing Feeds- MODERATE RISK INFANTS**

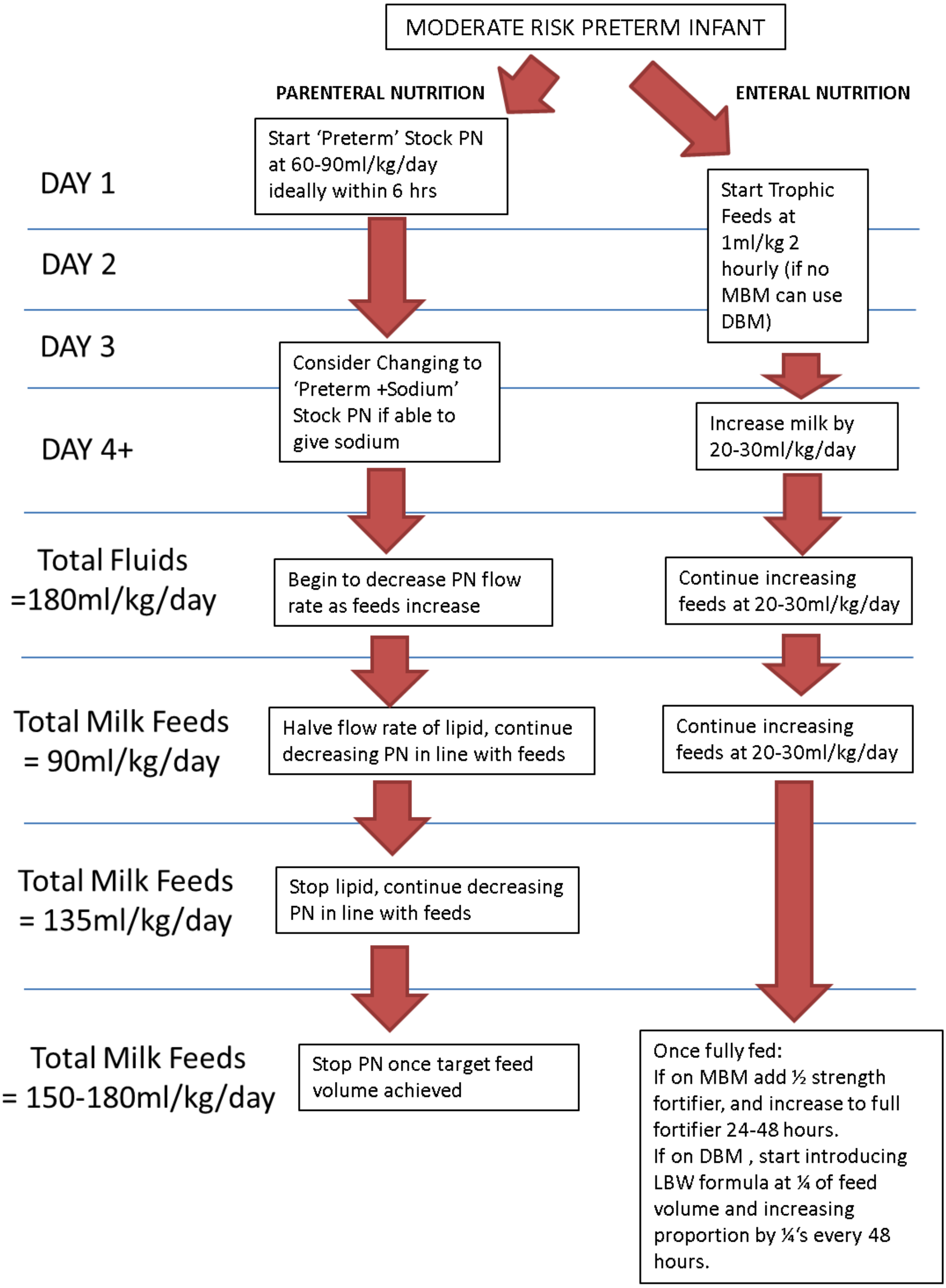



Disclaimer: It is your responsibility to check against StaffNet that this printout is the most recent issue of this document

University Hospital Southampton NHS
NHS Foundation Trust

## b. Management of common feed-related problems

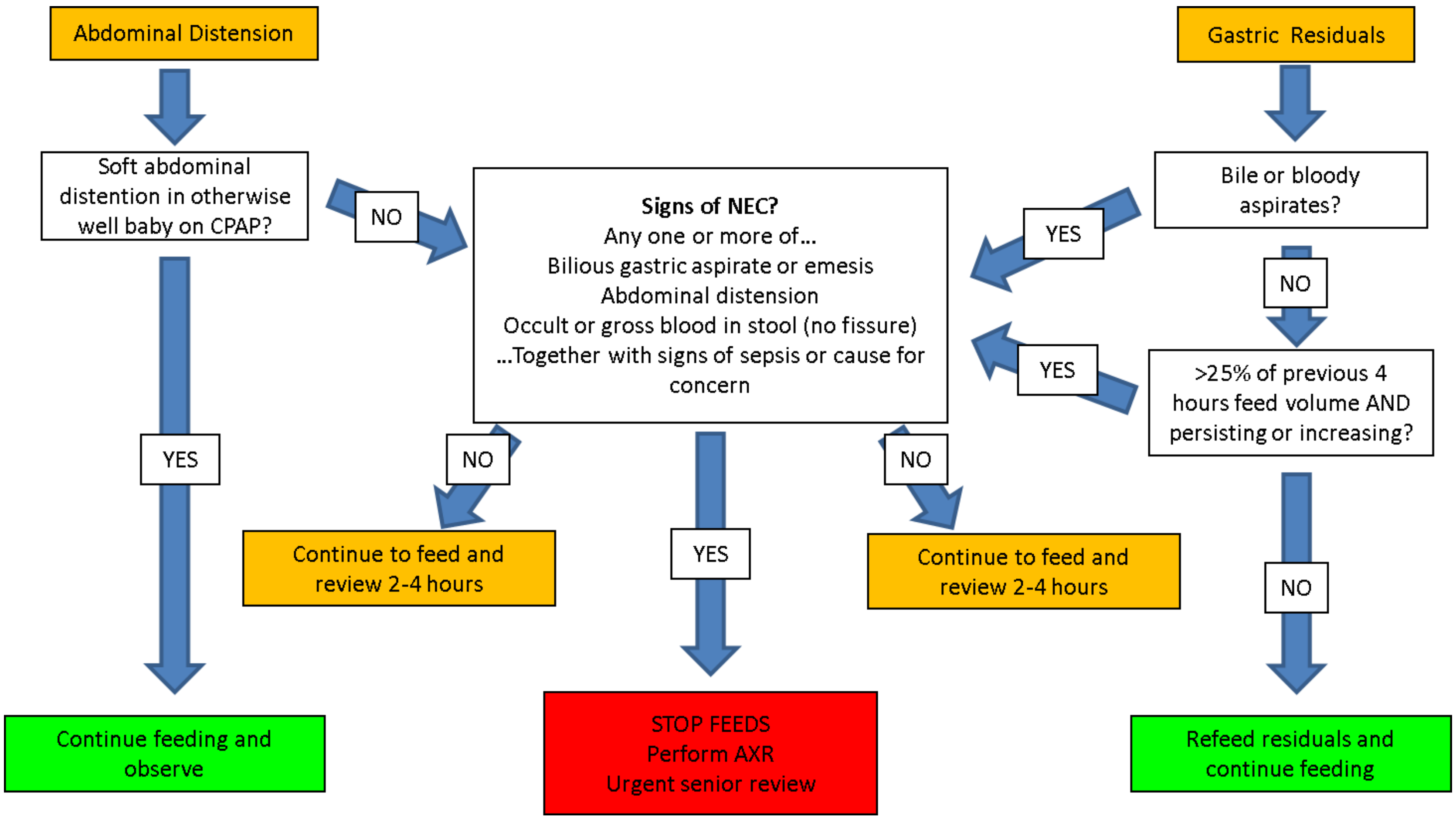

## c. Choice of Milk

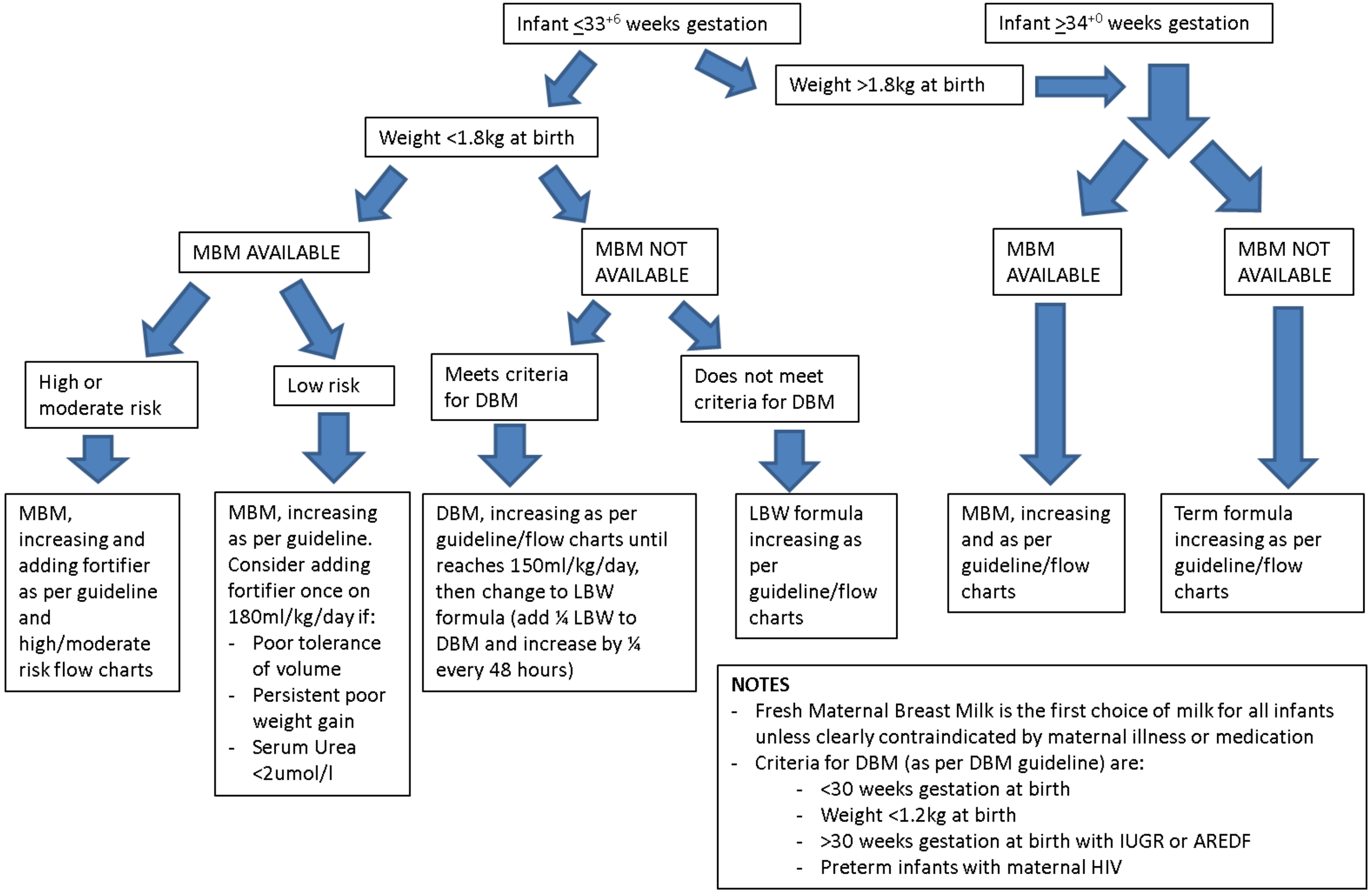


**NOTES**

- Fresh Maternal Breast Milk is the first choice of milk for all infants unless clearly contraindicated by maternal illness or medication
- Criteria for DBM (as per DBM guideline) are:
  - <30 weeks gestation at birth
  - Weight <1.2kg at birth
  - >30 weeks gestation at birth with IUGR or AREDF
  - Preterm infants with maternal HIV

## 8. TABLES

a. Starting and Increasing Feeds

i. High Risk Infants (based on increases of 10-20ml/kg/day)

| Weight (kg) | Start at (hourly) | Start at (2 hourly) | Increase hourly feed volume by* | Increase 2hourly feed volume by |
|---|---|---|---|---|
| less than 0.6 | N/A | 0.5 | 0.25ml every 24 hours | 0.5ml every 24 hours |
| 0.6-0.9 | 0.5 | 1 | 0.5ml every 24 hours | 1ml every 24 hours |
| 0.9-1.2 | 0.75 | 1.5 | 0.5ml every 12 hours | 1ml every 12 hours |
| 1.2-1.5 | 1 | 2 | 0.5ml every 8 hours | 1ml every 8 hours |
| 1.5-1.8 | 1.25 | 2.5 | 0.5ml every 6 hours | 1ml every 6 hours |
| 1.8-2 | 1.5 | 3 | 1ml every 12 hours | 2ml every 12 hours |

ii. Moderate Risk Infants (based on increases on 20-30ml/kg/day)

| Weight (kg) | Start at (hourly) | Start at (2 hourly) | Increase hourly feed volume by:* | Increase 2hourl feed volume by: |
|---|---|---|---|---|
| 1.0-1.2 | 1 | 2 | 0.5ml every 6 hours | 1ml every 6 hours |
| 1.2-1.6 | 1.5 | 3 | 1ml every 12 hours | 2ml every 12 hours |
| 1.6-2.0 | 2 | 4 | 1ml every 8 hours | 2ml every 8 hours |
| 2-2.4 | 2.5 | 5 | 1ml every 6 hours | 2ml every 6 hours |
| 2.4 and above | 3 | 6 | 1.5ml every 8 hours | 3ml every 8 hours |

*Note that this refers to the actual feed **volume** based on 1 hourly feeds. Therefore if baby is 2 hourly fed then multiply the amount on this table by 2 to give the increase on the feed volume, if on 3 hourly feeds multiply by 3 and so on.

University Hospital Southampton NHS
NHS Foundation Trust

| Nutrient / Fluid Name | Preterm Stock PN | Preterm + Sodium Stock PN | Term Stock PN | Stock Lipid | Dextrose 10% | MBM/DBM | MBM with Full Fortifier* | Neocate LCP | Peptijunior | LBW Formula (Aptamil Preterm) | Post D/C Formula (Nutriprem 2) | Term formula | Infantrini |
|---|---|---|---|---|---|---|---|---|---|---|---|---|---|
| **Energy (kcal)** | 63.0 | 59.8 | 70.2 | 166.7 | 40.0 | 69.0 | 85.0 | 71.0 | 66.0 | 80.0 | 75.0 | 66.0 | 100.0 |
| **Protein (g)** | 2.3 | 2.8 | 2.5 | 0 | 0.0 | 1.3 | 2.5 | 2.0 | 1.8 | 2.6 | 2.0 | 1.3 | 2.6 |
| **Carbohydrate (g)** | 12.1 | 11.0 | 13.5 | 0 | 0.0 | 7.2 | 10.0 | 8.1 | 6.8 | 8.4 | 7.4 | 7.3 | 10.3 |
| **Fat (g)** | 0 | 0 | 0 | 16.7 | 0.0 | 4.1 | 4.1 | 3.5 | 3.5 | 3.9 | 4.0 | 3.5 | 5.4 |
| **Sodium(mmol)** | 0.0 | 4.3 | 2.8 | 0.1 | 0.0 | 0.7 | 2.2 | 0.8 | 0.9 | 3.0 | 1.2 | 0.7 | 1.1 |
| **Potassium (mmol)** | 2.4 | 1.7 | 1.9 | 0 | 0.0 | 1.5 | 2.1 | 1.6 | 1.7 | 2.1 | 2.0 | 1.6 | 2.4 |
| **Calcium(mmol)** | 0.8 | 1.0 | 0.9 | 0 | 0.0 | 0.8 | 2.5 | 1.2 | 1.2 | 2.3 | 2.2 | 1.2 | 2.0 |
| **Phosphorous (mmol)** | 1.0 | 2.2 | 0.9 | 1.5 | 0.0 | 0.5 | 1.7 | 1.1 | 0.9 | 2.0 | 1.5 | 0.9 | 1.3 |
| **Iron (umol)** | 0.0 | 0.0 | 0.0 | 0.0 | 0.0 | 1.3 | 1.3 | 18.8 | 13.8 | 25.1 | 17.9 | 9.5 | 21.5 |
| **Vitamin A (IU)** | 0.0 | 0.0 | 0.0 | 3910.0 | 0.0 | 213.0 | 985.6 | 264.0 | 173.2 | 599.4 | 269.7 | 183.2 | 333.0 |
| **Vitamin D (IU)** | 0.0 | 0.0 | 0.0 | 680.0 | 0.0 | 0.0 | 200.0 | 51.0 | 52.0 | 120.0 | 68.0 | 48.0 | 68.0 |
| **Volume (ml/kg) required to reach recommended protein intake (ELBW infants)** | 152 | 125 | 140 | Contains no protein | Contains no protein | 292 | 152 | 195 | 211 | 146 | 190 | 292 | 146 |

b. Nutrient Content of Commonly Used Products per 100ml

Typical Values are used and are correct at 18/10/2011

*Based on Cow and Gate Nutriprem Breast Milk Fortifier

## 9. SUPPORTING INFORMATION

### GUIDELINES AND NUTRITIONAL CARE

There is good evidence from large epidemiological studies such as EPICure that preterm infants often fail to grow adequately, dropping to significantly lower centiles for weight and head circumference at discharge than those which they were born on[9, 10]. There is also evidence that growth failure is also associated with poorer neurodevelopmental outcomes[11]. One significant causative factor for this failure of growth is that these infants receive inadequate nutrition, and there is evidence that they fail to achieve appropriate targets for nutrient intake[12, 13]. Feeding practices across different neonatal units has been shown to be one of the factors responsible for the variability in lengths of stay and the level of postnatal growth restriction seen between different units offering the same level of care[14]. Although there is uncertainty around the definitive practice of nutritional support in preterm infants, there is evidence that standardisation of practice and the use of guidelines is beneficial. A systematic review and meta-analysis by Patole and De Clerk in 2005 showed that the use of standardised feeding regimens reduced rates of NEC, and in the context of the Vermont Oxford Network's 'Potentially Better Practices for Nutrition', the standardisation of practice was shown to reduce the time to start TPN and enteral feeds, improve use of breast milk, reduce lengths of stay and a lower rate of infants being discharges with weights below the 10$^{th}$ centile [4, 15]. Donovan et al studied aspects of nutrient intake and outcomes before and after the introduction of nutrition support guidelines in their NICU, showing significantly earlier initiation of both parenteral and enteral feeding, earlier achievement of full enteral feeding, and earlier regaining of birth-weight after introduction of guidelines[16].

### ASSESSMENT AND MONITORING

Some babies are at higher risk than others of nutritional problems – under-nutrition, feed – related complications or both. Regular assessment of nutritional status and monitoring of growth will help identify infants with greater nutritional needs or a higher risk of poor growth or problems. Preterm infants in particular are at risk and should have their weight, head circumference and length measured at a minimum of once a week [4, 6, 17].
The following are things to consider when assessing nutritional risk

- Term babies with appropriate birth weight have good nutrient stores, designed to support them through the first few days when breast milk volumes are low. They are low risk.

- Preterm babies have low nutrient stores and are born at time of rapid growth – the earlier they are then the bigger the problem and the greater their nutritional risk. This is compounded by immature gut and metabolic function. They are moderate to high risk (depending on gestation) and need early nutrition support.
- Growth restricted babies have less nutritional reserve; they may also have reduced perfusion to the gut before birth and an increased risk of NEC. These babies will therefore be at greater risk compared to babies of a similar gestation.
- Congenital abnormalities such as gastrointestinal abnormalities, facial anomalies and cardiac problems (including PDA and associated treatment) will all affect nutritional status and increase nutritional risk.
- Acquired disorders such as hypoxic-ischaemic injury, sepsis and NEC will impact on the nutrition infants receive and in turn put them at higher risk of poor nutrition.
- Combinations of the any of the above factors will result in a greater overall risk.

## NUTRITIONAL REQUIREMENTS

TERM INFANTS: breast milk provides appropriate nutrients for healthy term babies and breast-feeding should be supported and encouraged. Babies who are not being breast fed should be fed on a standard cows' milk based formula.
PRETERM INFANTS: evidence-based recommendations are available to guide nutrient intakes for preterm infants. The most comprehensive is Tsang 2005 [2], which gives guidelines for parenteral and enteral nutrition support, and specifies requirements for babies <1000g and 1000-1500g birth-weight, during both 'transition' phase (days 2-7 of life) and 'growth phase' (day 7 onwards). ESPHGAN 2010 [1] gives recommendations for enteral intake of fluid and nutrients, though is largely based on the Tsang recommendations. Growth is rapid in the third trimester of fetal life; infants born preterm thus have high requirements for nutrients, but immature physiological capacity to handle them. Breast milk is the optimal first choice for preterm infants' nutrition, however even at high volumes will not provide all adequate nutrients: supplementation with breast milk fortifier or preterm formula may be necessary. The tables in this guideline refer to the Tsang recommendations for energy and protein in VLBW infants and how they compare to typical feeds used in Southampton. Note that only LBW formula milk fed at 150ml/kg/day or fully fortified breast milk fed at 180ml/kg/day is able to achieve the recommended amounts). The full Tsang recommended nutrient intakes are given in Appendix 1. Essentially, the less mature, the lower the nutrient stores/reserves, the earlier nutrient provision is required

## STANDARD NUTRITIONAL SUPPORT OF PRETERM AND SICK INFANTS

a. PARENTERAL NUTRITION

i. *Early use of PN*

The VON Potentially Better Practices for nutrition state that TPN should be commenced as early as possible, ideally within the first 24 hours of life [4]. This helps prevent the net nutrient loss and catabolism that occurs when an infant is born prematurely. Significant nutritional deficits have been shown to occur in the first few days (up to 2 weeks) after birth, so introduction of TPN early is a strategy to help prevent this [12]. There is also good evidence that it promotes anabolism, prevents the loss of protein mass, improves calorie intakes, can improve growth and is safe [3, 18-21].

ii. *Protein intake*

As described above, nutrient delivery in high risk groups is challenging, and the delivery of protein and energy early in life often fails to meet recommended targets. Whilst intravenous glucose given early on will meet energy needs in many cases, it contains no protein, which can only be administered using TPN or milk feeds. Therefore, in high risk infants who cannot be fully fed quickly, it is vital to give the largest amount of protein possible as TPN, as early as possible to try and prevent the accumulation of deficits.  In view of this, Stock TPN in Southampton has recently been reformulated to provide higher levels of protein in a smaller volume. Using high protein TPN to deliver higher protein intakes in the first few days of life in preterm infants has recently been shown to have metabolic benefits in addition to the prevention of catabolism, including a reduction in hyperglycaemia and insulin use [22], and a significant reduction non-oliguric hyperkalaemia [23].

iii. *Peripheral vs central PN*

It is generally accepted that is preferable to given TPN via a percutaneous central venous catheter ('long line') than via a peripheral cannula, in view of the decreased risk of extravasation, the difficulty associated in obtaining repeated peripheral access in preterm infants, and the ability to give higher concentrations of glucose and potassium. Central lines on the other hand have the disadvantage of the risk of catheter related infections. A Cochrane review in 2007 concluded that central TPN was not associated with an increased risk of infection compared to peripheral TPN, and there was some evidence that central TPN resulted in a smaller number of catheters/cannulas per infant required to deliver the TPN, together with improved nutrient delivery [24]. However, it also concluded that there was no significant

difference in adverse events (including extravasation) when comparing central to peripheral TPN. Therefore, whilst TPN should be given centrally wherever possible, peripheral TPN should be considered in some individual cases where there is significant nutritional risk and a delay or difficulty in obtaining central access [3].

iv. *Monitoring and Complications*

Careful monitoring of patients whilst on TPN is important to ensure appropriate and adequate nutrition, and to identify potential complications, including liver disease, metabolic bone disease and catheter-related infection. Current recommendations regarding monitoring have been laid out by ESPGHANs guidelines on paediatric parenteral nutrition[3]., and can be found in the NNU Parenteral Nutrition Guidebook

b. ENTERAL FEEDING

i. *Choice of milk*

There is good evidence that maternal breast milk (and to some extent donor breast milk) is protective against NEC, so breast milk should be the food of first choice [25-30]. Ideally this should be the mother's own fresh colostrum. All mothers of preterm infants should have lactation support, and help with expressing within 6 hours of birth (ideally within half an hour according to current WHO recommendations)[5]. If no maternal milk available by 48 hours and the baby is ready for milk, consent should be sought to use DBM. However, as DBM is a limited resource and there is evidence it contains fewer nutrients than mother's own breast milk, DBM should be reserved only for the purposes of establishing feeds in high risk infants, as laid out in the DBM guideline). Where breast milk cannot be used, preterm infants should receive a specialist high calorie and high protein formula ('LBW formula')[31-33].Preterm formulas are designed to meet the basic nutritional requirements of most preterm infants when fed between 150 and 180ml/kg. Preterm formulas can be used as soon as commencement of enteral feeding is recommended. Term formulas should not be used as they fail to meet the nutritional needs of premature infants. There is no evidence to support the use of term elemental/semi elemental formulas in the early stages of feeding unless there is a compelling clinical reason to do so.

ii. *Starting Feeds*

The objective of early feeding is to stimulate gut maturation, motility and hormone release. As starvation leads to atrophy of the gut, withholding feeds

may render subsequent feeding less safe and protract the time to reach full enteral feeding [34]. No work has yet addressed whether initial feeds should be exclusively breast milk (mother's own or donor) or whether initial feeds should be delayed if only formula is available. However most evidence suggests that any enteral feed given early may be better than gut starvation [35].

Trophic feeding is defined as small volumes of enteral feeds up to 24 mls/kg/day given to promote gut function It has been shown to prevent changes of starvation in gut mucosa, but a systematic review of 9 trials of trophic feeds vs withholding feed, including 754 infants, did not find any difference in overall feed tolerance, weight gain or rates of NEC [36].

Due to concerns about NEC, commencement of enteral feeds is sometimes delayed in preterm infants. A Cochrane review of early vs delayed introduction of progressive enteral feeds did not show an increase in NEC with early feeds, but despite almost 1000 babies in 5 RCTs the conclusion was that data was insufficient [37]. The ADEPT trial randomised 404 preterm, growth-restricted babies to early feeds (start day 2) or late feeds (start day 6): the early group achieved full feeding earlier, required less PN and had less cholestasis, and no difference was seen in incidence of NEC [38]. There is thus no evidence to support delaying feeds; there is a lack of good evidence to guide feeding policy in babies on inotropes and ibuprofen.

iii. *Rate of advancing feeds*

In standard risk infants a rate of increase of 30ml/kg/day is reported safe, whereas data is more limited in the high risk infant. Evidence points towards several days of trophic feeds followed by a rate of increase of 10-20ml/kg/day. There should be a low threshold for withholding stepped increases secondary to tolerance concerns in the high risk infants. There is limited data on this. A Cochrane review [39] including 4 RCTs and 496 babies, considered increase of up to 24 mls/kg/day as slow, and 25 or greater mls/kg/day as rapid. More rapid increase was associated with earlier tolerance of full feeds and faster weight gain, and no difference in NEC, but numbers were too small to make definite conclusions. This topic is being considered by NIHR for a multi-centre UK trial at present.

iv. *Nutritional Supplements:*

As mentioned above, the nutritional needs of preterm infants are greater than infants born at term, and as such breast milk is adequate to meet those

needs [2]. In order to maintain the benefits of breast milk whilst optimising the nutritional status and growth of preterm infants' single multicomponent fortifiers (BMF) have been developed.

Concerns with the use of BMFs include tolerance and their effects to increasing osmolality and in turn the risk of NEC. Most studies have found no significant problems with the tolerance of fortified EBM [40], and a recent review of published evidence found no link between the relatively small increases in osmolality caused by the addition of fortifier to breast milk and NEC [41]. A Cochrane review concluded that the use of BMFs can lead to short term improvements in weight, length and head circumference and that while it is unlikely that further comparative studies with breast milk alone are to take place it recommends further research seeks to evaluate long term outcomes of BMF therapy and identify the optimum composition of BMF products [42].

Recommendations made in 2010 by ESPGHAN stated that the feed of choice for preterm infants (<1800g) was mother's own breast milk supplemented with BMF, or special preterm formula if breast milk not available [1].

v. *Nutrition at Discharge:*

Preterm infants are often discharged home with growth below that expected according to their birth centile. A review by ESPGHAN in 2006 looking at the evidence for feeding preterm infants after discharge recommended that infants discharged with an appropriate weight for their corrected gestational age should be discharged either breast feeding (where breast fed) or on regular formula (where formula fed). However, they also concluded that preterm infants discharged with a subnormal weight for their corrected gestation age should receive fortifier in addition to breast milk (where breast fed) or on special high energy/protein preterm infant formula (where formula fed) [43]. Recently, a Cochrane review looked at this in more detail, addressing the question of whether using fortifier in breast fed preterm infants after discharge improved growth. It concluded that using fortifier after discharge improved growth in infancy, though the evidence was limited [44].

## 6. References


1. Agostoni, C., et al., *Enteral nutrient supply for preterm infants: commentary from the European Society of Paediatric Gastroenterology, Hepatology and Nutrition Committee on Nutrition.* J Pediatr Gastroenterol Nutr, 2010. **50**(1): p. 85-91.
2. Tsang, R.C., *Nutrition of the preterm infant*. 2nd ed. ed. 2005, Cincinnati: Digital Educational Publishing. viii, 427 p.
3. Koletzko, B., et al., *1. Guidelines on Paediatric Parenteral Nutrition of the European Society of Paediatric Gastroenterology, Hepatology and Nutrition (ESPGHAN) and the European Society for Clinical Nutrition and Metabolism (ESPEN), Supported by the European Society of Paediatric Research (ESPR).* J Pediatr Gastroenterol Nutr, 2005. **41 Suppl 2**: p. S1-87.
4. Kuzma-O'Reilly, B., et al., *Evaluation, development, and implementation of potentially better practices in neonatal intensive care nutrition.* Pediatrics, 2003. **111**(4 Pt 2): p. e461-70.
5. WHO, *Management and support of infant feeding in maternity facilities*, in *Infant and young child feeding : model chapter for textbooks for medical students and allied health professionals.* 2009, World Health Organisation. p. 29-36.
6. Edmond, K. and R. Bahl, *Optimal feeding of low-birth-weight infants*. 2006, World Health Organisation.
7. Hartnoll, G., P. Betremieux, and N. Modi, *Randomised controlled trial of postnatal sodium supplementation on body composition in 25 to 30 week gestational age infants.* Arch Dis Child Fetal Neonatal Ed, 2000. **82**(1): p. F24-8.
8. Vandenplas, Y., et al., *Guidelines for the diagnosis and management of cow's milk protein allergy in infants.* Arch Dis Child, 2007. **92**(10): p. 902-8.
9. Wood, N.S., et al., *The EPICure study: growth and associated problems in children born at 25 weeks of gestational age or less.* Archives of Disease in Childhood Fetal & Neonatal Edition, 2003. **88**(6): p. F492-500.
10. Ehrenkranz, R.A., et al., *Longitudinal growth of hospitalized very low birth weight infants.* Pediatrics, 1999. **104**(2 Pt 1): p. 280-9.
11. Ehrenkranz, R.A., et al., *Growth in the neonatal intensive care unit influences neurodevelopmental and growth outcomes of extremely low birth weight infants.* Pediatrics, 2006. **117**(4): p. 1253-61.
12. Embleton, N.E., N. Pang, and R.J. Cooke, *Postnatal malnutrition and growth retardation: an inevitable consequence of current recommendations in preterm infants?* Pediatrics, 2001. **107**(2): p. 270-3.
13. Martin, C.R., et al., *Nutritional practices and growth velocity in the first month of life in extremely premature infants.* Pediatrics, 2009. **124**(2): p. 649-57.
14. Cooke, R.J., S.B. Ainsworth, and A.C. Fenton, *Postnatal growth retardation: a universal problem in preterm infants.* Arch Dis Child Fetal Neonatal Ed, 2004. **89**(5): p. F428-30.
15. Patole, S.K. and N. de Klerk, *Impact of standardised feeding regimens on incidence of neonatal necrotising enterocolitis: a systematic review and meta-analysis of observational studies.* Arch Dis Child Fetal Neonatal Ed, 2005. **90**(2): p. F147-51.
16. Donovan, R., et al., *Outcomes of early nutrition support in extremely low-birth-weight infants.* Nutr Clin Pract, 2006. **21**(4): p. 395-400.
17. *Malnutrition- What nurses working with children and young people need to know and do. An RCN position statement*. 2006, Royal College of Nursing.
18. Anderson, T.L., et al., *A controlled trial of glucose versus glucose and amino acids in premature infants.* J Pediatr, 1979. **94**(6): p. 947-51.
19. Ibrahim, H.M., et al., *Aggressive early total parental nutrition in low-birth-weight infants.* J Perinatol, 2004. **24**(8): p. 482-6.
20. Wilson, D.C., et al., *Randomised controlled trial of an aggressive nutritional regimen in sick very low birthweight infants.* Arch Dis Child Fetal Neonatal Ed, 1997. **77**(1): p. F4-11.

21. Van Goudoever, J.B., et al., *Immediate commencement of amino acid supplementation in preterm infants: effect on serum amino acid concentrations and protein kinetics on the first day of life.* J Pediatr, 1995. **127**(3): p. 458-65.
22. Mahaveer, A., C. Grime, and C. Morgan, *Increasing early protein intake is associated with a reduction in insulin-treated hyperglycaemia in very preterm infants.* Arch Dis Child Fetal Neonatal Ed, 2011. **96**(Suppl 1): p. Fa21.
23. Iacobelli, S., et al., *Standardized parenteral nutrition in preterm infants: early impact on fluid and electrolyte balance.* Neonatology, 2010. **98**(1): p. 84-90.
24. Ainsworth, S.B., L. Clerihew, and W. McGuire, *Percutaneous central venous catheters versus peripheral cannulae for delivery of parenteral nutrition in neonates.* Cochrane Database Syst Rev, 2007(3): p. CD004219.
25. Lucas, A. and T.J. Cole, *Breast milk and neonatal necrotising enterocolitis.* Lancet, 1990. **336**(8730): p. 1519-23.
26. McGuire, W. and M.Y. Anthony, *Donor human milk versus formula for preventing necrotising enterocolitis in preterm infants: systematic review.* Arch Dis Child Fetal Neonatal Ed, 2003. **88**(1): p. F11-4.
27. Schanler, R.J., et al., *Randomized trial of donor human milk versus preterm formula as substitutes for mothers' own milk in the feeding of extremely premature infants.* Pediatrics, 2005. **116**(2): p. 400-6.
28. Boyd, C.A., M.A. Quigley, and P. Brocklehurst, *Donor breast milk versus infant formula for preterm infants: systematic review and meta-analysis.* Arch Dis Child Fetal Neonatal Ed, 2007. **92**(3): p. F169-75.
29. Schanler, R.J., *Suitability of human milk for the low-birthweight infant.* Clin Perinatol, 1995. **22**(1): p. 207-22.
30. Henderson, G., M.Y. Anthony, and W. McGuire, *Formula milk versus maternal breast milk for feeding preterm or low birth weight infants.* Cochrane Database Syst Rev, 2007(4): p. CD002972.
31. Premji, S.S., T.R. Fenton, and R.S. Sauve, *Higher versus lower protein intake in formula-fed low birth weight infants.* Cochrane Database Syst Rev, 2006(1): p. CD003959.
32. Atkinson, S.A., et al., *Randomized Trial of Feeding Nutrient-Enriched vs Standard Formula to Premature Infants during the First Year of Life.* Pediatric Research. **45**(4): p. 276A.
33. Lucas, A., F. King, and N.B. Bishop, *Postdischarge formula consumption in infants born preterm.* Arch Dis Child, 1992. **67**(6): p. 691-2.
34. Ziegler, E.E., P.J. Thureen, and S.J. Carlson, *Aggressive nutrition of the very low birthweight infant.* Clin Perinatol, 2002. **29**(2): p. 225-44.
35. King, C., *What's new in enterally feeding the preterm infant?* Arch Dis Child Fetal Neonatal Ed, 2010. **95**(4): p. F304-8.
36. Bombell, S. and W. McGuire, *Early trophic feeding for very low birth weight infants.* Cochrane Database Syst Rev, 2009(3): p. CD000504.
37. Morgan, J., L. Young, and W. McGuire, *Delayed introduction of progressive enteral feeds to prevent necrotising enterocolitis in very low birth weight infants.* Cochrane Database Syst Rev, 2011(3): p. CD001970.
38. Leaf, A., et al., *Early or late enteral feeding for preterm growth-restricted infants? The abnormal Doppler enteral prescription trial.* Archives of Disease in Childhood, 2010. **95**: p. A3.
39. Morgan, J., L. Young, and W. McGuire, *Slow advancement of enteral feed volumes to prevent necrotising enterocolitis in very low birth weight infants.* Cochrane Database Syst Rev, 2011(3): p. CD001241.
40. Lucas, A., et al., *Randomized outcome trial of human milk fortification and developmental outcome in preterm infants.* Am J Clin Nutr, 1996. **64**(2): p. 142-51.
41. Pearson, F., M.J. Johnson, and A.A. Leaf, *Milk Osmolality - Does it Matter?* Arch Dis Child Fetal Neonatal Ed, 2011. **In press**.
42. Kuschel, C.A. and J.E. Harding, *Multicomponent fortified human milk for promoting growth in preterm infants.* Cochrane Database Syst Rev, 2004(1): p. CD000343.

43. Aggett, P.J., et al., *Feeding preterm infants after hospital discharge: a commentary by the ESPGHAN Committee on Nutrition.* J Pediatr Gastroenterol Nutr, 2006. **42**(5): p. 596-603.
44. McCormick, F.M., et al., *Multinutrient fortification of human breast milk for preterm infants following hospital discharge.* Cochrane Database Syst Rev, 2010(7): p. CD004866.

University Hospital Southampton NHS
NHS Foundation Trust

# Appendix 1- Nutritional requirements of Preterm Infants

| | Extremely Low Birth Weight (<1000g) | | | | | | | | | | | | Very Low Birth Weight (<1500g) | | | | | | | | | | | |
|---|---|---|---|---|---|---|---|---|---|---|---|---|---|---|---|---|---|---|---|---|---|---|---|---|
| | parenteral | | | | | | enteral | | | | | | parenteral | | | | | | enteral | | | | | |
| | Day 0 Lower | Day 0 Upper | Trans Lower | Trans Upper | Grow Lower | Grow Upper | Day 0 Lower | Day 0 Upper | Trans Lower | Trans Upper | Grow Lower | Grow Upper | Day 0 Lower | Day 0 Upper | Trans Lower | Trans Upper | Grow Lower | Grow Upper | Day 0 Lower | Day 0 Upper | Trans Lower | Trans Upper | Grow Lower | Grow Upper |
| Energy (kcal) | 40 | 50 | 75 | 85 | 105 | 115 | 50 | 60 | 90 | 100 | 130 | 150 | 40 | 50 | 60 | 70 | 90 | 100 | 50 | 60 | 75 | 90 | 110 | 130 |
| Protein (g) | 2 | 2 | 3.5 | 3.5 | 3.5 | 4 | 2 | 2 | 3.5 | 3.5 | 3.8 | 4.4 | 2 | 2 | 3.5 | 3.5 | 3.2 | 3.8 | 2 | 2 | 3.5 | 3.5 | 3.4 | 4.2 |
| Carbohydrate (g) | 7 | 7 | 8 | 15 | 13 | 17 | 7 | 7 | 8 | 15 | 9 | 20 | 7 | 7 | 5 | 12 | 9.7 | 15 | 7 | 7 | 5 | 12 | 7 | 17 |
| Fat (g) | 1 | 1 | 1 | 3 | 3 | 4 | 1 | 1 | 1 | 3 | 3.2 | 8.4 | 1 | 1 | 1 | 3 | 3 | 4 | 1 | 1 | 1 | 3 | 5.3 | 7.2 |
| Sodium (mmol) | 0 | 1 | 2 | 5 | 3 | 5 | 0 | 1 | 2 | 5 | 3 | 5 | 0 | 1 | 2 | 5 | 3 | 5 | 0 | 1 | 2 | 5 | 3 | 5 |
| Chloride (mmol) | 0 | 1 | 2 | 5 | 3 | 7 | 0 | 1 | 2 | 5 | 3 | 7 | 0 | 1 | 2 | 5 | 3 | 7 | 0 | 1 | 2 | 5 | 3 | 7 |
| Potassium (mmol) | 0 | 0 | 0 | 2 | 2 | 3 | 0 | 0 | 0 | 2 | 2 | 3 | 0 | 0 | 0 | 2 | 2 | 3 | 0 | 0 | 0 | 2 | 2 | 3 |
| Calcium (mmol) | 0.5 | 1.5 | 1.5 | 1.5 | 1.5 | 2 | 0.8 | 2.5 | 2.5 | 2.5 | 2.5 | 5.5 | 0.5 | 1.5 | 1.5 | 1.5 | 1.5 | 2 | 0.8 | 2.5 | 2.5 | 2.5 | 2.5 | 5.5 |
| Phosphorous (mmol) | 0 | 0 | 1.5 | 1.9 | 1.5 | 1.9 | 0.6 | 1.9 | 1.9 | 4.5 | 1.9 | 4.5 | 0 | 0 | 1.5 | 1.9 | 1.5 | 1.9 | 0.6 | 1.9 | 1.9 | 4.5 | 1.9 | 4.5 |
| Magnesium (mmol) | 0 | 0 | 0.2 | 0.3 | 0.2 | 0.3 | 0.1 | 0.3 | 0.3 | 0.6 | 0.3 | 0.6 | 0 | 0 | 0.2 | 0.3 | 0.2 | 0.3 | 0.1 | 0.3 | 0.3 | 0.6 | 0.3 | 0.6 |
| Iron (umol) | 0 | 0 | 0 | 0 | 1.8 | 3.6 | 0 | 0 | 0 | 0 | 35.8 | 71.6 | 0 | 0 | 0 | 0 | 1.8 | 3.6 | 0 | 0 | 0 | 0 | 35.8 | 71.6 |
| Zinc (umol) | 0 | 2.3 | 2.3 | 2.3 | 6.1 | 6.1 | 0 | 15.3 | 6.1 | 18.3 | 15.3 | 45.9 | 0 | 2.3 | 2.3 | 2.3 | 6.1 | 6.1 | 0 | 15.3 | 6.1 | 18.3 | 15.3 | 45.9 |
| Copper (umol) | 0 | 0 | 0 | 0.3 | 0.3 | 0.3 | 0 | 0 | 0 | 2.4 | 1.9 | 2.4 | 0 | 0 | 0 | 0.3 | 0.3 | 0.3 | 0 | 0 | 0 | 2.4 | 1.9 | 2.4 |
| Selenium (nmol) | 0 | 0 | 0 | 16.5 | 19 | 57 | 0 | 0 | 0 | 16.5 | 16.5 | 57 | 0 | 0 | 0 | 16.5 | 19 | 57 | 0 | 0 | 0 | 16.5 | 16.5 | 57 |
| Iodine (nmol) | 0 | 0 | 0 | 8 | 7.9 | 7.9 | 0 | 0 | 0 | 473 | 79 | 473 | 0 | 0 | 0 | 8 | 7.9 | 7.9 | 0 | 0 | 0 | 473 | 79 | 473 |
| Manganese (nmol) | 0 | 0 | 0 | 13.7 | 18.2 | 18.2 | 0 | 0 | 0 | 137 | 13 | 137 | 0 | 0 | 0 | 13.7 | 18.2 | 18.2 | 0 | 0 | 0 | 137 | 13 | 137 |
| Vitamin A (IU) | 700 | 1500 | 700 | 1500 | 700 | 1500 | 700 | 1500 | 700 | 1500 | 700 | 1500 | 700 | 1500 | 700 | 1500 | 700 | 1500 | 700 | 1500 | 700 | 1500 | 700 | 1500 |
| Vitamin D (IU) | 40 | 160 | 40 | 160 | 40 | 160 | 150 | 400 | 150 | 400 | 150 | 400 | 40 | 160 | 40 | 160 | 40 | 160 | 150 | 400 | 150 | 400 | 150 | 400 |
| Vitamin E (IU) | 2.8 | 3.5 | 2.8 | 3.5 | 2.8 | 3.5 | 6 | 12 | 6 | 12 | 6 | 12 | 2.8 | 3.5 | 2.8 | 3.5 | 2.8 | 3.5 | 6 | 12 | 6 | 12 | 6 | 12 |
| Vitamin K (ug) | 0 | 0 | 22 | 22 | 22 | 22 | 0 | 0 | 18 | 22 | 18 | 22 | 0 | 0 | 22 | 22 | 22 | 22 | 0 | 0 | 18 | 22 | 18 | 22 |
| Thiamin (ug) | 200 | 350 | 200 | 350 | 300 | 350 | 180 | 240 | 180 | 240 | 180 | 240 | 200 | 350 | 200 | 350 | 300 | 350 | 180 | 240 | 180 | 240 | 180 | 240 |
| Riboflavin (ug) | 150 | 200 | 150 | 200 | 150 | 200 | 250 | 360 | 250 | 360 | 250 | 360 | 150 | 200 | 150 | 200 | 150 | 200 | 250 | 360 | 250 | 360 | 250 | 360 |
| Vitamin B6 (ug) | 150 | 200 | 150 | 200 | 150 | 200 | 150 | 210 | 150 | 210 | 150 | 210 | 150 | 200 | 150 | 200 | 150 | 200 | 150 | 210 | 150 | 210 | 150 | 210 |
| Folate (ug) | 56 | 56 | 56 | 56 | 56 | 56 | 25 | 50 | 25 | 50 | 25 | 50 | 56 | 56 | 56 | 56 | 56 | 56 | 25 | 50 | 25 | 50 | 25 | 50 |
| Vitamin B12 (ug) | 0.3 | 0.3 | 0.3 | 0.3 | 0.3 | 0.3 | 0.3 | 0.3 | 0.3 | 0.3 | 0.3 | 0.3 | 0.3 | 0.3 | 0.3 | 0.3 | 0.3 | 0.3 | 0.3 | 0.3 | 0.3 | 0.3 | 0.3 | 0.3 |
| Biotin (ug) | 5.8 | 5.8 | 5.8 | 5.8 | 5.8 | 5.8 | 3.6 | 6 | 3.6 | 6 | 3.6 | 6 | 5.8 | 5.8 | 5.8 | 5.8 | 5.8 | 5.8 | 3.6 | 6 | 3.6 | 6 | 3.6 | 6 |
| Pantothenic Acid (mg) | 1.2 | 1.2 | 1.2 | 1.2 | 1.2 | 1.2 | 1.2 | 1.7 | 1.2 | 1.7 | 1.2 | 1.7 | 1.2 | 1.2 | 1.2 | 1.2 | 1.2 | 1.2 | 1.2 | 1.7 | 1.2 | 1.7 | 1.2 | 1.7 |
| Niacin (mg) | 4 | 6.8 | 4 | 6.8 | 4 | 4.6 | 3.6 | 4.8 | 3.6 | 4.8 | 3.6 | 4.8 | 4 | 6.8 | 4 | 6.8 | 4 | 4.6 | 3.6 | 4.8 | 3.6 | 4.8 | 3.6 | 4.8 |
| Vitamin C (mg) | 15 | 25 | 15 | 25 | 15 | 25 | 18 | 24 | 18 | 24 | 18 | 24 | 15 | 25 | 15 | 25 | 15 | 25 | 18 | 24 | 18 | 24 | 18 | 24 |
| Taurine (mg) | 0 | 3.75 | 1.88 | 3.75 | 1.88 | 3.75 | 0 | 9 | 4.5 | 9 | 4.5 | 9 | 0 | 3.75 | 1.88 | 3.75 | 1.88 | 3.75 | 0 | 9 | 4.5 | 9 | 4.5 | 9 |
| Choline (mg) | 0 | 28 | 14.4 | 28 | 14.4 | 28 | 0 | 28 | 14.4 | 28 | 14.4 | 28 | 0 | 28 | 14.4 | 28 | 14.4 | 28 | 0 | 28 | 14.4 | 28 | 14.4 | 28 |
| Carnitine (mg) | 0 | 2.9 | 2.9 | 2.9 | 2.9 | 2.9 | 0 | 2.9 | 2.9 | 2.9 | 2.9 | 2.9 | 0 | 2.9 | 2.9 | 2.9 | 2.9 | 2.9 | 0 | 2.9 | 2.9 | 2.9 | 2.9 | 2.9 |
| Inositol (mg) | 0 | 54 | 54 | 54 | 54 | 54 | 0 | 54 | 32 | 81 | 32 | 81 | 0 | 54 | 54 | 54 | 54 | 54 | 0 | 54 | 32 | 81 | 32 | 81 |

University Hospital Southampton NHS
NHS Foundation Trust

| |
|---|
| Affix Patient Label Here |

# Neonatal Nutritional Screening Tool

*To be completed on admission and weekly (every Monday)*

**Gestation at birth:** **Birth Weight:**

## 1. Assess Growth

| | | | | | |
|---|---|---|---|---|---|
| Current Weight: | | Current Centile: | | Birth Centile: | |
| Current OFC: | | Current Centile: | | Birth Centile: | |
| Current Length: | | Current Centile: | | Birth Centile: | |

## 2. Determine Risk Category

| | | *Tick* |
|---|---|---|
| HIGH RISK | *Any one of:*<br>• Preterm <28 weeks at birth<br>• Extremely Low Birth Weight < 1000g<br>• Severe IUGR (weight < 2nd centile and AREDFV) <35 weeks<br>• Infant establishing feeds after episode of NEC or GI perforation<br>• Infants with severe congenital GI malformation: gastroschisis<br>• Perinatal hypoxia / ischaemia with multi-organ dysfunction | |
| MODERATE RISK | *Any one of:*<br>• Preterm 28-31$^{+6}$ weeks, otherwise well<br>• Very Low Birth Weight 1000 - 1500g<br>• Moderate IUGR (weight < 9th centile and AREDFV) <35 weeks<br>• Baby on inotropes<br>• Baby on indomethacin/ibuprofen<br>• Illness or congenital anomaly which may compromise feeding<br>• Polycythaemia | |
| LOW RISK | *Any one of:*<br>• Preterm 32-36$^{+6}$ weeks, otherwise well<br>• AREDFV / IUGR ≥35 weeks | |
| NO RISK | • Well Term Infant ≥37 weeks | |

## 3. Determine the need for nutrition team review

The nutriton team should review any infant meeting the following criteria:

| | *Tick* |
|---|---|
| • High Risk Infants according to criteria above | |
| • Not regained birth weight by 2 weeks of age | |
| • >15% weight loss at any time | |
| • Weight gain <10g/kg/day from 2 weeks of age onwards | |
| • Drop through 2 centile lines for weight/HC/length | |
| • Intake <150ml/kg/day from 2 weeks of age onwards | |
| • NEC or GI surgery at any time | |

Name of person completing assessement: ____________________ Signature: ____________________

***If completing a first assessment on admission, please place this form in the plastic wallet in the baby's clear plastic nursing folder, next to the nutrition flow charts***

***If completing a a weekly asessment, please place this form in the box outside Room 3 once filled out***

# Nutrition Team Review

Date: ____________

Day: ____________

Gestation at Birth: ____________

Corrected Gestation: ____________

Staff Present:

**Current Clinical Issues:**

**Fluid Intake**

Total Prescribed Fluids: ml/kg/day

Enteral Feed Type: Parenteral Feed Type:

**Nutrient Intake**

**Enteral Feed Provides:**

| | | | |
|---|---|---|---|
| Milk Feeds: | ml/kg/day | kcal/kg/day | g/kg/day Protein |

**Parenteral Feed Provides:**

| | | | |
|---|---|---|---|
| Aqueous PN: | ml/kg/day | kcal/kg/day | g/kg/day Protein |
| Lipid: | ml/kg/day | kcal/kg/day | |
| **Total Intake:** | ml/kg/day | kcal/kg/day | g/kg/day Protein |
| | | | *g/kg/day Lipid* |

*Comments on intake:*

**Bloods**

| | | | |
|---|---|---|---|
| Hb: | Sodium: | Creatinine: | ALP: |
| CRP: | Potassium: | Albumin: | ALT: |
| Other: | Urea: | Bili: | Magnesium: |
| | Calcium (corr): | | Phosphate: |

**Assessment**

**Recommendations**
