# Supplementary material for: Successfully implementing and embedding guidelines to improve the nutrition and growth of preterm infants in neonatal intensive care: a prospective interventional study
Source: BMJ Open. 2017 Dec 6;7(12):e017727. doi: 10.1136/bmjopen-2017-017727 (PMC5728292; doi:10.1136/bmjopen-2017-017727)
Supplement: Supplementary file 2 [file bmjopen-2017-017727supp002.pdf]

Additional File 2: Data tables to accompany Figure 3 (mean daily nutrient intakes across stay) and Figure 4 (growth over stay)

		Mean Daily Energy Intake in kcal/kg/day (95% CI)		Mean Daily Protein Intake in g/kg/day (95% CI)		Mean Daily Energy Intake as a percentage of RRI (95% CI)		Mean Daily Protein Intake as a percentage of RRI (95% CI)	
**Period**	**Degrees of Freedom**	Unadjusted	Adjusted for sex, gestational age and weight at birth	Unadjusted	Adjusted for sex, gestational age and weight at birth	Unadjusted	Adjusted for sex, gestational age and weight at birth	Unadjusted	Adjusted for sex, gestational age and weight at birth
A. Pre-implementation period (January 1st - July 31st 2011)	10190	115.17 (111.79 to 118.54)	114.51 (111.07 to 117.96)	2.88 (2.77 to 2.98)	2.87 (2.76 to 2.98)	105.31 (103.00 to 107.61)	102.42 (100.45 to 104.39)	79.56 (77.35 to 81.77)	79.19 (76.92 to 81.45)
B. Partial implementation period (August 1st – December 31st 2011)	10190	115.77 (112.61 to 118.94)	115.21 (112.00 to 118.42)	3.09 (2.99 to 3.19)	3.09 (2.98 to 3.19)	102.69 (100.50 to 104.88)	100.86 (98.93 to 102.79)	83.53 (81.42 to 85.65)	83.25 (81.10 to 85.40)
C. Main Intervention Period (January 1st - December 31st 2012)	10190	117.87 (115.23 to 120.52)	117.49 (114.82 to 120.16)	3.20 (3.12 to 3.28)	3.20 (3.12 to 3.28)	100.75 (98.95 to 102.54)	100.58 (99.07 to 102.09)	85.70 (83.97 to 87.42)	85.53 (83.78 to 87.28)
D. Post-implementation period (January 1st - June 30th 2013)	10190	120.45 (116.83 to 124.07)	120.25 (116.61 to 123.89)	3.34 (3.23 to 3.46)	3.34 (3.23 to 3.46)	96.50 (94.03 to 98.98)	97.27 (95.18 to 99.36)	86.79 (84.42 to 89.17)	86.82 (84.42 to 89.22)

Detailed Results of the generalized linear model with mixed effects for nutrient intakes across all 4 study periods. (RRI- reasonable range of intake, CI-confidence interval)

	Mean Difference in Daily Energy Intake kcal/kg/day				Mean Difference in Daily Protein Intake g/kg/day				Mean Difference in Daily Energy Intake as a percentage of RRI				Mean Difference in Daily Protein Intake as a percentage of RRI			
**Comparison**	Unadjusted	p value	Adjusted for sex, gestational age and weight at	p value	Unadjusted	p value	Adjusted for sex, gestational age and weight at	p value	Unadjusted	p value	Adjusted for sex, gestational age and weight at	p value	Unadjusted	p value	Adjusted for sex, gestational age and weight at	p value
A vs B	-0.601	0.986	-0.698	0.979	-0.216	0.001	-0.215	0.001	2.612	0.162	1.559	0.536	-3.971	0.006	-4.066	0.005
A vs C	-2.704	0.549	-2.974	0.47	-0.33	<0.001	-0.33	<0.001	4.559	0.007	1.843	0.431	-6.136	<0.001	-6.345	<0.001
A vs D	-5.279	0.143	-5.733	0.101	-0.472	<0.001	-0.473	<0.001	8.802	<0.001	5.149	0.002	-7.232	<0.001	-7.633	<0.001
B vs C	-2.103	0.638	-2.276	0.577	-0.114	0.169	-0.115	0.169	1.947	0.409	0.283	0.994	-2.165	0.283	-2.28	0.242
B vs D	-4.678	0.19	-5.035	0.144	-0.256	0.003	-0.257	0.003	6.19	0.001	3.59	0.058	-3.262	0.163	-3.568	0.113
C vs D	-2.575	0.543	-2.759	0.489	-0.142	0.087	-0.143	0.091	4.243	0.01	3.306	0.031	-1.096	0.837	-1.288	0.766

Pairwise comparison of all study periods using the generalized linear model with mixed effects approach, showing difference between periods. P values <0.05 are highlighted in bold. Unadjusted differences are given together with differences adjusted for sex, gestational age and weight at birth. Tukey's method was used to adjust for multiple comparisons. (RRI- reasonable range of intake)

	Mean Change in Weight SDS from birth (95% Confidence Interval)			Mean Change in Head Circumference from birth (95% Confidence Interval)		
**Period**	**Degrees of Freedom**	**Unadjusted**	**Adjusted for sex, gestational age and weight at birth**	**Degrees of Freedom**	**Unadjusted**	**Adjusted for sex, gestational age and weight at birth**
**A. Pre-implementation period (January 1st - July 31st 2011)**	3628	-0.941 (-1.040 to -0.842)	-0.939 (-1.032 to -0.847)	745	-0.989 (-1.290 to -0.687)	-1.0574 (-1.322 to -0.793)
**B. Partial implementation period (August 1st – December 31st 2011)**	3628	-0.677 (-0.767 to -0.587)	-0.693 (-0.778 to -0.609)	745	-0.819 (-1.089 to -0.548)	-0.908 (-1.153 to -0.662)
**C. Main Intervention Period (January 1st - December 31st 2012)**	3628	-0.476 (-0.556 to -0.397)	-0.510 (-0.583 to -0.437)	745	-0.685 (-0.855 to -0.515)	-0.738 (-0.884 to -0.591)
**D. Post-implementation period (January 1st - June 30th 2013)**	3628	-0.342 (-0.445 to -0.239)	-0.3911 (-0.4865 to -0.2957)	745	-0.571 (-0.807 to -0.335)	-0.645 (-0.851 to -0.434)

Detailed Results of the general linear model with mixed effects for the change in standard deviation scores (SDS) during stay across all 4 study periods.

	Mean Change in Weight SDS from birth				Mean Change in Head Circumference SDS from birth			
**Comparison**	**Unadjusted**	**p value**	**Adjusted for sex, gestational age and weight at birth**	**p value**	**Unadjusted**	**p value**	**Adjusted for sex, gestational age and weight at birth**	**p value**
**A vs B**	-0.264	<0.001	-0.245	<0.001	-0.17	0.823	-0.15	0.83
**A vs C**	-0.465	<0.001	-0.429	<0.001	-0.304	0.305	-0.32	0.155
**A vs D**	-0.599	<0.001	-0.548	<0.001	-0.418	0.14	-0.413	0.077
**B vs C**	-0.201	<0.001	-0.184	<0.001	-0.134	0.796	-0.17	0.582
**B vs D**	-0.335	<0.001	-0.302	<0.001	-0.248	0.508	-0.263	0.363
**C vs D**	-0.134	0.028	-0.119	0.055	-0.114	0.827	-0.093	0.867

Pairwise comparison of all study periods using the general linear model with mixed effects approach, showing difference between periods. P values <0.05 are highlighted in bold. Unadjusted differences are given together with differences adjusted for sex, gestational age and weight at birth. Tukey's method was used to adjust for multiple comparisons. (SDS-standard deviation score)
