# Supplementary material for: Successfully implementing and embedding guidelines to improve the nutrition and growth of preterm infants in neonatal intensive care: a prospective interventional study
Source: BMJ Open. 2017 Dec 6;7(12):e017727. doi: 10.1136/bmjopen-2017-017727 (PMC5728292; doi:10.1136/bmjopen-2017-017727)
Supplement: Author's manuscript [file bmjopen-2017-017727.draft_revisions.pdf]

BMJ Open is committed to open peer review. As part of this commitment we make the peer review history of every article we publish publicly available.

When an article is published we post the peer reviewers' comments and the authors' responses online. We also post the versions of the paper that were used during peer review. These are the versions that the peer review comments apply to.

The versions of the paper that follow are the versions that were submitted during the peer review process. They are not the versions of record or the final published versions. They should not be cited or distributed as the published version of this manuscript.

BMJ Open is an open access journal and the full, final, typeset and author-corrected version of record of the manuscript is available on our site with no access controls, subscription charges or pay-per-view fees (http://bmjopen.bmj.com).

If you have any questions on BMJ Open's open peer review process please email editorial.bmjopen@bmj.com

Successful implementation and embedding of guidelines to improve the nutrition and growth of preterm infants in neonatal intensive care

Journal:	*BMJ Open*
Manuscript ID	bmjopen-2017-017727
Article Type:	Research
Date Submitted by the Author:	12-May-2017

Complete List of Authors:	Johnson, Mark; University Hospital Southampton NHS Foundation Trust and University of Southampton, National Institute for Health Research Southampton Biomedical Research Centre; University Hospital Southampton NHS Foundation Trust, Department of Neonatal Medicine<br>Leaf, Alison; University Hospital Southampton NHS Foundation Trust and University of Southampton, National Institute for Health Research Southampton Biomedical Research Centre; University Hospital Southampton NHS Foundation Trust, Department of Neonatal Medicine<br>Pearson, Freya; University Hospital Southampton NHS Foundation Trust, Department of Neonatal Medicine<br>Clark, Howard; University Hospital Southampton NHS Foundation Trust, Department of Neonatal Medicine; University of Southampton, Faculty of Medicine<br>Dimitrov, Borislav; University of Southampton, Primary Care and Population Sciences<br>Pope, Catherine; University of Southampton, Health Sciences<br>May, Carl; University of Southampton, Faculty of Health Sciences

**Primary Subject Heading**:	Health services research
Secondary Subject Heading:	Nutrition and metabolism, Paediatrics
Keywords:	Change management < HEALTH SERVICES ADMINISTRATION & MANAGEMENT, Protocols & guidelines < HEALTH SERVICES ADMINISTRATION & MANAGEMENT, Neonatal intensive & critical care < INTENSIVE & CRITICAL CARE, NEONATOLOGY, NUTRITION & DIETETICS

SCHOLARONE™
Manuscripts

Successful implementation and embedding of guidelines to improve the nutrition and growth of preterm infants in neonatal intensive care

Mark J Johnson[1,2](m.johnson@soton.ac.uk), Alison A Leaf[1,2](a.a.leaf@soton.ac.uk), Freya Pearson[2](freya.pearson@uhs.nhs.uk), Howard W. Clark[2,3](h.w.clark@soton.ac.uk) Borislav D. Dimitrov[4](b.dimitrov@soton.ac.uk), Catherine Pope[5] (c.j.pope@soton.ac.uk) & Carl R. May[5] (c.r.may@soton.ac.uk)

[1]National Institute for Health Research, Southampton Biomedical Research Centre, University Hospital Southampton NHS Foundation Trust and University of Southampton, Southampton, UK

[2]Department of Neonatal Medicine, Princess Anne Hospital, University Hospital Southampton NHS Foundation Trust, Southampton, UK

[3]University Child Health, Faculty of Medicine, University of Southampton, Southampton, Hampshire, UK

[4]Primary Care and Population Sciences, Faculty of Medicine, University of Southampton, Southampton, Hampshire, UK

[5]Faculty of Health Sciences, University of Southampton, Southampton, Hampshire, UK

Corresponding Author

Mark J. Johnson
Department of Neonatal Medicine
Mailpoint 105 Level E
University Hospital Southampton NHS Foundation Trust
Princess Anne Hospital
Coxford Road
Southampton
Hants
SO16 5YA
Tel: +442381204643
Email: m.johnson@soton.ac.uk

Keywords: Implementation, Premature Infant, Nutrition, Nutritional Assessment, Growth

Word Count: 4256

For peer review only

For peer review only - http://bmjopen.bmj.com/site/about/guidelines.xhtml

ABSTRACT

Objectives

We aimed to improve the nutritional care of preterm infants by developing a complex (multifaceted) intervention intended to translate current evidence into practice. We used the sociological framework of Normalization Process Theory (NPT), to guide implementation in order to embed the new practices into routine care,

Design

A prospective interventional study with a before and after methodology

Participants

Infants <30 weeks gestation or <1500g at birth.

Setting

Tertiary neonatal intensive care unit

Interventions

The intervention was introduced in phases: Phase 1 (Control period, Jan-Aug 2011); Phase 2 (Partial Implementation; improved parenteral and enteral nutrition solutions, nutrition team, education, Aug–Dec 2011); Phase 3 (Full implementation; guidelines, screening tool, 'nurse champions', Jan-Dec 2012); Phase 4 (Post implementation; Jan-Jun 2013). Bi-monthly audits and staff NPT questionnaires were used to measure guideline compliance and 'normalisation' respectively. NPT scores were used to guide implementation in real time. Data on nutrient intakes and growth were collected continuously.

Results

There were 52, 36, 75 and 35 infants in phases 1, 2, 3 and 4 respectively. Mean guideline compliance exceeded 75% throughout the intervention period, peaking at 85%. Guideline compliance and NPT scores both increased over time, (r=0.92 and 0.15, p<0.03 for both), with a significant linear association between the two (r=0.21, p<0.01). There were significant improvements in daily protein intake and weight gain between birth and discharge in phases 2 and 3 compared to phase 1 (p<0.01 for all), which were sustained into phase 4.

Conclusions

NPT and audit results suggest that the intervention was rapidly incorporated into practice, with high guideline compliance and accompanying improvements in protein intake and weight gain. NPT appears to offer an effective way of implementing new practices such that they lead to sustained changes in care. Complex interventions based on current evidence can improve both practice and clinical outcomes.

For peer review only - http://bmjopen.bmj.com/site/about/guidelines.xhtml

ARTICLE SUMMARY

Strengths and Limitations of the this study

- This study was novel in using a sociological theory (Normalisation Process Theory) to both guide and measure the process of implementation
- This study shows that complex interventions, when properly implemented, can change practice in a sustained fashion
- The before and after methodology used in this study is a limitation and means result should be interested with caution, but allowed the implementation process to be studied more closely and in 'real world' conditions.

For peer review only

For peer review only - http://bmjopen.bmj.com/site/about/guidelines.xhtml

MAIN MANUSCRIPT TEXT

BACKGROUND

Attempts to span translational gaps and implement evidence-based practice into routine clinical practice often fail [1, 2]. This can mean that patients fail to receive optimal treatment, or conversely may mean they receive unnecessary or potentially harmful care. Neonatal intensive care offers important opportunities for professional behaviour change and practice implementation but is a complex and demanding environment. The Neonatal Intensive Care Unit (NICU) has very vulnerable patients with complex and multiple medical problems, and a large multidisciplinary healthcare team working variable shift patterns. It is also a highly technological and information rich environment. Staff must manage and assimilate a constantly changing array of clinical information from a variety of sources, including monitoring equipment and computerised results systems. It is an interaction rich environment too: with complex interactions between different professionals, parents and patients themselves. It is a demanding environment to work in, with priorities constantly changing across the unit as new patients are admitted or others become clinically unstable.

The nutritional care and growth of preterm infants managed in the NICU is an important example of the problem of translating evidence into practice. Recommendations for nutrient intakes have been published [3, 4], however there is evidence that these are not effectively integrated into clinical practice [5]. There is also evidence that inconsistent and variable nutritional care may be partly responsible for sub-optimal growth. Neonatal units offering the same level of care have reported significant variations in rates of postnatal growth restriction and in length of stay, with differences in feeding practices shown to be one of the factors responsible for this variation [6]. Taking this together with the complexity of the NICU environment, it is understandable that current evidence and recommendations for practice fail to be consistently assimilated. We have recently discussed the issues surrounding context and complexity,

For peer review only - http://bmjopen.bmj.com/site/about/guidelines.xhtml

and it is clear that context has a profound effect on the extent to which new practices can be successfully implemented [7].

In this paper we describe the successful implementation of a nutrition guideline for preterm infants in a UK NICU leading to sustained change in practice. We show how integrating this guideline into patient care effectively required a carefully designed programme of translational work that facilitated both professional behaviour change (when professionals work differently) and practice implementation (when they embed new ways of conceptualizing, enacting and organizing practice into their workflow). We explain the operation of this programme of translational work using Normalization Process Theory (NPT) [8, 9], a conceptual tool-kit that helped us both to plan guideline implementation and to understand its dynamics [10]. More than 250 studies have now been reported that employ NPT. It offers a rigorous and transferable explanatory model of the mechanisms that promote implementation processes and fits well with the MRC Framework for Evaluating Complex Interventions [11, 12]. NPT has four main constructs; Coherence (whether people understand the need for change), Cognitive Participation (whether people understand the change itself and what they need to do to enact new practices), Collective action (whether people actually do the work needed for the new practices) and Reflexive monitoring (whether people see the benefit of the new practices in their daily work). In **Figure 1**, we show how the mechanisms that drive implementation processes are characterised in NPT. Whilst NPT provides a robust model of implementation that has often been used retrospectively to explain these process, it has less frequently been used to develop, guide and drive implementation prospectively as it was in the present study.

For peer review only

METHODS

For peer review only - http://bmjopen.bmj.com/site/about/guidelines.xhtml

Aims. We hypothesized that (i) the implementation of an evidence-based nutrition guideline for preterm infants would improve nutrient intakes and growth; and (ii) that the use of NPT to monitor and guide implementation of the guideline would result in its successful integration into practice. We anticipated that improvements in nutrient intake and growth that would follow from successful implementation would have important health benefits.

Setting and sample. The study was conducted in a NICU in the South of England. Inborn infants with a gestational age less than 30 weeks or birth weight less than 1501g were eligible for inclusion in the study, and were automatically included from birth to receive the newly implemented service for the provision and monitoring of nutrition for preterm infants. Staff were eligible for inclusion in the study if they were qualified clinicians (nurses, doctors, dietitians) rostered to NICU during the phase 2, 3 and 4 of the implementation study. They took part in individual structured (questionnaire) data collection using an online tool, and semi-structured (qualitative) interviews and focus groups facilitated by MJJ. The study was approved by an NHS Research Ethics Committee, ('Oxford 'B'' Reference 11/sc/0365). **Figure 2** shows a flow chart of the study.

Intervention development. A complex intervention was developed with the aim of translating evidence about the nutritional care of preterm infants into practice. It was based on current literature and practice recommendations available at the time (see additional file 1). To improve the likelihood of implementation and embedding in practice, each component of the intervention also aimed to target implementation mechanisms identified by NPT[13]. The implementation intervention had seven major components:

- A comprehensive nutrition guideline (see additional file 1).
- A screening tool to identify nutritional risk, linked to specific guideline pathways and nutrition review [14].

For peer review only - http://bmjopen.bmj.com/site/about/guidelines.xhtml

- Improved nutritional products: Stock PN solutions were revised to provide more nutrition in a smaller volume and new formula milks and breast milk fortifier introduced with higher nutritional content.
- A multidisciplinary nutrition support team, (consultant neonatologist with an interest in nutrition, a neonatal dietitian, a neonatal pharmacist and nurse champions).
- Nurse champions seconded one day in five to the nutrition team to improve their knowledge and skills nutritional care, and four days in five working clinically, supporting their colleagues in the new ways of working[15].
- A weekly nutrition ward round to review infants at the highest nutritional risk and provide additional management plans for nutrition

Once developed, the clinical guidelines were circulated to staff and two focus groups held in order to both raise awareness of the changes in practice and to gain insight into potential barriers or facilitators to the implementation process, enabling tailoring of the guidelines to the local setting.

Guideline implementation. This was an observational study. Data were collected in discrete periods between January 2011 and June 2013:

a. ***Control period*** (1st January 2011 and 31st July 2011). Nutrient intake and growth data on infants born during this period were collected retrospectively after the study had finished in order to provide a contemporaneous ‘control’ group.
b. ***Intervention planning and introduction of improved nutrition products*** (August 1st – December 31st 2011). Nutrient intake and growth data on infants were collected prospectively during this period, during which some elements of the intervention (including improved nutritional solutions) were introduced, and staff were consulted about guideline intervention and its associated changes

For peer review only - http://bmjopen.bmj.com/site/about/guidelines.xhtml

in organization and practice. In addition, the work with staff carried out during this period to develop the intervention would also be likely to begin to affect practice.

c. ***Facilitated guideline implementation*** (January 1st- December 31st 2012) during which the full complex intervention was implemented. Nutrient intake and growth data on infants were collected prospectively and audits of guideline compliance and staff NPT Toolkit questionnaires were carried out bi-monthly.

d. ***Post-implementation phase*** (January 1st- June 30th 2013). Nutrient intake and growth data on infants were collected prospectively during this period, and one final audit of guideline compliance was carried out to assess the degree to which the new practices remained in place after the main intervention period.

Patient outcomes. Infant outcomes of primary interest were (i) differences in mean daily energy and protein intakes during stay on NICU between pre-implementation and intervention periods, and (ii) differences in the change in weight and head circumference standard deviation scores (SDS) between birth and discharge. These data were collected by entering infant chart data on fluid and feed intake into a specially designed spreadsheet, which was pre-programmed with the nutrient content of feeds and fluids available on the NICU, and automatically calculated daily energy and protein intakes for each infant. Growth data were collected in a similar manner and converted to SDS using the LMS growth add in for Microsoft Excel using reference data from the UK-WHO Newborn Infant Close Monitoring growth chart. Differences in patient outcomes were also detected by monitoring routinely collected data on mortality, morbidity (e.g. necrotising enterocolitis; chronic lung disease; retinopathy of prematurity; severe Intraventricular haemorrhage; late onset sepsis) and length of stay.

Guideline normalization and compliance. Measures of nutritional processes were extracted from patient charts at the time of nutritional data entry: time of starting enteral feeds, time of starting PN,

For peer review only

For peer review only - http://bmjopen.bmj.com/site/about/guidelines.xhtml

time of starting breast milk fortifier and type of feed at discharge. Audits of compliance with the nutrition guideline were carried out throughout the full implementation period, and again at the end of the post-implementation period [16]. Audits were carried out every two months in the implementation phase, and once in the post-implementation phase. Measures of the normalization of guideline compliance were made using a questionnaire based on the NPT online toolkit (www.normalizationprocess.org). This was adapted to ensure that questions related to implementing and embedding the nutrition guideline in practice. This was made available to staff online using www.freesurveys.com. Respondents were asked to score their level of agreement with each of the 16 items between one and ten. This provided overall scores for each of the four domains of NPT (sense-making, participation, action and monitoring). Staff completed questionnaires anonymously.

Statistical analysis. Descriptive statistics was used to summarise the demographic and outcome variables. The outcome variables were tested for normality using the Kolmogorov–Smirnov test in order to help determine the nature of the analysis methods used, with $p<0.05$ indicating that the tested variable distribution differed from a normal distribution. For normally distributed continuous variables, the mean and standard deviation were calculated, with the median and interquartile range calculated for other continuous variables. Distribution of categorical variables was presented as frequency and percentage. Comparison of daily nutrient intake and growth data between periods was carried out using general linear models with mixed effects. This statistical technique accounts for repeated measures in the same infant, allowing the addition of other potentially confounding variables (sex, gestational age at birth and birth weight) and subsequent adjustment of the model. Post-hoc Tukey's test was used to adjust significance values in view of multiple comparisons. For normally distributed data, a type of general linear model was used, whilst for non-normally distributed data a type of generalized linear model was used in which repeated effects are considered random effects.

For peer review only - http://bmjopen.bmj.com/site/about/guidelines.xhtml

Mortality and morbidity data and other dichotomous outcomes were compared across study periods using X^2 tests (or Fishers Exact test where numbers were low). Continuous process outcome measures were compared across study periods using either a two-way ANOVA (for normally distributed data) or the Kruskal-Wallis test (for non-normally distributed data). If significant differences were found then comparisons between pairs of groups were further analysed with post hoc adjustment by Tukey's test (normally distributed data) or multiple Mann-Whitney-U tests (non-normally distributed data).

Guideline compliance audit results and measures of the 'normalisation' of practice (using scores from the online NPT questionnaire) were summarised as mean scores and plotted over time. Multiple linear regression was used to describe the nature of the relationship between mean percentage audit compliance and NPT scores over time. A similar approach was then used to relate mean percentage audit compliance and NPT scores to the primary infant outcome measures. Plots of mean percentage audit compliance and NPT scores were overlaid with plots of energy intakes, protein intakes and the differences in weight and head circumference SDS between birth and discharge over time during the intervention period. The analyses were carried out using Stata IC v12.3 (Stata Corp) and SAS 9.3 (SAS Institute Inc.).

RESULTS

Measures of Infant Outcomes. **Table 1** summarises the sex, gestational age at birth and birth weight of infants in each study period. CRIB II[17] scores are also shown as in indication of illness severity. CRIB II scores were not available for all infants and the numbers available with CRIB scores are also shown in **Table 2**. There were no significant differences in sex, birth weight or gestational age between groups. There was a significant difference in CRIB II scores between groups (p=0.008), with post hoc pairwise

For peer review only - http://bmjopen.bmj.com/site/about/guidelines.xhtml

testing using Tukey's method revealing that only group D was significantly different (higher) from all the others. This suggests an increased level of illness severity in group D when interpreting results.

Period	n	Male (%)	Mean Birth weight (SD)	Mean Gestational Age (SD)	Mean CRIB II (SD), n
A. Pre-implementation period (Jan 2011 – Jul 2011	52	23 (44.2)	1.084 (0.270)	29.2 (2.6)	7.0 (3.6), 30
B. Partial implementation period (Aug – Dec 2011)	36	18 (50)	1.029 (0.311)	29.2 (2.9)	6.4 (3.9), 20
C. Main Intervention Period (Jan – Dec 2012)	75	37 (49.3)	0.998 (0.269)	28.7 (3.0)	6.9 (2.5), 44
D. Post-implementation period (Jan – Jun 2013)	35	22 (62.9)	0.924 (0.261)	28.1 (2.8)	9.7 (3.2), 18
p value for difference between groups (ANOVA)		0.392*	0.066	0.290	**0.008**

Table 1: Infant Characteristics in each study group (SD-Standard Deviation) *p value is for Chi^2

Outcome	Mean nutritional process audit compliance	
	Model with Time Excluded	Model with Time Included
**Mean NPT Score Coefficient (p value)**	0.95 (0.002)	0.40 (0.031)
**Time coefficient (p value)**	Omitted	0.72 (<0.0001)
**p value for model**	0.0018	<0.0001
**r for model**	0.2098	0.8076
**r^2 for model**	0.044	0.6522

Table 2: Results of linear regression for mean audit compliance measures and mean NPT scores over time.

Nutrient Intakes over time. When compared with baseline data, progressive increases in protein intake were observed over the course of the study. **Figures 3a-d** show the results of the generalised linear

For peer review only

For peer review only - http://bmjopen.bmj.com/site/about/guidelines.xhtml

modelling analysis for median daily nutrient intakes for each of energy (kcal/kg/day), protein (g/kg/day), energy (as a percentage of RRI) and protein (as a percentage of RRI) respectively. Using Tukey's test to compare the difference between each period, there were significant improvements in protein intake in period B and C compared to period A (both p<0.001), and this was sustained beyond the intervention into period D (p<0.01 vs periods A and B). In particular, there was a significant increase protein intake between the intervention planning phase (B) and the post implementation phase (D).

Growth over time The results of the general linear model using mixed effects for the changes in weight and head circumference SDS in each study period are shown in **Figure 4**. Using Tukey's test to compare the difference between each period, there was a significant and sequential improvement in the change in standard deviation score from birth (cSDS) for weight in period B and C compared to period A (both p<0.01), which again were sustained post implementation in period D (p<0.001 vs periods A and B). This demonstrates that there was a sequential improvement in the difference in weight SDS between birth and discharge in each study period during the study. There was a non-significant improvement in the cSDS for head circumference (HC) across the study.

Mortality and Morbidity. No significant differences were detected in the rates of mortality, chronic lung disease, necrotising enterocolitis, severe intraventricular haemorrhage, retinopathy of prematurity and late onset infection.

Professional behaviour change and practice implementation

Timing of commencement of feeds and types of feed. There were no significant differences in the number of babies receiving breast milk, preterm formula, term formula or mixed feeding at discharge between phases of the study. There were no significant changes in the proportion of breast milk fed

For peer review only - http://bmjopen.bmj.com/site/about/guidelines.xhtml

infants receiving fortifier, nor were their differences in the time to start enteral feeds or the time of starting fortifier in infants receiving breast milk between study periods. However, there were differences in the median time to starting parenteral nutrition between the phases of the study. In the baseline or control phase of the study this was 15 hrs. Over the pre-implementation and implementation phases of the study this reduced to nine hours. In the post implementation phase this rose to 12 hours. A significant difference between study phases was detected using the Kruskal-Wallis test (p=0.013).

Adherence to Guideline. Bimonthly guideline compliance audits – described in Figure 2 – during the intervention phase and at the end of the post-implementation phase showed that mean compliance improved incrementally across the implementation phase, but there was a slight decrease in compliance at the final audit in July 2013. Linear regression of mean nutritional audit compliance during the 12 months of the intervention period demonstrated a significant linear increase over time, with a regression coefficient of 1.1 (r=0.92, p=0.009).

Normalisation Process Theory Scores. Taking into account participant dropout due to staff turnover, response rates to the NPT Toolkit questionnaire peaked at 74% in May 2012, falling to 27% in the final questionnaire in July 2013. Details regarding the number and type of respondents can be seen in **table 3**. **Figure 5** shows NPT scores as radar plots for each time period ; in general, the fuller the radar plot, the greater extent to which staff felt that the practices were part of 'normal practice' at that time. Radar plots generally become fuller over time, though some key areas of the plots were less full at different time points, indicating areas for improvement. The items relating to collective action and reflexive monitoring were scoring lower early in the intervention period, indicating that staff could not see the benefit of the intervention in their work. In order to address this, the results of the study to date were displayed around the staff areas of NICU in August 2012, with a subsequent improvement in the related NPT scores. There was a significant linear increase in mean NPT score over time (coefficient=0.031,

For peer review only

r=0.15, p=0.023), though this dropped off during the post-implementation phase. **Figure 6** shows that global NPT scores and guideline compliance increased together over time and then flattened out in the post implementation phase. Linear regression analysis showed that there was a significant association between mean global NPT scores and audit compliance through the intervention development, implementation and post-implementation phases of the study with a coefficient of 0.95 (r=0.21, p=0.002, see table 2). The addition of time as a variable into the linear regression models (to account for the repeated measures nature of the data) is also shown in table 2. The addition of time significantly contributed to the increases in compliance over the study and increased the predictive value of the model, though despite this the mean NPT scores remained a significant predictor, showing that the measures of normalisation using NPT are associated with measures of clinical practice. Linear regression using the mean individual construct scores for NPT showed a significant association with the mean audit scores and participants' capacity to monitor the effects of their actions (*reflexive monitoring*), both before and after adjustment for the effect of time (coefficients of 0.89 and 0.51, p=0.034 and p=0.044 with and without adjustment for time respectively).

Time Period	Mar-12	May-12	Jul-12	Sep-12	Nov-12	Jan-13	Jul-13
Number of Respondents	44	52	39	26	24	18	16
Percentage Response Rate	57.9	74.3	58.2	41.3	40.7	31	27
Number (%) Consultants	4 (9.1)	4 (7.7)	4 (10.3)	4 (15.4)	4 (16.7)	3 (16.7)	4 (25)
Number (%) Junior Doctors/ANNPs	1 (2.3)	3 (5.8)	3 (7.7)	0 (0)	0 (0)	0 (0)	0 (0)
Number (%) Pharmacists	1 (2.3)	1 (1.9)	1 (2.6)	0 (0)	0 (0)	0 (0)	0 (0)
Number (%) Band 7 Nurses	4 (9.1)	4 (7.7)	2 (5.1)	3 (11.5)	5 (20.8)	2 (11.1)	2 (12.5)
Number (%) Band 6 Nurses	10 (22.7)	9 (17.3)	6 (15.4)	7 (26.9)	6 (25.0)	5 (27.8)	4 (25.0)
Number (%) Band 5 Nurses	19 (43.1)	23 (44.2)	18 (46.2)	10 (38.5)	6 (25.0)	5 (27.8)	4 (25)
Number (%) Band 4 Nurses	2 (4.6)	4 (7.7)	2 (5.1)	1 (3.9)	1 (4.2)	0 (0)	2 (12.5)

For peer review only - http://bmjopen.bmj.com/site/about/guidelines.xhtml

Number (%) Band 3 Nurses or lower	3 (6.8)	4 (7.7)	3 (7.7)	1 (3.85)	2 (8.3)	1 (5.6)	1 (6.3)

Table 3: Number of respondents and percentage response rate for each NPT questionnaire

DISCUSSION

We evaluated the effects of guideline implementation by measuring objective changes in nutrition intake. These data are important in their own right, but can also be used to corroborate subjective self-reports of behaviour change and practice implementation by staff. Objective improvements in nutrient intake and weight gain were detected in infants across the four data collection periods. Against this background, mean audit guideline compliance and NPT scores both increased in a linear fashion over time. Impressively, mean guideline compliance was in excess of 75% throughout the intervention period, peaking at 85%. The headline result of this study is that implementation of the guideline was successfully achieved, and that activities associated with specific intervention components were routinely embedded in workflow within the NICU.

This paper has described the successful implementation of a nutrition guideline for preterm infants in NICU, leading to sustained change in practice and improved nutritional outcomes. During the time this study was active, other groups have used similar approaches in the preterm population in order to try and improve infant growth in NICU [18, 19]. They also used before and after study designs, but did not include a process evaluation. Our study has shown that implementing a facilitated nutrition guideline in NICU using a multifaceted intervention improved protein intake and weight gain in preterm infants. Our process evaluation demonstrates that using NPT to develop and guide the implementation process can lead to high compliance with guidelines and changes in practice that are sustained beyond the initial intervention period. The results also show that measures of normalisation using the NPT toolkit correlate well with measures of clinical practice in real life, and suggest that NPT may therefore offer an

For peer review only

For peer review only - http://bmjopen.bmj.com/site/about/guidelines.xhtml

effective way of measuring and guiding the implementation process. Effectively implementing the components of this intervention significantly improved both protein intake and weight gain, and appeared to prevent the 'expected' fall of around 1.5-2 SDS for weight between birth and discharge reported in other studies [20, 21]. This may be clinically relevant; for example, it may lead to improved neurodevelopmental outcomes [22-24]. Improvement in weight gain and protein intake appears to continue into the post implementation period, suggesting that improvements were sustained beyond the main intervention period.

In the present study, audits of guideline compliance were used in combination with the NPT toolkit. The audits measured how well the guideline was put into practice, and the toolkit provided insight into how well the intervention was being integrated into routine care by staff and identified areas where more work was needed to aid implementation. NPT was used prospectively for the first time in this study to develop and drive the intervention, rather than retrospectively assessing the implementation process. In particular, the guidelines were aimed at encouraging *coherence* and *cognitive participation* by being clear about the reasoning behind the approaches used and how to use them. Similarly, the nutrition team, nurse champions and nutrition ward round aimed to provide feedback to aid *reflexive monitoring*. Audit compliance generally improved over the course of the intervention period, and was around 80%, which is exceptionally high for studies of implementation. NPT scores generally increased over time, suggesting the intervention was becoming 'normalised' into practice. While the use of the NPT Toolkit to measure normalisation in this study was novel and experimental, it seems that the measure of 'normalisation' provided by the NPT toolkit does relate to practice changes in the 'real world'. Here, subjective self-reports by staff related well to objective measures of guideline compliance. Global NPT scores were high even at the start of the intervention, suggesting that staff felt the intervention became embedded into routine care rapidly

For peer review only - http://bmjopen.bmj.com/site/about/guidelines.xhtml

A notable result of this study is the importance of reflexive monitoring of implementation progress by staff. This was significantly associated with audit compliance (r = 0.25). However, it accounted for 6% of the variation in audit compliance and it had an effect size of an improvement of 0.9% audit compliance for every point in global NPT scores. Seeing the impact of personal action functions as a feedback mechanism, and such 'feedback loops' are likely to be responsible for the efficacy of professional interventions such as 'audit and feedback' and 'educational outreach' from other health professionals [10]. Both of these were central components of the intervention. These findings are also consistent with those of a previous theory-led overview of systematic reviews of professional interventions using NPT by our group, which showed that those interventions that emphasised *reflexive monitoring* were more likely to be successful [10]. Showing staff the results of the study to date during the main implementation period in response to low *reflexive monitoring* scores demonstrates the utility of NPT to identify issues and make implementation a more dynamic process. It also illustrates how addressing such issues results in responsive changes that can be seen in subsequent NPT scores, suggesting that NPT offers a way to both *measure* and *guide* change.

We have previously discussed the importance of context in relation to implementation, suggesting that NPT is also able to provide a lens through which to consider the interactions between context and complex interventions [7]. We proposed that the *plasticity* of interventions and the *elasticity* of the context into which they were introduced played a significant part in the degree of implementation success. Using NPT in the present study to both develop and guide the implementation process, perhaps helped overcome the issues with the complex context of the NICU, providing contemporaneous feedback on the barriers to implementation and allowing a degree of plasticity of the intervention itself. This process was also facilitated by the focus groups prior to implementation, allowing potential barriers to be overcome by alterations in the intervention components and the way in which they were

For peer review only - http://bmjopen.bmj.com/site/about/guidelines.xhtml

delivered. In addition, the focus groups suggested a desire from staff for more consistency in nutritional care, and this in turn is likely to have improved the elasticity of the host context, facilitating *normative restructuring* around the intervention and aiding implementation. This may explain the high degree of compliance and normalisation seen in the present study.

There were some limitations to this study. As a controlled before and after study, it is not possible to be sure if any of the changes seen during the study are a direct result of the intervention. As this was not a randomised controlled trial, it cannot control for causal mechanisms and confounders, and as such it is subject to limits of interpretation. Whilst the statistical analyses show associations between the progressive implementation of the intervention and changes in outcomes, it cannot prove causation. A further limitation relates to having adequate patient numbers and statistical power to detect important differences, which may possibly account for the failure to detect a clinically significant improvement in head circumference. The study was also not powered to detect differences in mortality and morbidity data. An important limitation of the NPT toolkit questionnaires used in this study is that staff responses may have been biased by their beliefs about the expectations of the study team, which is a common problem in such studies.

CONCLUSION

This study used nutrition in the NICU as a vehicle to understand implementation in a complex environment. It has demonstrated that the implementation of the facilitated guideline was associated with improvements in infant protein intakes and weight. The use of NPT to guide and monitor the implementation of the intervention resulted in high guideline compliance and a degree of 'normalisation' of the complex intervention into routine care. Measures of normalisation using NPT

For peer review only - http://bmjopen.bmj.com/site/about/guidelines.xhtml

appear to relate to objective measures of practice, suggesting that NPT could provide a useful way of understanding the dynamics of implementation processes in complex clinical environments.

For peer review only

For peer review only - http://bmjopen.bmj.com/site/about/guidelines.xhtml

LIST OF ABBREVIATIONS:

ALP – Alkaline Phosphatase; ESPGHAN - European Society for Pediatric Gastroenterology; Hepatology and Nutrition; NICU - Neonatal Intensive Care Unit; NPT – Normalization Process Theory; PN – Parenteral Nutrition; RNI – Reference Nutrient Intake; RRI – Reasonable Range of Intake; WHO – World Health Organisation.

DECLARATIONS

Ethics approval and consent to participate

The study was approved by an NHS Research Ethics Committee, ('Oxford 'B'' Reference 11/sc/0365)

Consent for publication

Not applicable

Availability of data and material

The datasets generated and/or analysed during the current study are not publicly available due to further pending publications and current approvals, but may be available from the corresponding author on reasonable request. An implementation toolkit and a validated instrument to measure implementation processes using Normalisation Process Theory are available at www.normalizationprocess.org.

Competing interests

For peer review only - http://bmjopen.bmj.com/site/about/guidelines.xhtml

All authors have completed the ICMJE uniform disclosure form at www.icmje.org/coi_disclosure.pdf and declare: no support from any organisation for the submitted work; CRM is an original author of Normalization Process Theory; no other relationships or activities that could appear to have influenced the submitted work.

Funding

Work leading to this paper was funded by the National Institute for Health Research (NIHR) Biomedical Research Centre, Southampton. MJ's contribution was partly supported by an NIHR Doctoral Research Fellowship DRF-2012-05-272, and CRM's contribution was partly supported by NIHR CLAHRC Wessex and partly by ESRC Grant ES-062-23-3274. We gratefully acknowledge the financial support from these agencies. Funders had no role in the design of the study and collection, analysis and interpretation of the data or in writing the manuscript, and the paper does not necessarily represent their views.

Authors' contributions

MJJ contributed to the design of the study, carried out data analysis and interpreted all data. He was responsible for drafting the article and revising it critically for important intellectual content. He is guarantor. AAL, FP, HWC contributed to the conception and design of the study and interpretation of data. They revised the article critically for important intellectual content. BDD supervised the statistical analysis and developed the statistical model used for longitudinal data analysis. He contributed to the interpretation of data and revised the article critically for important intellectual content. CJP and CRM contributed to the design of the study, the use of NPT in the study and interpretation of data. They revised the article critically for important intellectual content.

Acknowledgements

For peer review only - http://bmjopen.bmj.com/site/about/guidelines.xhtml

We would like to further acknowledge the work of Amanda Beedham and Karen Hayllar at Co.Efficient Consultancy, who provided expertise in the development phase and were contracted to develop and build the electronic tool for calculating and storing daily nutrient intake and growth data. We would also like to acknowledge Miss Zoe Lansdowne and Dr Amanda Bevan (Neonatal and Paediatric Pharmacists, University Hospital Southampton NHS Foundation Trust) for their help with the parenteral nutrition solution data used in the electronic tool.

We would also like to acknowledge Jenny Pond, Jane Rhodes-Kitson, Charlotte Oates, Jenny Weddell and, Linda Anderson, Christina Humphrey and Liz Blake for their help in data collection and in their roles as nurse 'Champions for Nutrition'.

FIGURE TITLES AND LEGENDS

Figure 1: The Model of Normalization Process Theory
The four main constructs of NPT are shown in bold. Reproduced with permission [8]

Figure 2: Study process flow chart

Figure 3: Bar graphs showing mean daily nutrient intakes across the four study periods
Bars show mean daily Energy in kcal/kg/day (A), protein in g/kg/day (B), energy as a percentage of RRI (C) and protein as a percentage of RRI (D).Error bars represent 95% confidence intervals. Blue bars represent unadjusted data, while red bars are adjusted for sex, gestational age and weight at birth. *p<0.05 for difference vs period A, †p<0.05 for difference vs period B, +p<0.05 for difference vs period C. (RRI- reasonable range of intake)

Figure 4: Bar graphs showing mean change in standard deviation score (SDS) across the four study periods
Changes are from birth until discharge for weight (A) and head circumference (B). Error bars represent 95% confidence intervals. Blue bars represent unadjusted data, whilst red bars are adjusted for sex, gestational age and weight at birth *p<0.05 for difference vs period A, †p<0.05 for difference vs period B, +p<0.05 for difference vs period C

Figure 5: Radar plots showing the mean score for each sub-construct of NPT
Results from the NPT questionnaire taken throughout the course of the study.

Figure 6: Relationship over time between mean NPT scores and percentage guideline compliance

For peer review only - http://bmjopen.bmj.com/site/about/guidelines.xhtml

ADDITIONAL FILES

Additional File 1.pdf: Nutrition guideline used in this study

For peer review only

For peer review only - http://bmjopen.bmj.com/site/about/guidelines.xhtml

REFERENCES

1. Grimshaw JM, Eccles MP, Lavis JN, et al. Knowledge translation of research findings. Implement Sci 2012;**7**:50 doi: 10.1186/1748-5908-7-50

1748-5908-7-50 [pii][published Online First: Epub Date]|.

2. Eccles M, Mittman B. Welcome to Implementation Science. Implementation Science 2006;**1**(1):1 doi: 10.1186/1748-5908-1-1[published Online First: Epub Date]|.

3. Tsang RC. *Nutrition of the preterm infant*. 2nd ed. ed. Cincinnati: Digital Educational Publishing, 2005.

4. Agostoni C, Buonocore G, Carnielli VP, et al. Enteral nutrient supply for preterm infants: commentary from the European Society of Paediatric Gastroenterology, Hepatology and Nutrition Committee on Nutrition. J Pediatr Gastroenterol Nutr 2010;**50**(1):85-91 doi: 10.1097/MPG.0b013e3181adaee0[published Online First: Epub Date]|.

5. Embleton NE, Pang N, Cooke RJ. Postnatal malnutrition and growth retardation: an inevitable consequence of current recommendations in preterm infants? Pediatrics 2001;**107**(2):270-3

6. Cooke RJ, Ainsworth SB, Fenton AC. Postnatal growth retardation: a universal problem in preterm infants. Arch Dis Child Fetal Neonatal Ed 2004;**89**(5):F428-30 doi: 10.1136/adc.2001.004044

89/5/F428 [pii][published Online First: Epub Date]|.

7. May CR, Johnson M, Finch T. Implementation, context and complexity. Implement Sci 2016;**11**(1):141 doi: 10.1186/s13012-016-0506-3

10.1186/s13012-016-0506-3 [pii][published Online First: Epub Date]|.

8. May C, Finch T. Implementing, Embedding, and Integrating Practices: An Outline of Normalization Process Theory. Sociology 2009;**43**(3):535-54 doi: 10.1177/0038038509103208[published Online First: Epub Date]|.

9. May CR, Mair F, Finch T, et al. Development of a theory of implementation and integration: Normalization Process Theory. Implementation Science 2009;**4**(1):29 doi: 10.1186/1748-5908-4-29[published Online First: Epub Date]|.

10. Johnson MJ, May CR. Promoting professional behaviour change in healthcare: what interventions work, and why? A theory-led overview of systematic reviews. BMJ Open 2015;**5**(9):e008592 doi: 10.1136/bmjopen-2015-008592

bmjopen-2015-008592 [pii][published Online First: Epub Date]|.

11. Craig P, Dieppe P, Macintyre S, et al. Developing and Evaluating Complex Interventions: New Guidance: Medical Research Council, 2008.

12. Tabak RG, Khoong EC, Chambers DA, et al. Bridging research and practice: models for dissemination and implementation research. Am J Prev Med 2012;**43**(3):337-50 doi: 10.1016/j.amepre.2012.05.024

S0749-3797(12)00389-3 [pii][published Online First: Epub Date]|.

13. Murray E, Treweek S, Pope C, et al. Normalisation process theory: a framework for developing, evaluating and implementing complex interventions. BMC medicine 2010;**8**:63 doi: 10.1186/1741-7015-8-63[published Online First: Epub Date]|.

14. Johnson MJ, Pearson F, Emm A, et al. Developing a new screening tool for nutritional risk in neonatal intensive care. Acta Paediatr 2015;**104**(2):e90-3 doi: 10.1111/apa.12855[published Online First: Epub Date]|.

For peer review only - http://bmjopen.bmj.com/site/about/guidelines.xhtml

15. Westbury JA, Johnson MJ, Pond JP, et al. Developing the role of the nurse as a link advisor for research and a champion for nutrition in the neonatal intensive care unit. Journal of Neonatal Nursing 2013;**19**:198 doi: http://dx.doi.org/10.1016/j.jnn.2013.01.003[published Online First: Epub Date]|.

16. Lee L, Girish S, van den Berg E, et al. Random safety audits in the neonatal unit. Arch Dis Child Fetal Neonatal Ed 2009;**94**(2):F116-9 doi: adc.2007.131052 [pii]

10.1136/adc.2007.131052[published Online First: Epub Date]|.

17. Parry G, Tucker J, Tarnow-Mordi W. CRIB II: an update of the clinical risk index for babies score. Lancet 2003;**361**(9371):1789-91 doi: S0140-6736(03)13397-1 [pii]

10.1016/S0140-6736(03)13397-1[published Online First: Epub Date]|.

18. Rochow N, Fusch G, Muhlinghaus A, et al. A nutritional program to improve outcome of very low birth weight infants. Clin Nutr 2012;**31**(1):124-31 doi: 10.1016/j.clnu.2011.07.004

S0261-5614(11)00131-2 [pii][published Online First: Epub Date]|.

19. Roggero P, Gianni ML, Orsi A, et al. Implementation of nutritional strategies decreases postnatal growth restriction in preterm infants. PLoS One 2012;**7**(12):e51166 doi: 10.1371/journal.pone.0051166

PONE-D-12-22771 [pii][published Online First: Epub Date]|.

20. Ehrenkranz RA, Younes N, Lemons JA, et al. Longitudinal growth of hospitalized very low birth weight infants. Pediatrics 1999;**104**(2 Pt 1):280-9

21. Cole TJ, Statnikov Y, Santhakumaran S, et al. Birth weight and longitudinal growth in infants born below 32 weeks' gestation: a UK population study. Arch Dis Child Fetal Neonatal Ed 2013 doi: 10.1136/archdischild-2012-303536

archdischild-2012-303536 [pii][published Online First: Epub Date]|.

22. Ehrenkranz RA, Dusick AM, Vohr BR, et al. Growth in the neonatal intensive care unit influences neurodevelopmental and growth outcomes of extremely low birth weight infants. Pediatrics 2006;**117**(4):1253-61 doi: 117/4/1253 [pii]

10.1542/peds.2005-1368[published Online First: Epub Date]|.

23. Stephens BE, Walden RV, Gargus RA, et al. First-week protein and energy intakes are associated with 18-month developmental outcomes in extremely low birth weight infants. Pediatrics 2009;**123**(5):1337-43 doi: 123/5/1337 [pii]

10.1542/peds.2008-0211[published Online First: Epub Date]|.

24. Chan SH, Johnson MJ, Leaf AA, et al. Nutrition and neurodevelopmental outcomes in preterm infants: a systematic review. Acta Paediatr 2016;**105**(6):587-99 doi: 10.1111/apa.13344[published Online First: Epub Date]|.

For peer review only - http://bmjopen.bmj.com/site/about/guidelines.xhtml

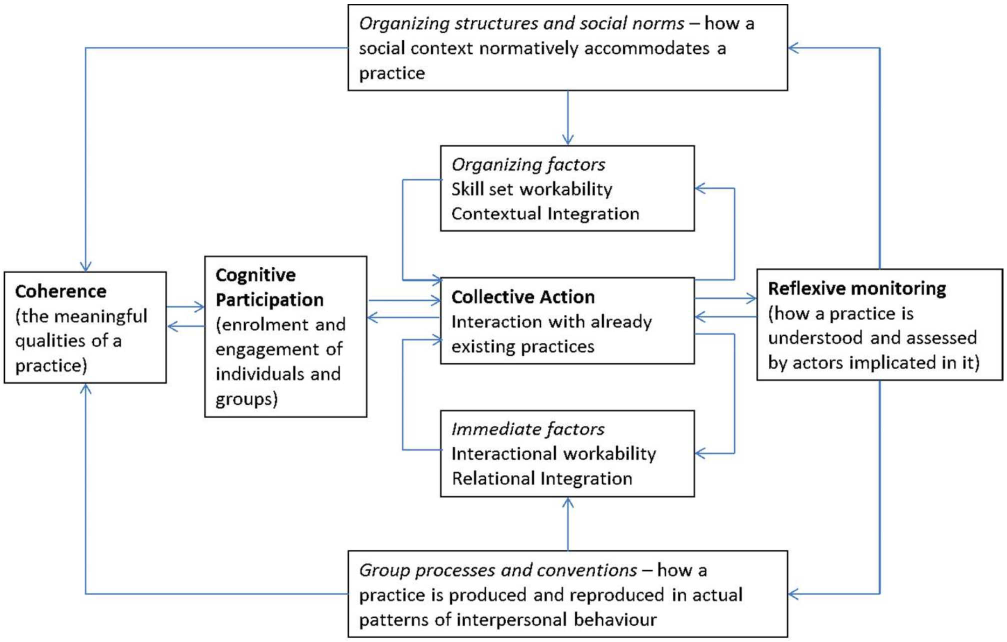

Figure 1: The Model of Normalization Process Theory
The four main constructs of NPT are shown in bold. Reproduced with permission [8]

374x239mm (96 x 96 DPI)

For peer review only

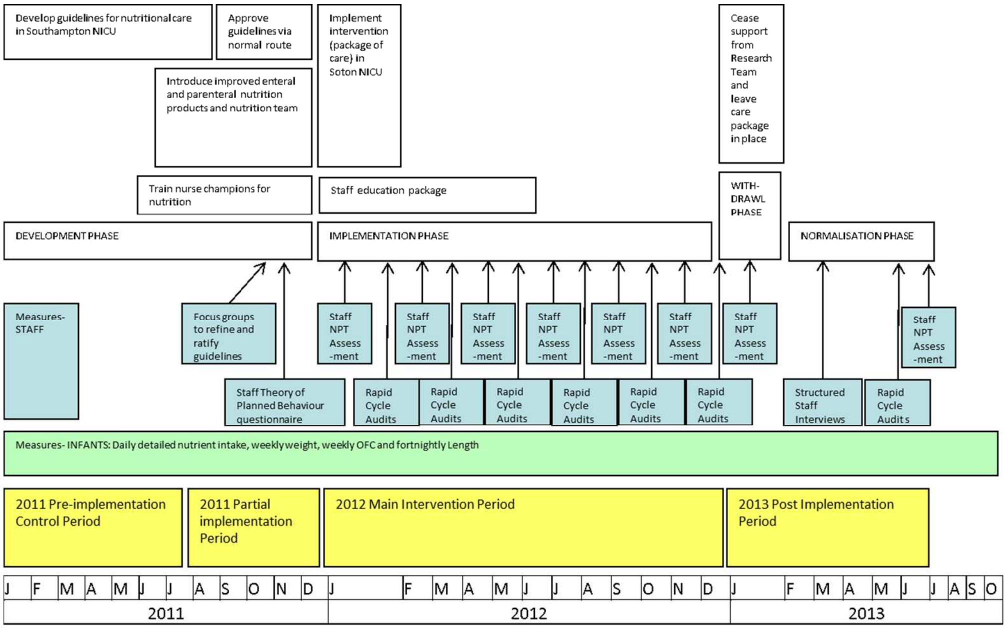

Figure 2: Study process flow chart

345x217mm (96 x 96 DPI)

view only

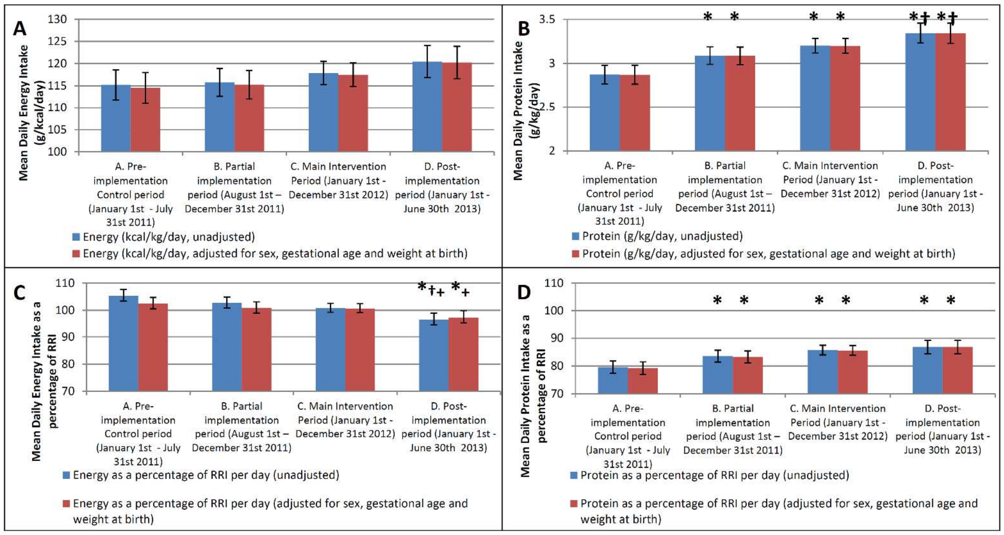

Figure 3: Bar graphs showing mean daily nutrient intakes across the four study periods
Bars show mean daily Energy in kcal/kg/day (A), protein in g/kg/day (B), energy as a percentage of RRI (C) and protein as a percentage of RRI (D).Error bars represent 95% confidence intervals. Blue bars represent unadjusted data, while red bars are adjusted for sex, gestational age and weight at birth. *p<0.05 for difference vs period A, †p<0.05 for difference vs period B, +p<0.05 for difference vs period C. (RRI- reasonable range of intake)

1157x613mm (96 x 96 DPI)

view only

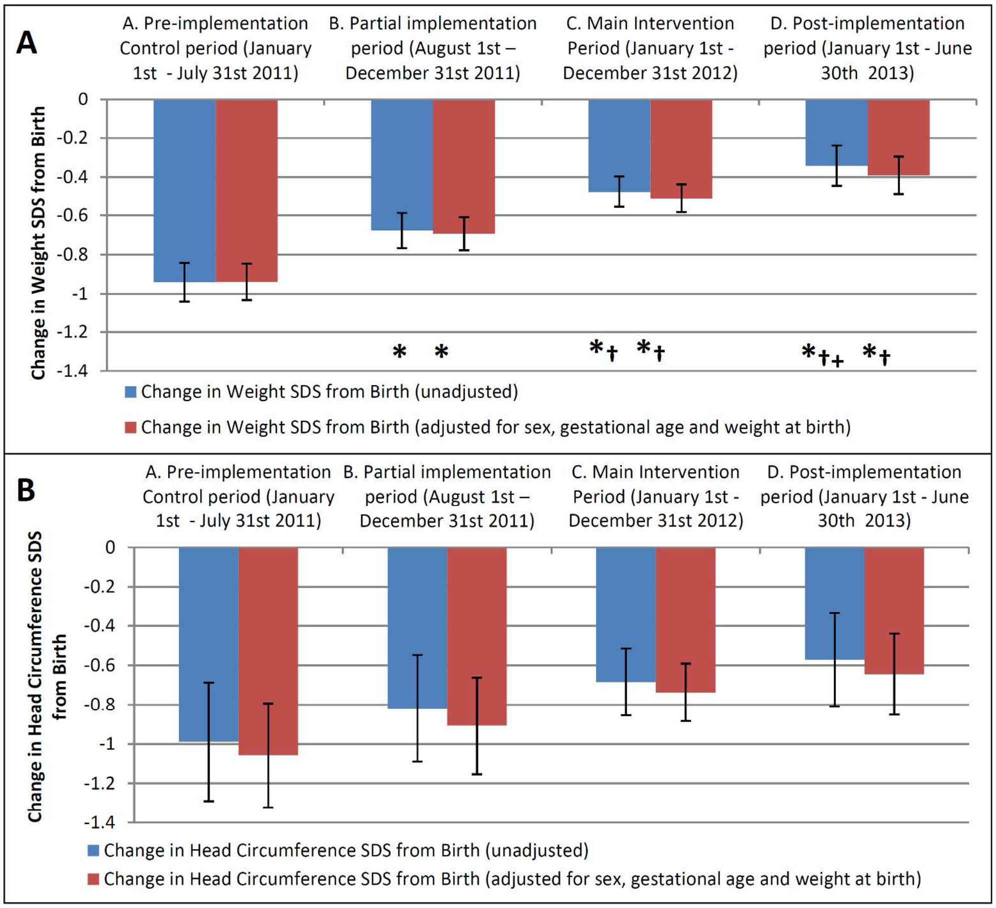

Figure 4: Bar graphs showing mean change in standard deviation score (SDS) across the four study periods Changes are from birth until discharge for weight (A) and head circumference (B). Error bars represent 95% confidence intervals. Blue bars represent unadjusted data, whilst red bars are adjusted for sex, gestational age and weight at birth *p<0.05 for difference vs period A, †p<0.05 for difference vs period B, +p<0.05 for difference vs period C

740x673mm (96 x 96 DPI)

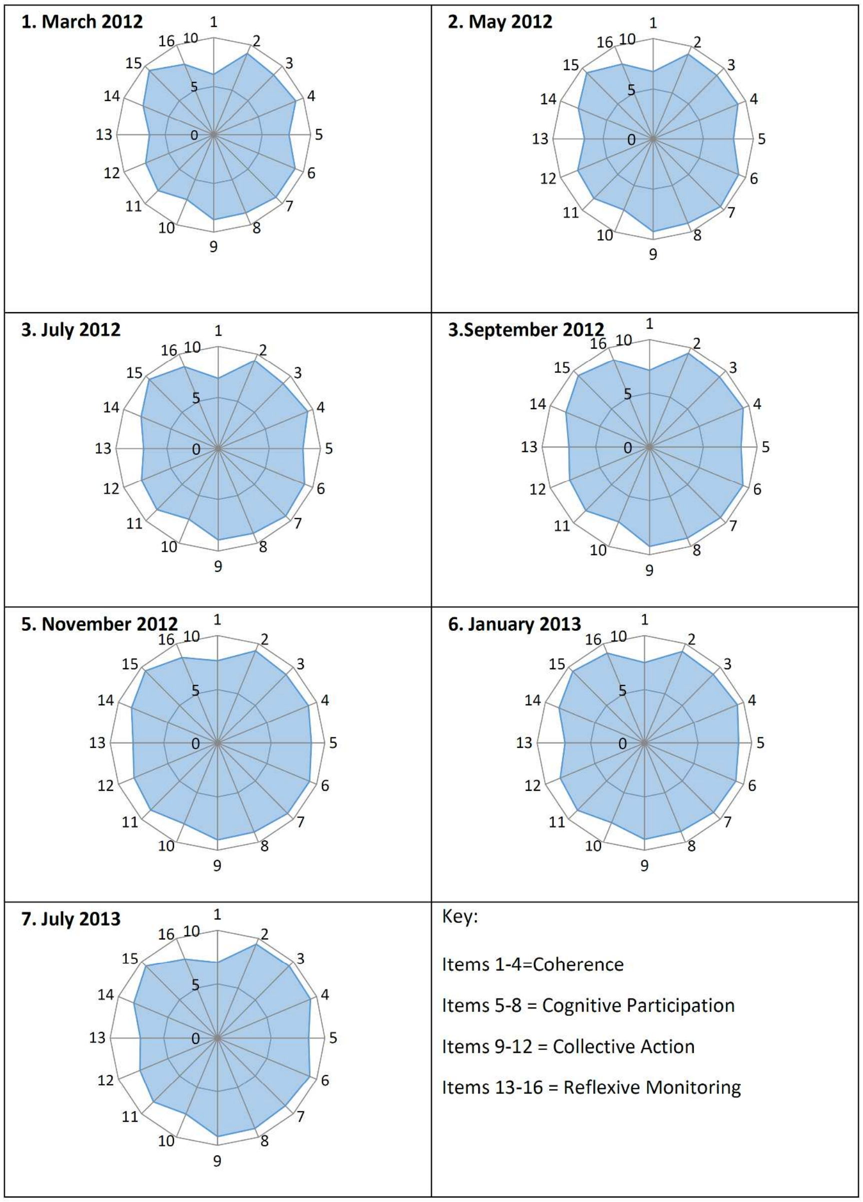

Figure 5: Radar plots showing the mean score for each sub-construct of NPT Results from the NPT questionnaire taken throughout the course of the study.

747x1039mm (96 x 96 DPI)

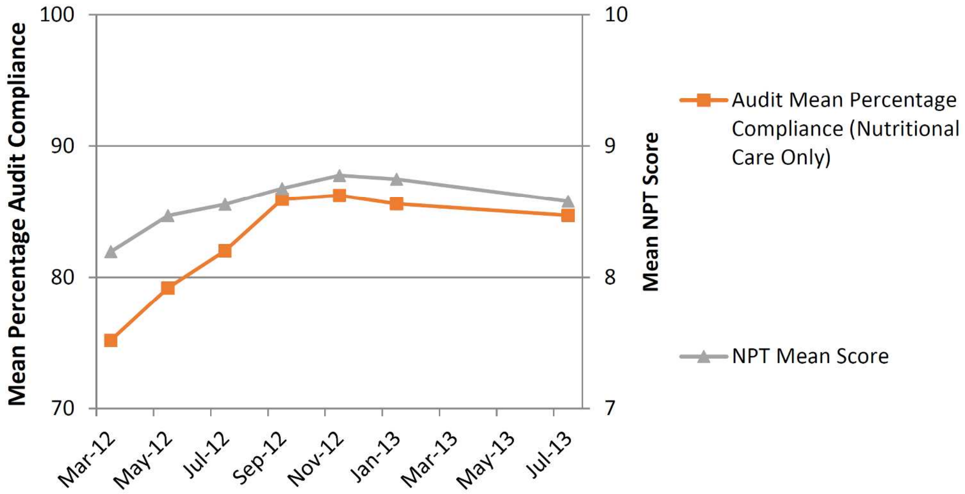

Figure 6: Relationship over time between mean NPT scores and percentage guideline compliance

702x365mm (96 x 96 DPI)

University Hospital Southampton NHS

Guidelines for the Nutritional Care of Infants in the Neonatal Unit

Version: 1.0

Issued: December 2011

Review date: December 2014

Author: Dr Alison Leaf

The procedural aspects of this guideline can be found in the document entitled:-

Guideline Proforma **- Guidelines for the Nutritional Care of Infants in the Neonatal Unit**

Disclaimer: It is your responsibility to check against StaffNet that this printout is the most recent issue of this document

For peer review only - http://bmjopen.bmj.com/site/about/guidelines.xhtml

University Hospital Southampton NHS
NHS Foundation Trust

Contents

Paragraph		Page
	**Executive Summary**	3
1.	Introduction	4
2.	Definitions	5
3.	Roles and Responsibilities	6
4.	Related Trust Documents	7
5.	Guideline	
	**1. Aims and objectives; audit points**	8
	**2. Assessment and monitoring**	
	i. Initial assessment: growth / risk category / screen	9
	ii. On-going assessment: growth / biochemistry / nutrition screening / nutrition team	10
	**3. Nutrient requirements**	11
	**4. Standard nutritional support of preterm and sick infants**	12
	a. Overview by risk group	12
	b. Parenteral nutrition	13
	i. Indications for PN	13
	ii. Starting PN	13
	iii. Stock PN solutions	13
	iv. Pharmacy-manufactured ('bespoke') PN	13
	v. Reducing PN as feeds increase	14
	vi. Peripheral PN	14
	vii. Cautions	14
	c. Enteral nutrition	15
	i. Starting feeds	15
	ii. Choice of milk	15
	iii. Advancing feeds	15
	iv. Routine nutritional supplements	16
	v. Nutrition at discharge	16
	**5. Management of common gut and feeding problems**	17
	a. Feed intolerance / gastric residuals	17
	b. Abdominal distension	17
	c. Suspected Necrotising Enterocolitis (NEC)	17
	d. Suspected Gastro-oesophageal reflux (GOR)	17
	e. Suspected Food Protein Intolerance	17
	**6. Management of babies with surgical bowel problems**	18
	**7. Flow Charts**	20
	a. Starting and Increasing Feeds – High Risk Infants	20
	b. Starting and Increasing Feeds – Moderate Risk Infants	21
	c. Management of common feed related problems	22
	d. Choice of milk	23
	**8. Tables**	24
	a. Starting and increasing feeds	24
	b. Nutrient content of commonly used products	25
	**9. Supporting Information**	26
6.	References	32
**Appendices**		
Appendix 1	Nutrient Requirements of preterm infants	35
Appendix 2	Neonatal Nutrition Screening Tool	36

Disclaimer: It is your responsibility to check against StaffNet that this printout is the most recent issue of this document

For peer review only - http://bmjopen.bmj.com/site/about/guidelines.xhtml

University Hospital Southampton NHS
NHS Foundation Trust

Executive Summary

Good nutrition is important at all stages of life. Babies are born at a time of rapid growth and formation of body tissues and organs, yet immature metabolism means they are unable to cope with either excess or lack of nutrients. Detail in both the quantity and quality of nutrients is critically important.

There is good evidence that mother's breast milk confers many advantages to baby, mother and to the formation of the parental bond. As well as containing just the right nutrients for human development, breast milk contains many factors which promote immune function and enable healthy intestinal development. Breast milk and breast-feeding should be encouraged in almost all situations.

Preterm infants and those with congenital abnormalities or metabolic disorders may require nutrient supplements or special feeds, and may require a period of intravenous nutrition until the gut is able to support their needs.

Measuring growth and monitoring biochemical well-being is crucial to optimising nutrition in high risk individuals.

These guidelines aim to provide both practical and theoretical guidance for the optimal nutrition of sick and preterm infants in the NNU at Southampton.

For peer review only

Disclaimer: It is your responsibility to check against StaffNet that this printout is the most recent issue of this document

For peer review only - http://bmjopen.bmj.com/site/about/guidelines.xhtml

University Hospital Southampton NHS
NHS Foundation Trust

1. Introduction

- Good early growth is essential for long term health and well-being of all babies.
- Achieving recommended nutrient intake in very low birth-weight and sick infants is difficult particularly in the first weeks of life and development of a significant nutrient deficit is common. It is then very difficult to 'catch up'.
- Protein intake is particularly difficult to achieve.
- These guidelines aim to support decision-making such that nutrient delivery can be optimised. Close monitoring of intakes, biochemical status and growth is essential to monitor how well this is achieved.
- ***Every feed and every day is important – being aware of daily intake of key nutrients is the first step to improving growth and development***
- SENNAT (Southampton Electronic Neonatal Nutrition Assessment Tool) has been developed to help us all measure and monitor nutrient intakes and growth

These guidelines are based on recommendations of:

- Enteral nutrient supply for preterm infants: commentary from the European Society of Paediatric Gastroenterology, Journal of Pediatric Gastroenterology and Nutrition 2010[1]

- Nutrition of the Preterm Infant: Scientific basis and Practical Guidelines (second edition). Tsang RC, Uauy R, Koletzko B, Zlotkin S. Digital Educational Publishing 2005[2]

- Guidelines on Paediatric Parenteral Nutrition of the European Society of Paediatric Gastroenterology, Hepatology and Nutrition (ESPGHAN) and the European Society for Clinical Nutrition and Metabolism (ESPEN), Supported by the European Society of Paediatric Research (ESPR), Journal of Pediatric Gastroenterology and Nutrition 2005[3]

- Vermont Oxford Network 'Potentially Better Practices (PBPs) for Nutrition' as laid out in Pediatrics, 2003[4]

- Management and support of infant feeding in maternity facilities. Infant and young child feeding : model chapter for textbooks for medical students and allied health professionals., World Health Organisation 2009[5]

- Optimal feeding of low-birth-weight infants, World Health Organisation, 2006[6]

- UNICEF Baby Friendly Initiative, http://www.unicef.org.uk/babyfriendly

Disclaimer: It is your responsibility to check against StaffNet that this printout is the most recent issue of this document

For peer review only - http://bmjopen.bmj.com/site/about/guidelines.xhtml

University Hospital Southampton NHS
NHS Foundation Trust

2. Definitions

AREDF	Absent or Reversed End Diastolic Flow (in umbilical artery, seen on antenatal scans)
AXR	Abdominal X-Ray
BMF	Breast Milk Fortifier
CPAP	Continuous Positive Airways Pressure
D/C	Discharge
DBM	Donor Breast Milk
DH	Department of Health
ELBW	Extremely Low Birth Weight (birth weight <1000g)
FBC	Full Blood Count
g	grams
IU	International Units
IUGR	Intrauterine Growth Restriction
IV	Intravenous
kcal	kilocalories
kg	kilogram
LBW	Low Birth Weight (birth weight <2500g)
LFT	Liver Function Tests
MBM	Maternal Breast Milk
mg	milligram
ml	millilitre
mmol	millimole
NBM	Nil By Mouth
NEC	Necrotising Enterocolitis
NICU	Neonatal Intensive Care Unit
NNU	Neonatal Unit
PBP	Potentially Better Practice
PDA	Patent Ductus Arteriosus
PDF	Post Discharge Formula
PN	Parenteral Nutrition
RCT	Randomised Controlled Trial
SD	Standard Deviation
TAT	Trans-anastamotic Tube
TPN	Total Parenteral Nutrition
U&E	Urea and Electrolytes
VLBW	Very Low Birth Weight (birth weight <1500g)
VON	Vermont Oxford Network

For peer review only

Disclaimer: It is your responsibility to check against StaffNet that this printout is the most recent issue of this document

For peer review only - http://bmjopen.bmj.com/site/about/guidelines.xhtml

3. Roles and Responsibilities

BREAST-FEEDING AND LACTATION SUPPORT

- All staff: awareness of Trust Policy and NNU Guidelines
- 'Breast-feeding babes' – Lead Sandy Jackson: expert guidance for mothers breast-feeding on the post-natal wards
- NNU lactation support team – Lead Jess Macfarlane: expert guidance for mothers breast-feeding and/or expressing milk in NNU

PARENTERAL NUTRITION

- All staff: awareness of need for PN in high risk infants
- Nursing staff: awareness of location of 'stock' PN in NNU and knowledge and skills for PN administration appropriate to nursing skill level
- Medical staff: awareness of PN supplies available and how to prescribe; awareness of potential complications of PN and how to avoid
- Pharmacists: Amanda Bevan and Zoe Lansdowne: expertise in detailed composition of PN solutions and provision of PN in different situations on NNU

ENTERAL NUTRITION

- All staff: support for mothers in choice of feeding
- All staff: awareness of choices for enteral nutrition: maternal breast milk / breast-feeding; donor breast milk / milk bank; standard infant formula; formulas for preterm infants; special formulas for infants with specific gut or feeding problems
- Neonatal Dietitian (Anita Emm): expert knowledge of composition of breast milk and alternatives and guidance on making appropriate choices
- Surgical team: expert knowledge on potential feeding challenges in infants with congenital or acquired abnormalities of the gut, particularly following surgery.

FEEDING DIFFICULTIES

- All staff: awareness of common feeding difficulties of preterm infants and those with neurological complications
- Speech and language therapist: expert knowledge of structure and function of upper gastro- intestinal tract and how to optimise feeding potential of vulnerable babies

GROWTH MONITORING

- All staff: Awareness of importance of making accurate and regular measurements and plotting them on appropriate charts to monitor growth
- Nursing staff: Weigh babies at intervals as indicated by clinical condition (ideally three times per week)
- Medical and Nursing staff: Measure head circumference and length at intervals as indicated by clinical conditions (ideally head circumference at least weekly and length at least fortnightly)
- Medical and Nursing staff: Plot growth measurements on appropriate chart weekly (provided competent to do so)

SPECIAL CASES

- Neonatal Nutrition Team: Will review high risk or complex patients on weekly nutrition ward round

Disclaimer: It is your responsibility to check against StaffNet that this printout is the most recent issue of this document

For peer review only - http://bmjopen.bmj.com/site/about/guidelines.xhtml

University Hospital Southampton NHS
NHS Foundation Trust

4. Related Trust Documents

Donor Breast Milk Guideline (to be found at: http://staffnet/TrustDocsMedia/DeptDivSpecific/DivC/WomenNewborn/NeonatalUnit/NeonatalGuidelines/DonerBreastMilkGuideline/DonorBreastMilkGuideline.doc)

Breastfeeding care pathway (on Neonatal Unit Guidelines on Unit Desktop PCs)

Vitamins and supplements guideline (on Neonatal Unit Guidelines on Unit Desktop PCs)

Parenteral Nutrition Guidebook, 4th edition (Hard copies in nurseries on Neonatal Unit)

Princess Anne Breastfeeding Policy (to be found at http://staffnet/TrustDocsMedia/DeptDivSpecific/DivC/WomenNewborn/Obstetrics/ObstetricClinicalGuidelines/BreastfeedingTermInfantsGuideline/BreastfeedingTermInfantsGuideline.doc)

Neonatal Unit Breastfeeding and Formula Feeding Guideline (currently being written)

Neonatal Surgical Clinical Aids (to be found at: http://staffnet/Departments/DivisionC/Womenandnewborn/Neonatalservices/Neonatalsurgery/Neonatalsurgeryclinicalaids/Neonatalsurgeryclinicalaids.aspx)

Central Venous Access Guideline (currently being written)

Naso/Orogastric Tubes in Neonates - the safe placement of: Guidelines (to be found at:http://staffnet/TrustDocsMedia/DeptDivSpecific/DivC/WomenNewborn/NeonatalUnit/NeonatalGuidelines/NasoOrogastricTubesinNeonates-thesafeplace/NasoOrogastricTubesinNeonates-thesafeplacementofGuidelines.DOC)

Disclaimer: It is your responsibility to check against StaffNet that this printout is the most recent issue of this document

For peer review only - http://bmjopen.bmj.com/site/about/guidelines.xhtml

5. Guideline Information

1. AIMS AND OBJECTIVES

- To optimise use of breast milk and breast-feeding
- To achieve recommended nutrient intakes
- To achieve postnatal growth and body composition approximating fetal growth.
- To reduce the risk of nutritional deficiency states such as late anaemia of prematurity or metabolic bone disease.
- To reduce the risk of feeding related morbidities such as NEC or cholestasis
- To optimise long term neurodevelopmental outcome.

KEY PRINCIPLES

- All babies should be measured and have nutritional risk assessment on admission, and weekly during their stay
- Nutrition support should be started early: PN for high risk; enteral feeds for lower risk
- Mother's breast milk is the feed of first choice
- Feed tolerance should be assessed regularly and managed according to algorithms
- Protein intake should be documented and optimised in preterm infants
- High risk babies should be seen each week by the Nutrition Team
- Nutrition and feeding should be discussed in Discharge Planning and documented in the notes

AUDIT POINTS

- Use of Nutrition Screening Tool, on all NNU admissions (100%)
- Use of growth charts on all NNU admissions (100%)
    - Weight and Head Circumference plot weekly; length plot 2-weekly
- Lactation advice and support by 6 hours for all mothers of VLBW infants
    - 100% - unless mother too ill
- Breastfeeding rates at discharge
- Protein and energy intakes as recommended by Tsang 2005
- Use of nutritional supplements according to Guidelines
- Documentation of Nutrition Plan at discharge (100%)

Disclaimer: It is your responsibility to check against StaffNet that this printout is the most recent issue of this document

For peer review only - http://bmjopen.bmj.com/site/about/guidelines.xhtml

University Hospital Southampton NHS
NHS Foundation Trust

2. ASSESSMENT AND MONITORING

(i) INITIAL ASSESSMENT

a. <u>Growth Measurement</u>

All infants should have weight, length and head circumference measured and plotted on the appropriate growth chart at admission. This information, together with other risk factors detailed below, will identify the degree of 'nutritional risk' – ie risk of becoming malnourished or developing nutrition and feeding related problems. Infants with multiple risk factors should be classified according to their highest individual risk factor. This will guide nutritional care and allow subsequent progress to be monitored.

b. <u>Risk assessment</u> – identify level of risk for nutrition and / or feeding-related problems

High risk

- Preterm <28 weeks
- ELBW < 1000g
- Severe IUGR (weight < 2nd centile with AREDF) <35 weeks
- Infant establishing feeds after episode of NEC or GI perforation
- Infants with severe congenital GI malformation: e.g. gastroschisis
- Severe Perinatal hypoxia / ischaemia

Moderate risk

- Preterm 28-31^{+6} weeks, otherwise well
- VLBW 1000 – 1500g
- Moderate IUGR (weight < 9th centile with AREDF) <35 weeks
- Baby on inotropes
- Baby on indomethacin/ibuprofen (NB avoid concomitant treatment with steroids)
- Baby >1500g with illness or congenital anomaly which may compromise feeding
- Symptomatic polycythaemia, with PCV $\geq$ 70%

Low risk

- Preterm 32-36^{+6} weeks, otherwise well
- AREDF / IUGR $\geq$35 weeks
- Term Infants >37 weeks

Disclaimer: It is your responsibility to check against StaffNet that this printout is the most recent issue of this document

For peer review only - http://bmjopen.bmj.com/site/about/guidelines.xhtml

(ii) ON-GOING ASSESSMENT AND MONITORING

a. GROWTH

i. Weight should be measured at least twice a week, and plotted on CLOSE MONITORING WHO growth chart weekly. More frequent weights required for some babies should be plotted on a daily weight chart
ii. Head circumference should be measured and plotted weekly
iii. Length should be measured and plotted within the first week, and every 2 weeks thereafter.
iv. If a baby is too sick to be weighed and measured so cannot be plotted, mark the bottom of the growth chart at date with a triangle (△) at the day's date.
v. Targets for weight – changes in weight in the early days of life usually reflect fluid balance: aim for weight loss of no more than 10% from birth weight. Once baby is stable and growing, aim for gain of 15-20 grams/kg/day
vi. Head circumference and length: normally expect increase of 0.75 cm/week

b. BIOCHEMISTRY

i. **First week of PN**:
   - Full TPN Profile daily (FULL IP MG on eQuest, this includes U&E's, Calcium, magnesium phosphate and LFTs)
   - FBC twice weekly

ii. **Second and subsequent week of PN**:
   - Full TPN Profile and FBC twice weekly if stable (daily if still unstable)

iii. **Triglycerides** should be measured weekly (ideally Mondays)when on IV lipid
iv. **If on PN for longer than 1 month, then Trace elements (Zn, Cu, Se, Mn – use special blood bottle in Dr's Office) and Vitamins (A, D and E) should be measured monthly. Consider measuring Iron status and clotting**
v. **When on enteral feeds:**
   - Infants in the High and Medium risk categories need weekly FBC, U&Es, LFTs and Bone profiles once they are off PN and fully enterally fed. This can be extended to once fortnightly when babies are moved into Special Care.

c. SCREENING

i. A Neonatal Nutrition Screening form should be completed on admission and on Sunday/Monday when the baby has been weighed and measured each week on all babies to identify those requiring nutrition team review

d. NUTRITION TEAM REVIEW

i. Nutrition ward rounds take place on Tuesday mornings from 0900-1100. Nutrition team will see all 'high-risk' babies, and any others identified by nutritional screening on Sunday/Mondays.

For peer review only - http://bmjopen.bmj.com/site/about/guidelines.xhtml

3. NUTRIENT REQUIREMENTS

Nutrient requirement for Term and Preterm infants in the first weeks of life are summarised below. The figures shown below are based on the parenteral requirements for the first week, and the enteral requirements for the subsequent weeks (for a full list of parenteral and enteral requirements see Appendix 1).

Term infants – based on intake in 150 ml/kg breast milk; preterm infants based on recommendations in Tsang 2005 unless otherwise stated.

There are no specific guidelines for those babies born over 1.5kg and under term weight (2.5 kg) but it can be anticipated that their nutritional needs will be between those of preterm infants and term infants. Nutritional support should therefore aim to deliver nutrient intakes in this area.

It should be noted that these are just recommendations, and some infants may require more of certain nutrients such as Sodium and Potassium as dictated by the results of blood tests.

**Nutrient Unit/kg/day**	**Term infant**	**Preterm VLBW 1000-1500g 1st week (parenteral)**	**Preterm VLBW 1000-1500g After 1st week (enteral)**	**Preterm ELBW < 1000g 1st week (parenteral)**	**Preterm ELBW < 1000g After 1st week (enteral)**
Energy (kcal)	100	60-70	110-130	75-85	130-150
Protein (g)	1.5-2.1	3.5	3.4-4.2	3.5	3.8-4.4
Nitrogen (g)	0.24-0.34	0.56	0.54-0.61	0.56	0.61-0.70
Sodium (mmol)	1.4	2.0-5.0	3.0-7.0	2.0-5.0	3.0-7.0
Potassium (mmol)	2.0	0-2.0	2.0-3.0	0-2.0	2.0-3.0
Calcium (mmol)	1.25	1.5	2.5-5.5	1.5	2.5-5.5
Phosphate (mmol)	1.3	1.5-1.9	1.9-4.5	1.5-1.9	1.9-4.5
Vitamin D IU*	340	40-160	800-1000	40-160	800-1000
Vitamin A IU**	1150	700-1500	700-1500	700-1500	700-1500
Iron (umol)	17.9	0	35.8-71.6	0	35.8-71.6

*Vitamin D = dose quoted is total daily dose; ESPGHAN 2010 recommendation for enteral dose for preterm infants; term infants DH Dietary Reference Values 1991 (340 IU = 8.5 mcg Vit D)

**Vitamin A = dose quoted is total daily dose; term infants DH Dietary Reference Values 1991 (1150 IU = 350 mcg of Vitamin A retinol equivalent)

For peer review only - http://bmjopen.bmj.com/site/about/guidelines.xhtml

Clinical Guidance

University Hospital Southampton NHS
NHS Foundation Trust

4. STANDARD NUTRITION SUPPORT –

(a) OVERVIEW - GETTING STARTED - EARLY TPN AND TROPHIC MILK FEEDS

HIGH RISK / MEDIUM RISK (see flow charts for high [A] and medium risk preterm infants [B])

- Aim to introduce milk feeds gradually while maintaining calorie and nutrient intake with PN
- Before starting or increasing milk ensure baby is clinically stable and abdomen soft
- Ensure mother has lactation support to start expressing (see breastfeeding care pathway)

High risk preterm (<28 weeks; <1000g; severe IUGR/AREDFV <35 weeks)

Day 1	Start Stock Preterm PN at 60-90 ml/kg/day via UVC or long line, as soon as possible unless baby very unstable. Give fresh colostrum as mouth care or as trophic feeds
Day 2-3	Start trophic feeds: MBM 1 ml/kg 2-4 hourly (if no MBM can use DBM- see choice of milk chart);
Day 3-7	Change to Stock Preterm + Sodium PN when 6% weight loss from birthweight [7], additional sodium required, or by day 5, whichever soonest Increase milk by 10-20 ml/kg/day as tolerated (see table); Aim to decrease PN flow rates with feeds only once baby on total fluids of 180ml/kg/day

Moderate risk preterm (28-31^{+6} weeks; 1000g <1500g; mod IUGR/AREDFV < 35 weeks)

Day 1	Start Stock preterm PN at 60-90 ml/kg/day via UVC or long line as soon as possible; if no central access consider peripheral PN
Day 1-2	Start colostrum/milk 1 ml/kg 2 hourly ('see choice of milk' chart)
Day 3-7	Change to Stock Preterm + Sodium PN when 6% weight loss, or by day 5, whichever is sooner. Aim to decrease PN flow rates with feeds only once baby on total fluids of 180ml/kg/day Increase milk by 20-30 ml/kg/day according to clinical condition and tolerance;

High / moderate risk term or near-term infants

All high/moderate risk babies should have a plan for nutrition support on admission and periods greater than 48 hours without protein and micronutrients should be unusual

Low risk

Day 1	Commence milk feeds 30-60 ml/kg/day, supplemented by IV fluids if necessary
Day 2-7	Increase milk feeds by 30 ml/kg/day as tolerated

NOTES

- If severely unwell or acidotic, PN may need to be delayed (though contains acetate)
- Babies with HIE undergoing therapeutic hypothermia, may tolerate trophic milk feeds
- For babies with surgical problems , see 'surgical guidelines' – section 6

Disclaimer: It is your responsibility to check against StaffNet that this printout is the most recent issue of this document

For peer review only - http://bmjopen.bmj.com/site/about/guidelines.xhtml

4. (b) PARENTERAL NUTRITION

i) Indications for PN

- High or Moderate risk infants as described above
- Infants who are NBM and unlikely to achieve adequate milk intake in the next 5 days
- Infants who are not tolerating feeds such that they cannot take full feed volumes for 5 consecutive days

ii) Starting PN

- In high and moderate risk infants PN should be started as soon as possible as delay can result in significant and cumulative nutrient deficits.
- Birth weight ≤1500g – start as soon as possible after birth
  - Ideally within 6 hours
- Birth weight >1500g – if enteral feeding contra-indicated, start PN by
  - 48 hours in 1500-2500g
  - 72 hours in 2500-3500g if NBM
- Central line insertion (UVC or peripherally inserted central venous line) should be a priority for high and moderate risk infants
- If feeds are stopped on high or medium risk infant for any reason, re-stat PN

iii) Stock PN

- Infants should be started on Stock PN in the first instance as detailed below:
  - Preterm PN – For preterm infants (<37/40 gestation) where additional sodium is not indicated (ie until 6% weight loss, or day 5 of life)
  - Preterm + Sodium PN- For preterm infants (<37/40 gestation) requiring maintenance sodium. **This should be the PN of choice for the majority of preterm infants after the first few days following birth**, as it contains more protein.
  - Term PN – for Term infants (≥37 weeks gestation) at any point after birth.
- Stock PN comprises an aqueous solution (glucose, amino acids, electrolytes and trace elements) and a lipid solution (**which contains both fat- and water-soluble vitamins**). For adequate nutrition it is **important that the lipid is always given with the aqueous solution** at all times (except when well advanced on enteral feeds - see below).

iv) Pharmacy made ('bespoke') PN

- Neither PN alone nor unfortified full breast milk feeds fully meet the nutritional needs of preterm infants, so the period when a preterm infant transitions from PN to milk feeds is when they are at highest risk of poor nutrient intakes.
- Stock PN is designed to give the maximum possible nutrition at 130ml/kg/day. **Therefore, pharmacy can make bespoke PN, which provides more nutrition in a smaller volume, should be used whenever a preterm infant is receiving less than 130ml/kg/day of Stock PN**. This will occur whenever a preterm infant is increasing on enteral feeds, is fluid restricted, or receiving other infusions
- Bespoke PN may also be appropriate where infants have electrolyte requirements than cannot be met with Stock PN

Disclaimer: It is your responsibility to check against StaffNet that this printout is the most recent issue of this document

For peer review only - http://bmjopen.bmj.com/site/about/guidelines.xhtml

v) Reducing PN as enteral feeds increase

- **Only once the infant is receiving 180ml/kg/day total fluids should the PN solution be decreased as enteral feeds increase** (unless there is a clinical decision to restrict fluids).
- Once the infant is on 90ml/kg/day enteral feeds, the rate of lipid infusion should be halved, and then stopped when the infant reaches 135ml/kg/day enteral feeds (beware with pharmacy made TPN as this reduction in lipid may have already been done as part of the prescription). Any shortfall in total fluid volume due to the reduction in lipid should be made up by increasing the aqueous PN solution, to allow maximum protein to be delivered to the infant (though do not go above the maximum prescribed rate). This is important when infants are on Stock PN, but for those on bespoke PN, the reduction in lipid may have already been done/accounted for by the pharmacists when the PN was prescribed so may not be necessary (check with the pharmacists first). **Remember that once the lipid is stopped, vitamin intake will be inadequate until Abidec is started.**

vi) Peripheral PN

- PN should ideally be given via a central line. However, there are occasions in high nutritional risk infants with difficult access where the benefits of giving PN peripherally may outweigh the risks. Such decisions should be made by the Consultant responsible for the patient.

vii) Cautions on PN

SEPSIS - may affect lipid metabolism; measure triglycerides and if >2.8mmol/L consider reducing or stopping IV lipid for 12-24 hours in severely septicaemic baby (remember to restart/increase lipid when sepsis has resolved)

THROMBOCYTOPENIA – high concentration of polyunsaturated fats may impair platelet adhesion: reduce lipid to 1-2 g/kg/day if platelets <50.

CHOLESTATIC JAUNDICE – total and prolonged PN increases the risk, so try to give some enteral feed if at all possible; other risk factors include IUGR, sepsis and short bowel syndrome. Lipid solutions containing fish oil (eg SMOF) can reduce or reverse cholestasis, and should be considered in high risk babies if on PN for 4 weeks or more. Alternate day lipid may also be indicated in this situation, or if altered liver function - discuss with the pharmacists.

Disclaimer: It is your responsibility to check against StaffNet that this printout is the most recent issue of this document

For peer review only - http://bmjopen.bmj.com/site/about/guidelines.xhtml

University Hospital Southampton NHS
NHS Foundation Trust

4 (c) ENTERAL NUTRITION

i. **Starting feeds** – see section 4(a) for guidance. Before starting feeds ensure baby is clinically stable and abdomen soft. In high-risk infants trophic feeding should be started within the first 72 hours if at all possible to minimise intestinal mucosal atrophy, and continued until ready to progress.

ii. **Choice of milk** – Mother's breast milk is almost always the feed of first choice, unless contraindicated by maternal illness or drugs. If no maternal milk available pasteurised donor breast may be used for high risk babies (parental consent required) in accordance with the DBM guideline. Preterm formula (LBW/Aptamil Preterm) is indicated for infants with gestation <34 weeks, or birth weight <1800 grams; Post discharge formula (Nutriprem 2) is indicated for preterm infants either as sole diet or in addition to breast-feeding from around 36 weeks (or at discharge) up to 6 months corrected. (see Flow Chart D)

iii. **Advancing feeds** – see section 4 (a) for guidance on volumes

- Before starting or increasing milk ensure baby is clinically stable and abdomen soft. Small gastric residuals can be tolerated if baby well. Passage of meconium and then changing stools is an important indication of gut motility. Glycerine suppositories may be useful if no stool passed for 48 hours.
- Feeds can be increased by 10-20ml/kg/day in high-risk, 20-30ml/kg/day in moderate risk and 30 ml/kg/day in low risk babies
- Test for residuals 4-6 hourly
- If baby vomits, or has residuals >25% of the previous 4 hours total feed volume and persisting or increasing examine and assess baby and refer to flow chart C

iv. Nutritional supplements

- BREAST MILK FORTIFIER (BMF, see high risk and moderate risk flow charts A and B) - 'multi-component' fortifier provides additional calories (carbohydrate), protein (cows' milk based), minerals and vitamins in a powder which is added to mother's breast milk. It should be more or less routine for babies with birth weight <1500g to receive fortifier once they have tolerated 150 mls/kg/day of MBM for 24 hours, unless significant gut or renal compromise. Blood Urea and albumin levels provide useful markers of protein status. In general, give ½ strength for 24-48 hours and then increase to full strength (2.2g sachet to 50 mls MBM), though it may be preferable to increase the fortifier by ¼s in high risk infants. For some extremely high risk infants it may be prudent to start fortifier when on 120-135 mls/kg/day of MBM and increase strength more gradually as PN is gradually reduced, in order to ensure the baby will be able to achieve enteral nutrient targets before stopping PN.

Disclaimer: It is your responsibility to check against StaffNet that this printout is the most recent issue of this document

For peer review only - http://bmjopen.bmj.com/site/about/guidelines.xhtml

- Vitamins and Iron – breast milk provides insufficient vitamins (particularly vitamin A and D) for preterm infants, and virtually no iron. Abidec (multivitamins) and Sytron (iron) should be started according to NNU guideline
- Electrolytes and minerals
  - Small doses should be given as boluses, as scheduled on drug chart
  - Sodium : aim to maintain serum sodium 135-145 mmol/L
    If on > 4 mmol/kg/day, add to daily feeds in milk kitchen; if < 4 mmol/kg/day, give as divided bolus drugs (ideally as a four times daily regimen)
  - Phosphate: content of BM is low. Aim to maintain serum inorganic phosphate levels greater than 1.8mmo/L. Usually given as Potassium Acid Phosphate 0.5-2mmol/kg/day. If required as outpatient, may be preferable to use BMF

v. Nutrition at discharge

It is important to start discharge-planning well in advance. Breast-feeding at discharge is the preferred goal for all infants. However for preterm infants nutritional supplementation will be required. For those not being breast fed advice has to be given on choice of formula, so for all infants a pre-discharge nutrition assessment should be made and plan documented.

MUM PLANNING TO BREAST FEED

- Ensure lactation support is on-going re feeding technique
- Discuss with Out-reach sister re support at home
- **All preterm infants (<35 weeks) should have Abidec (1 ml) and Sytron (1 ml) daily**
- Assess growth
  - If growth has been good and weight, length and HC are no more than 0.67 SD (ie one centile line) below birth levels, then assess weight gain after 48 hours. If satisfactory can go home breast-feeding
  - If baby has had significant post-natal growth restriction and is >1.33 SD below birth (2 centile lines), discuss with Nutrition team / Dietician and consider discharge on BMF, with Outreach Support
  - For those with modest growth restriction, i.e. between one and two centile line drop, review overall pattern of growth and consider requesting nutrition review and Outreach support.

MUM PLANNING TO FORMULA FEED

- Babies <34 weeks gestation, with birthweight <2kg can be considered for discharge on Post-Discharge Formula (PDF) – 'Nutriprem 2'. This should be continued until 3 to 6 months corrected age.
- ELBW and VLBW babies who have been on LBW formula should be changed to PDF at approximately 36 weeks corrected age, or when beginning to take most feeds by bottle. For those who have had severe extra-uterine growth restriction, continuing with LBW formula to 40 weeks corrected age may be appropriate.
- Babies discharged on PDF should have Abidec 0.6 ml, but not Sytron.
- If changing to term formula, prescribe Abidec 1 ml (continue until at least one year post term) and Sytron1ml (continue until 6 month post term)

SOLIDS **–** can be introduced at 5-8 months REAL AGE (ie not corrected for prematurity)

Disclaimer: It is your responsibility to check against StaffNet that this printout is the most recent issue of this document

For peer review only - http://bmjopen.bmj.com/site/about/guidelines.xhtml

5. MANAGEMENT OF COMMON GUT AND FEEDING PROBLEMS – see flow chart C

a. **Gastric aspirates / residuals** – preterm infants have immature gut motility, and aspirates/residuals and small vomits are not uncommon. Dark green bile stained aspirates, particularly in association with abdominal distension and / or tenderness are a cause for concern. However small milky / yellow aspirates up to 2-3 mls are frequently normal. They can be replaced, and feeds continued.

b. **Abdominal distension** – this is another common feature in preterm infants, due to poor gut motility. It tends to be more common in babies on nasal CPAP, with high volumes of air flowing into the upper airway and oesophagus. Tenderness, or systemic symptoms and signs such as apnoea, tachycardia or temperature instability should raise concern. If baby is otherwise well, a small glycerine suppository may help to stimulate peristalsis, and enable feeds to be continued.

c. **Suspected NEC** – classical features are blood and mucous in stools, bile stained aspirates and abdominal tenderness. Systemic signs such as tachycardia and hypotension occur in severe NEC. X-ray might show intramural gas (‘pneumatosis coli’), dilated loops of bowel, free air, or a ‘gas-less’ bowel. In suspected NEC feeds should be stopped, and urgent attention paid to supporting ventilation, circulation and fluid balance.

d. **Suspected GOR** – mild milk reflux is common in newborn babies, including those born preterm and is usually self-limiting. It is rarely the cause of significant cardio-respiratory disturbance. However, apnoea and bradycardia are common in preterm babies and may occur in association with feeds. Try to avoid using gaviscon in babies who are having fortified MBM as the milk becomes excessively thick.

e. **Suspected Food Protein Intolerance** – food protein (e.g. cow’s milk protein) intolerance can occur in young infants either breast fed or formula fed. Symptoms may include severe regurgitation, vomiting, constipation, peri-anal rash, blood in stools and iron deficiency anaemia. Non-intestinal features may include skin rash – atopic eczema, and colic. If this is thought to be the cause of symptoms, it is recommended that cow’s milk protein be excluded from diet. If breast feeding, mother should exclude both cows’ milk and egg products from her diet for two weeks, while continuing to breast feed. Formula fed infants should be tried on amino acid formula. If improvement is seen, a staged reintroduction should be carried out. If no improvement is seen on definite exclusion diet, food protein intolerance is unlikely. If exclusion diet is difficult to maintain, a trial of amino-acid formula may be breast fed infants. See review by Vandenplas et al.[8]

Disclaimer: It is your responsibility to check against StaffNet that this printout is the most recent issue of this document

For peer review only - http://bmjopen.bmj.com/site/about/guidelines.xhtml

University Hospital Southampton NHS
NHS Foundation Trust

6. MANAGEMENT OF BABIES WITH SURGICAL BOWEL CONDITIONS WHICH MAY COMPROMISE NUTRITION

Information has been extracted from the NEONATAL SURGERY CLINICAL AIDS on SUHTranet:

(http://staffnet/Departments/DivisionC/Womenandnewborn/Neonatalservices/Neonatalsurgery/Neonatalsurgeryclinicalaids/Anorectalmalformations.aspx)

This website should be checked to ensure that the most up to date version of the guidance is used.

GASTROSCHISIS

All babies with gastroschisis will require TPN.

For those treated with a Medicina Silo insertion at the cot-side a percutaneous long line should be sited on the Neonatal Unit but line insertion should ideally be delayed until after gut manipulation has ceased, i.e. once the silo has been removed and the defect closed, to reduce the chance of line colonisation. The median time to closure is 4 days. If it is felt that TPN should be commenced before this time then this can be given via peripheral cannula. In babies in whom it is thought there may be a delay in defect closure it may be better to proceed with line insertion prior to closure. As some gastroschisis babies may go on to have intestinal failure and require long term central venous access, central lines should only be inserted by staff with considerable experience of line insertion so as to avoid loss of suitable veins.

If the baby is taken to theatre for primary closure or surgical silo creation a percutaneous long line can be inserted in theatre at the time if someone with the appropriate expertise is available.

Duration of TPN may vary from 10 days to 6 weeks with a mean of 3 weeks. In rare cases gut function may be impaired for many months.

DUODENAL ATRESIA

A trans-anastamotic tube (TAT) can be placed during surgery, which allows feeding into the jejunum. A naso/orogastric tube will also be required for gastric decompression. Usually a 6Fr enteral feeding tube is placed nasojejunally and an 8Fr nasogastric tube placed down the other nostril. In preterm babies this may produce problems due to obstruction to both nostrils. In this situation it may be better to pass an orogastric 8Fr tube and leave one nostril patent.

Poor duodenal contractility may delay normal oral feeding for as long as 3 weeks. This may be overcome by transanastamotic feeding although there is evidence that this may delay eventual oral feeding. It is NOT usually necessary to place a long line or commence TPN because of the use of TAT feeding. Duration of admission is about 7 - 10 days but may be longer if motility is very delayed.

EXOMPHALOS

Nutritional support: Most babies who have undergone primary closure will tolerate enteral feeding soon and not need TPN. Most babies with a silo will require a long line and TPN

For peer review only - http://bmjopen.bmj.com/site/about/guidelines.xhtml

University Hospital Southampton NHS
NHS Foundation Trust

MECONIUM ILEUS

Feeding may start when gut recovery from surgery allows. Usually start on MBM or standard formula feed grading up slowly. Feed may need to change to hydrolysed formula if weight gain inadequate on breast milk or standard formula. Occasionally TPN is needed.

80-90% of babies with MI are deficient in pancreatic enzymes, and supplementation with 'Creon®' may be required. Further details are provided in Surgical Clinical Aids and treatment will usually be guided by advice from the CF team

OESOPHAGEAL ATRESIA and TRACHEO-OESOPHAGEAL FISTULA

A trans-anastomotic tube (TAT) nasogastric tube will be placed at time of surgery and feeding usually commences via the TAT at 48hrs post-op. If the TAT falls out do not re-pass as this may perforate the anastomosis. Consult the surgical team immediately.

Oral feeding normally starts between 3 and 5 days post-op at the discretion of the surgical team.

Gastro-oesophageal reflux prophylaxis: some surgeons use ranitidine post-op for 3 - 6 months. Others do not.

For peer review only

Disclaimer: It is your responsibility to check against StaffNet that this printout is the most recent issue of this document

For peer review only - http://bmjopen.bmj.com/site/about/guidelines.xhtml

7. FLOW CHARTS

a. Starting and Increasing Feeds- HIGH RISK INFANTS

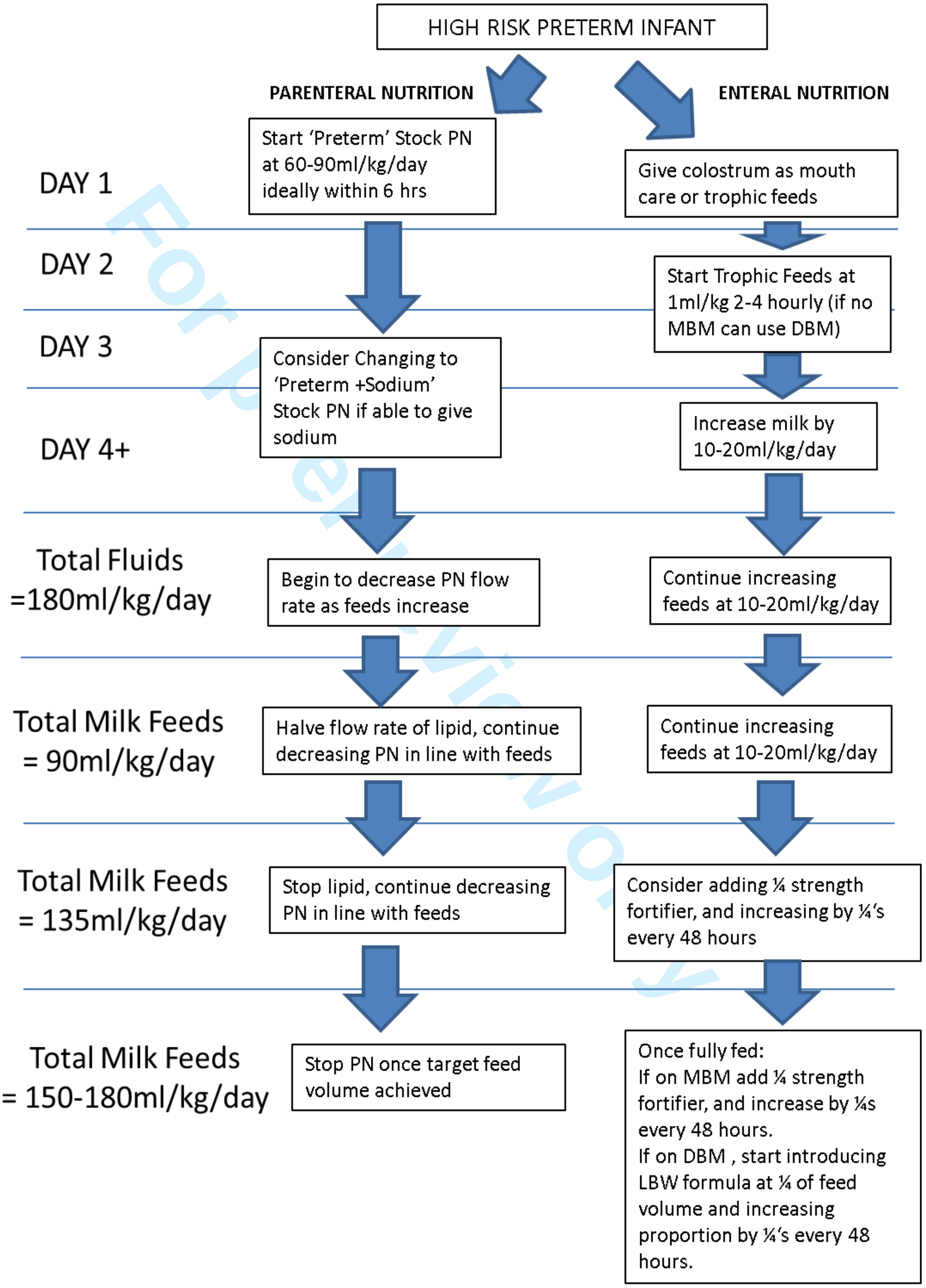

For peer review only - http://bmjopen.bmj.com/site/about/guidelines.xhtml

b. **Starting and Increasing Feeds- MODERATE RISK INFANTS**

MODERATE RISK PRETERM INFANT

	**PARENTERAL NUTRITION**	**ENTERAL NUTRITION**
DAY 1	Start 'Preterm' Stock PN at 60-90ml/kg/day ideally within 6 hrs	Start Trophic Feeds at 1ml/kg 2 hourly (if no MBM can use DBM)
DAY 2		
DAY 3	Consider Changing to 'Preterm +Sodium' Stock PN if able to give sodium	
DAY 4+		Increase milk by 20-30ml/kg/day
Total Fluids =180ml/kg/day	Begin to decrease PN flow rate as feeds increase	Continue increasing feeds at 20-30ml/kg/day
Total Milk Feeds = 90ml/kg/day	Halve flow rate of lipid, continue decreasing PN in line with feeds	Continue increasing feeds at 20-30ml/kg/day
Total Milk Feeds = 135ml/kg/day	Stop lipid, continue decreasing PN in line with feeds	
Total Milk Feeds = 150-180ml/kg/day	Stop PN once target feed volume achieved	Once fully fed: If on MBM add ½ strength fortifier, and increase to full fortifier 24-48 hours. If on DBM , start introducing LBW formula at ¼ of feed volume and increasing proportion by ¼'s every 48 hours.

For peer review only

Disclaimer: It is your responsibility to check against StaffNet that this printout is the most recent issue of this document

For peer review only - http://bmjopen.bmj.com/site/about/guidelines.xhtml

University Hospital Southampton NHS
NHS Foundation Trust

b. Management of common feed-related problems

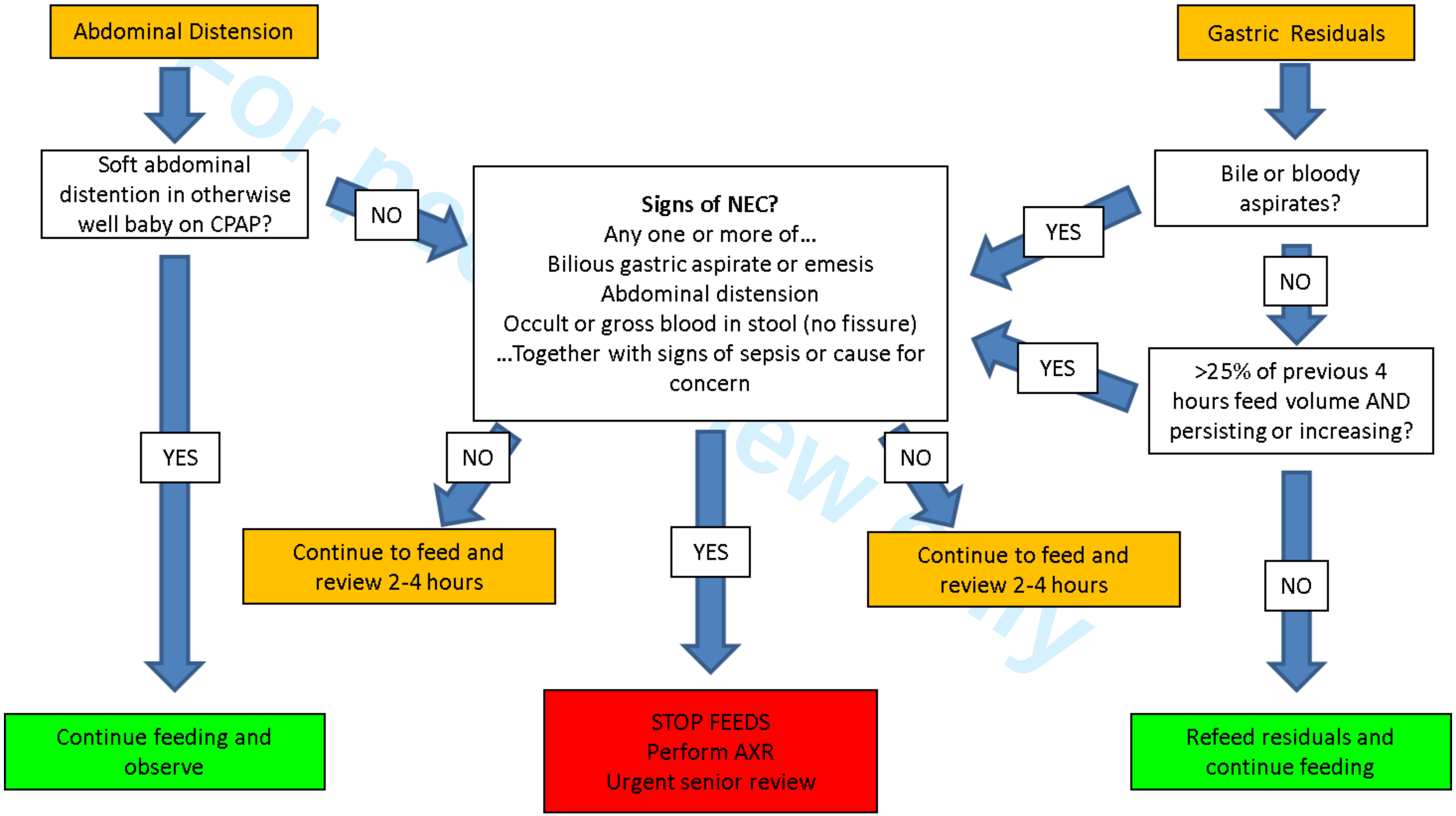

For peer review only - http://bmjopen.bmj.com/site/about/guidelines.xhtml

Clinical Guidance

University Hospital Southampton NHS
NHS Foundation Trust

c. Choice of Milk

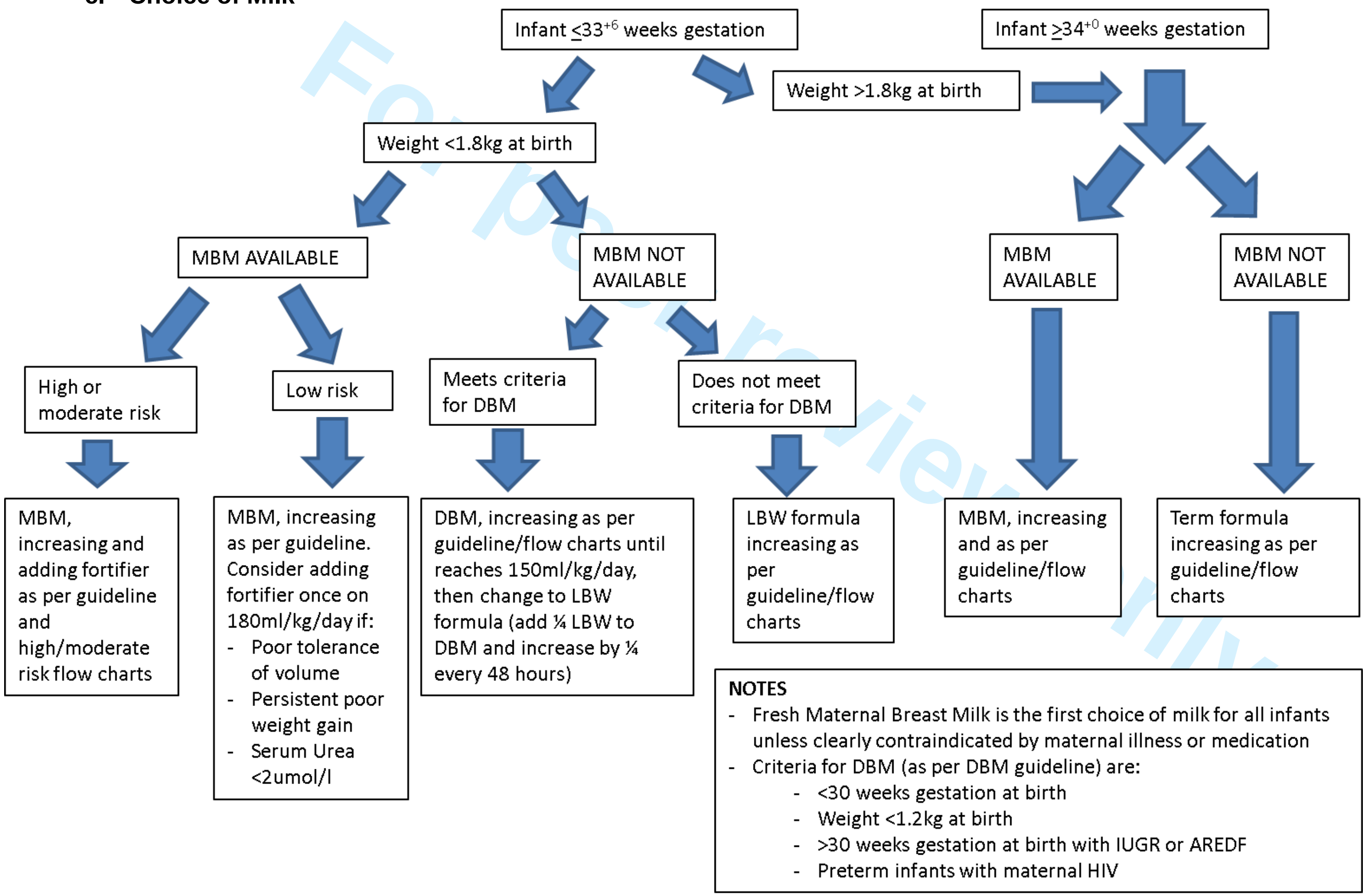

Disclaimer: It is your responsibility to check against StaffNet that this printout is the most recent issue of this document

For peer review only - http://bmjopen.bmj.com/site/about/guidelines.xhtml

8. TABLES

a. Starting and Increasing Feeds

i. High Risk Infants (based on increases of 10-20ml/kg/day)

Weight (kg)	Start at (hourly)	Start at (2 hourly)	Increase hourly feed volume by*	Increase 2hourly feed volume by
less than 0.6	N/A	0.5	0.25ml every 24 hours	0.5ml every 24 hours
0.6-0.9	0.5	1	0.5ml every 24 hours	1ml every 24 hours
0.9-1.2	0.75	1.5	0.5ml every 12 hours	1ml every 12 hours
1.2-1.5	1	2	0.5ml every 8 hours	1ml every 8 hours
1.5-1.8	1.25	2.5	0.5ml every 6 hours	1ml every 6 hours
1.8-2	1.5	3	1ml every 12 hours	2ml every 12 hours

ii. Moderate Risk Infants (based on increases on 20-30ml/kg/day)

Weight (kg)	Start at (hourly)	Start at (2 hourly)	Increase hourly feed volume by:*	Increase 2hourl feed volume by:
1.0-1.2	1	2	0.5ml every 6 hours	1ml every 6 hours
1.2-1.6	1.5	3	1ml every 12 hours	2ml every 12 hours
1.6-2.0	2	4	1ml every 8 hours	2ml every 8 hours
2-2.4	2.5	5	1ml every 6 hours	2ml every 6 hours
2.4 and above	3	6	1.5ml every 8 hours	3ml every 8 hours

*Note that this refers to the actual feed **volume** based on 1 hourly feeds. Therefore if baby is 2 hourly fed then multiply the amount on this table by 2 to give the increase on the feed volume, if on 3 hourly feeds multiply by 3 and so on.

For peer review only - http://bmjopen.bmj.com/site/about/guidelines.xhtml

University Hospital Southampton NHS

NHS Foundation Trust

Fluid Name / Nutrient	Preterm Stock PN	Preterm + Sodium Stock PN	Term Stock PN	Stock Lipid	Dextrose 10%	MBM/DBM	MBM with Full Fortifier*	Neocate LCP	Peptijunior	LBW Formula (Aptamil Preterm)	Post D/C Formula (Nutriprem 2)	Term formula	Infantrini
**Energy (kcal)**	63.0	59.8	70.2	166.7	40.0	69.0	85.0	71.0	66.0	80.0	75.0	66.0	100.0
**Protein (g)**	2.3	2.8	2.5	0	0.0	1.3	2.5	2.0	1.8	2.6	2.0	1.3	2.6
**Carbohydrate (g)**	12.1	11.0	13.5	0	0.0	7.2	10.0	8.1	6.8	8.4	7.4	7.3	10.3
**Fat (g)**	0	0	0	16.7	0.0	4.1	4.1	3.5	3.5	3.9	4.0	3.5	5.4
**Sodium(mmol)**	0.0	4.3	2.8	0.1	0.0	0.7	2.2	0.8	0.9	3.0	1.2	0.7	1.1
**Potassium (mmol)**	2.4	1.7	1.9	0	0.0	1.5	2.1	1.6	1.7	2.1	2.0	1.6	2.4
**Calcium(mmol)**	0.8	1.0	0.9	0	0.0	0.8	2.5	1.2	1.2	2.3	2.2	1.2	2.0
**Phosphorous (mmol)**	1.0	2.2	0.9	1.5	0.0	0.5	1.7	1.1	0.9	2.0	1.5	0.9	1.3
**Iron (umol)**	0.0	0.0	0.0	0.0	0.0	1.3	1.3	18.8	13.8	25.1	17.9	9.5	21.5
**Vitamin A (IU)**	0.0	0.0	0.0	3910.0	0.0	213.0	985.6	264.0	173.2	599.4	269.7	183.2	333.0
**Vitamin D (IU)**	0.0	0.0	0.0	680.0	0.0	0.0	200.0	51.0	52.0	120.0	68.0	48.0	68.0
**Volume (ml/kg) required to reach recommended protein intake (ELBW infants)**	152	125	140	Contains no protein	Contains no protein	292	152	195	211	146	190	292	146

b. Nutrient Content of Commonly Used Products per 100ml

Typical Values are used and are correct at 18/10/2011

*Based on Cow and Gate Nutriprem Breast Milk Fortifier

For peer review only - http://bmjopen.bmj.com/site/about/guidelines.xhtml

9. SUPPORTING INFORMATION

GUIDELINES AND NUTRITIONAL CARE

There is good evidence from large epidemiological studies such as EPICure that preterm infants often fail to grow adequately, dropping to significantly lower centiles for weight and head circumference at discharge than those which they were born on[9, 10]. There is also evidence that growth failure is also associated with poorer neurodevelopmental outcomes[11]. One significant causative factor for this failure of growth is that these infants receive inadequate nutrition, and there is evidence that they fail to achieve appropriate targets for nutrient intake[12, 13]. Feeding practices across different neonatal units has been shown to be one of the factors responsible for the variability in lengths of stay and the level of postnatal growth restriction seen between different units offering the same level of care[14]. Although there is uncertainty around the definitive practice of nutritional support in preterm infants, there is evidence that standardisation of practice and the use of guidelines is beneficial. A systematic review and meta-analysis by Patole and De Clerk in 2005 showed that the use of standardised feeding regimens reduced rates of NEC, and in the context of the Vermont Oxford Network's 'Potentially Better Practices for Nutrition', the standardisation of practice was shown to reduce the time to start TPN and enteral feeds, improve use of breast milk, reduce lengths of stay and a lower rate of infants being discharges with weights below the 10th centile [4, 15]. Donovan et al studied aspects of nutrient intake and outcomes before and after the introduction of nutrition support guidelines in their NICU, showing significantly earlier initiation of both parenteral and enteral feeding, earlier achievement of full enteral feeding, and earlier regaining of birth-weight after introduction of guidelines[16].

ASSESSMENT AND MONITORING

Some babies are at higher risk than others of nutritional problems – under-nutrition, feed – related complications or both. Regular assessment of nutritional status and monitoring of growth will help identify infants with greater nutritional needs or a higher risk of poor growth or problems. Preterm infants in particular are at risk and should have their weight, head circumference and length measured at a minimum of once a week [4, 6, 17].
The following are things to consider when assessing nutritional risk

- Term babies with appropriate birth weight have good nutrient stores, designed to support them through the first few days when breast milk volumes are low. They are low risk.

Disclaimer: It is your responsibility to check against StaffNet that this printout is the most recent issue of this document

For peer review only - http://bmjopen.bmj.com/site/about/guidelines.xhtml

- Preterm babies have low nutrient stores and are born at time of rapid growth – the earlier they are then the bigger the problem and the greater their nutritional risk. This is compounded by immature gut and metabolic function. They are moderate to high risk (depending on gestation) and need early nutrition support.
- Growth restricted babies have less nutritional reserve; they may also have reduced perfusion to the gut before birth and an increased risk of NEC. These babies will therefore be at greater risk compared to babies of a similar gestation.
- Congenital abnormalities such as gastrointestinal abnormalities, facial anomalies and cardiac problems (including PDA and associated treatment) will all affect nutritional status and increase nutritional risk.
- Acquired disorders such as hypoxic-ischaemic injury, sepsis and NEC will impact on the nutrition infants receive and in turn put them at higher risk of poor nutrition.
- Combinations of the any of the above factors will result in a greater overall risk.

NUTRITIONAL REQUIREMENTS

TERM INFANTS: breast milk provides appropriate nutrients for healthy term babies and breast-feeding should be supported and encouraged. Babies who are not being breast fed should be fed on a standard cows' milk based formula.

PRETERM INFANTS: evidence-based recommendations are available to guide nutrient intakes for preterm infants. The most comprehensive is Tsang 2005 [2], which gives guidelines for parenteral and enteral nutrition support, and specifies requirements for babies <1000g and 1000-1500g birth-weight, during both 'transition' phase (days 2-7 of life) and 'growth phase' (day 7 onwards). ESPHGAN 2010 [1] gives recommendations for enteral intake of fluid and nutrients, though is largely based on the Tsang recommendations. Growth is rapid in the third trimester of fetal life; infants born preterm thus have high requirements for nutrients, but immature physiological capacity to handle them. Breast milk is the optimal first choice for preterm infants' nutrition, however even at high volumes will not provide all adequate nutrients: supplementation with breast milk fortifier or preterm formula may be necessary. The tables in this guideline refer to the Tsang recommendations for energy and protein in VLBW infants and how they compare to typical feeds used in Southampton. Note that only LBW formula milk fed at 150ml/kg/day or fully fortified breast milk fed at 180ml/kg/day is able to achieve the recommended amounts). The full Tsang recommended nutrient intakes are given in Appendix 1. Essentially, the less mature, the lower the nutrient stores/reserves, the earlier nutrient provision is required

STANDARD NUTRITIONAL SUPPORT OF PRETERM AND SICK INFANTS

Disclaimer: It is your responsibility to check against StaffNet that this printout is the most recent issue of this document

For peer review only - http://bmjopen.bmj.com/site/about/guidelines.xhtml

a. PARENTERAL NUTRITION

i. *Early use of PN*

The VON Potentially Better Practices for nutrition state that TPN should be commenced as early as possible, ideally within the first 24 hours of life [4]. This helps prevent the net nutrient loss and catabolism that occurs when an infant is born prematurely. Significant nutritional deficits have been shown to occur in the first few days (up to 2 weeks) after birth, so introduction of TPN early is a strategy to help prevent this [12]. There is also good evidence that it promotes anabolism, prevents the loss of protein mass, improves calorie intakes, can improve growth and is safe [3, 18-21].

ii. *Protein intake*

As described above, nutrient delivery in high risk groups is challenging, and the delivery of protein and energy early in life often fails to meet recommended targets. Whilst intravenous glucose given early on will meet energy needs in many cases, it contains no protein, which can only be administered using TPN or milk feeds. Therefore, in high risk infants who cannot be fully fed quickly, it is vital to give the largest amount of protein possible as TPN, as early as possible to try and prevent the accumulation of deficits. In view of this, Stock TPN in Southampton has recently been reformulated to provide higher levels of protein in a smaller volume. Using high protein TPN to deliver higher protein intakes in the first few days of life in preterm infants has recently been shown to have metabolic benefits in addition to the prevention of catabolism, including a reduction in hyperglycaemia and insulin use [22], and a significant reduction non-oliguric hyperkalaemia [23].

iii. *Peripheral vs central PN*

It is generally accepted that is preferable to given TPN via a percutaneous central venous catheter (‘long line’) than via a peripheral cannula, in view of the decreased risk of extravasation, the difficulty associated in obtaining repeated peripheral access in preterm infants, and the ability to give higher concentrations of glucose and potassium. Central lines on the other hand have the disadvantage of the risk of catheter related infections. A Cochrane review in 2007 concluded that central TPN was not associated with an increased risk of infection compared to peripheral TPN, and there was some evidence that central TPN resulted in a smaller number of catheters/cannulas per infant required to deliver the TPN, together with improved nutrient delivery [24]. However, it also concluded that there was no significant

Disclaimer: It is your responsibility to check against StaffNet that this printout is the most recent issue of this document

For peer review only - http://bmjopen.bmj.com/site/about/guidelines.xhtml

difference in adverse events (including extravasation) when comparing central to peripheral TPN. Therefore, whilst TPN should be given centrally wherever possible, peripheral TPN should be considered in some individual cases where there is significant nutritional risk and a delay or difficulty in obtaining central access [3].

iv. *Monitoring and Complications*

Careful monitoring of patients whilst on TPN is important to ensure appropriate and adequate nutrition, and to identify potential complications, including liver disease, metabolic bone disease and catheter-related infection. Current recommendations regarding monitoring have been laid out by ESPGHANs guidelines on paediatric parenteral nutrition[3]., and can be found in the NNU Parenteral Nutrition Guidebook

b. ENTERAL FEEDING

i. *Choice of milk*

There is good evidence that maternal breast milk (and to some extent donor breast milk) is protective against NEC, so breast milk should be the food of first choice [25-30]. Ideally this should be the mother's own fresh colostrum. All mothers of preterm infants should have lactation support, and help with expressing within 6 hours of birth (ideally within half an hour according to current WHO recommendations)[5]. If no maternal milk available by 48 hours and the baby is ready for milk, consent should be sought to use DBM. However, as DBM is a limited resource and there is evidence it contains fewer nutrients than mother's own breast milk, DBM should be reserved only for the purposes of establishing feeds in high risk infants, as laid out in the DBM guideline). Where breast milk cannot be used, preterm infants should receive a specialist high calorie and high protein formula ('LBW formula')[31-33].Preterm formulas are designed to meet the basic nutritional requirements of most preterm infants when fed between 150 and 180ml/kg. Preterm formulas can be used as soon as commencement of enteral feeding is recommended. Term formulas should not be used as they fail to meet the nutritional needs of premature infants. There is no evidence to support the use of term elemental/semi elemental formulas in the early stages of feeding unless there is a compelling clinical reason to do so.

ii. *Starting Feeds*

The objective of early feeding is to stimulate gut maturation, motility and hormone release. As starvation leads to atrophy of the gut, withholding feeds

Disclaimer: It is your responsibility to check against StaffNet that this printout is the most recent issue of this document

For peer review only - http://bmjopen.bmj.com/site/about/guidelines.xhtml

may render subsequent feeding less safe and protract the time to reach full enteral feeding [34]. No work has yet addressed whether initial feeds should be exclusively breast milk (mother's own or donor) or whether initial feeds should be delayed if only formula is available. However most evidence suggests that any enteral feed given early may be better than gut starvation [35].

Trophic feeding is defined as small volumes of enteral feeds up to 24 mls/kg/day given to promote gut function It has been shown to prevent changes of starvation in gut mucosa, but a systematic review of 9 trials of trophic feeds vs withholding feed, including 754 infants, did not find any difference in overall feed tolerance, weight gain or rates of NEC [36].

Due to concerns about NEC, commencement of enteral feeds is sometimes delayed in preterm infants. A Cochrane review of early vs delayed introduction of progressive enteral feeds did not show an increase in NEC with early feeds, but despite almost 1000 babies in 5 RCTs the conclusion was that data was insufficient [37]. The ADEPT trial randomised 404 preterm, growth-restricted babies to early feeds (start day 2) or late feeds (start day 6): the early group achieved full feeding earlier, required less PN and had less cholestasis, and no difference was seen in incidence of NEC [38]. There is thus no evidence to support delaying feeds; there is a lack of good evidence to guide feeding policy in babies on inotropes and ibuprofen.

iii. *Rate of advancing feeds*

In standard risk infants a rate of increase of 30ml/kg/day is reported safe, whereas data is more limited in the high risk infant. Evidence points towards several days of trophic feeds followed by a rate of increase of 10-20ml/kg/day. There should be a low threshold for withholding stepped increases secondary to tolerance concerns in the high risk infants. There is limited data on this. A Cochrane review [39] including 4 RCTs and 496 babies, considered increase of up to 24 mls/kg/day as slow, and 25 or greater mls/kg/day as rapid. More rapid increase was associated with earlier tolerance of full feeds and faster weight gain, and no difference in NEC, but numbers were too small to make definite conclusions. This topic is being considered by NIHR for a multi-centre UK trial at present.

iv. *Nutritional Supplements:*

As mentioned above, the nutritional needs of preterm infants are greater than infants born at term, and as such breast milk is adequate to meet those

Disclaimer: It is your responsibility to check against StaffNet that this printout is the most recent issue of this document

For peer review only - http://bmjopen.bmj.com/site/about/guidelines.xhtml

needs [2]. In order to maintain the benefits of breast milk whilst optimising the nutritional status and growth of preterm infants' single multicomponent fortifiers (BMF) have been developed.

Concerns with the use of BMFs include tolerance and their effects to increasing osmolality and in turn the risk of NEC. Most studies have found no significant problems with the tolerance of fortified EBM [40], and a recent review of published evidence found no link between the relatively small increases in osmolality caused by the addition of fortifier to breast milk and NEC [41]. A Cochrane review concluded that the use of BMFs can lead to short term improvements in weight, length and head circumference and that while it is unlikely that further comparative studies with breast milk alone are to take place it recommends further research seeks to evaluate long term outcomes of BMF therapy and identify the optimum composition of BMF products [42].

Recommendations made in 2010 by ESPGHAN stated that the feed of choice for preterm infants (<1800g) was mother's own breast milk supplemented with BMF, or special preterm formula if breast milk not available [1].

v. *Nutrition at Discharge:*

Preterm infants are often discharged home with growth below that expected according to their birth centile. A review by ESPGHAN in 2006 looking at the evidence for feeding preterm infants after discharge recommended that infants discharged with an appropriate weight for their corrected gestational age should be discharged either breast feeding (where breast fed) or on regular formula (where formula fed). However, they also concluded that preterm infants discharged with a subnormal weight for their corrected gestation age should receive fortifier in addition to breast milk (where breast fed) or on special high energy/protein preterm infant formula (where formula fed) [43]. Recently, a Cochrane review looked at this in more detail, addressing the question of whether using fortifier in breast fed preterm infants after discharge improved growth. It concluded that using fortifier after discharge improved growth in infancy, though the evidence was limited [44].

Disclaimer: It is your responsibility to check against StaffNet that this printout is the most recent issue of this document

For peer review only - http://bmjopen.bmj.com/site/about/guidelines.xhtml

6. References

1. Agostoni, C., et al., *Enteral nutrient supply for preterm infants: commentary from the European Society of Paediatric Gastroenterology, Hepatology and Nutrition Committee on Nutrition.* J Pediatr Gastroenterol Nutr, 2010. **50**(1): p. 85-91.
2. Tsang, R.C., *Nutrition of the preterm infant*. 2nd ed. ed. 2005, Cincinnati: Digital Educational Publishing. viii, 427 p.
3. Koletzko, B., et al., *1. Guidelines on Paediatric Parenteral Nutrition of the European Society of Paediatric Gastroenterology, Hepatology and Nutrition (ESPGHAN) and the European Society for Clinical Nutrition and Metabolism (ESPEN), Supported by the European Society of Paediatric Research (ESPR).* J Pediatr Gastroenterol Nutr, 2005. **41 Suppl 2**: p. S1-87.
4. Kuzma-O'Reilly, B., et al., *Evaluation, development, and implementation of potentially better practices in neonatal intensive care nutrition.* Pediatrics, 2003. **111**(4 Pt 2): p. e461-70.
5. WHO, *Management and support of infant feeding in maternity facilities*, in *Infant and young child feeding : model chapter for textbooks for medical students and allied health professionals.* 2009, World Health Organisation. p. 29-36.
6. Edmond, K. and R. Bahl, *Optimal feeding of low-birth-weight infants*. 2006, World Health Organisation.
7. Hartnoll, G., P. Betremieux, and N. Modi, *Randomised controlled trial of postnatal sodium supplementation on body composition in 25 to 30 week gestational age infants.* Arch Dis Child Fetal Neonatal Ed, 2000. **82**(1): p. F24-8.
8. Vandenplas, Y., et al., *Guidelines for the diagnosis and management of cow's milk protein allergy in infants.* Arch Dis Child, 2007. **92**(10): p. 902-8.
9. Wood, N.S., et al., *The EPICure study: growth and associated problems in children born at 25 weeks of gestational age or less.* Archives of Disease in Childhood Fetal & Neonatal Edition, 2003. **88**(6): p. F492-500.
10. Ehrenkranz, R.A., et al., *Longitudinal growth of hospitalized very low birth weight infants.* Pediatrics, 1999. **104**(2 Pt 1): p. 280-9.
11. Ehrenkranz, R.A., et al., *Growth in the neonatal intensive care unit influences neurodevelopmental and growth outcomes of extremely low birth weight infants.* Pediatrics, 2006. **117**(4): p. 1253-61.
12. Embleton, N.E., N. Pang, and R.J. Cooke, *Postnatal malnutrition and growth retardation: an inevitable consequence of current recommendations in preterm infants?* Pediatrics, 2001. **107**(2): p. 270-3.
13. Martin, C.R., et al., *Nutritional practices and growth velocity in the first month of life in extremely premature infants.* Pediatrics, 2009. **124**(2): p. 649-57.
14. Cooke, R.J., S.B. Ainsworth, and A.C. Fenton, *Postnatal growth retardation: a universal problem in preterm infants.* Arch Dis Child Fetal Neonatal Ed, 2004. **89**(5): p. F428-30.
15. Patole, S.K. and N. de Klerk, *Impact of standardised feeding regimens on incidence of neonatal necrotising enterocolitis: a systematic review and meta-analysis of observational studies.* Arch Dis Child Fetal Neonatal Ed, 2005. **90**(2): p. F147-51.
16. Donovan, R., et al., *Outcomes of early nutrition support in extremely low-birth-weight infants.* Nutr Clin Pract, 2006. **21**(4): p. 395-400.
17. *Malnutrition- What nurses working with children and young people need to know and do. An RCN position statement*. 2006, Royal College of Nursing.
18. Anderson, T.L., et al., *A controlled trial of glucose versus glucose and amino acids in premature infants.* J Pediatr, 1979. **94**(6): p. 947-51.
19. Ibrahim, H.M., et al., *Aggressive early total parental nutrition in low-birth-weight infants.* J Perinatol, 2004. **24**(8): p. 482-6.
20. Wilson, D.C., et al., *Randomised controlled trial of an aggressive nutritional regimen in sick very low birthweight infants.* Arch Dis Child Fetal Neonatal Ed, 1997. **77**(1): p. F4-11.

Disclaimer: It is your responsibility to check against StaffNet that this printout is the most recent issue of this document

For peer review only - http://bmjopen.bmj.com/site/about/guidelines.xhtml

University Hospital Southampton NHS
NHS Foundation Trust

21. Van Goudoever, J.B., et al., *Immediate commencement of amino acid supplementation in preterm infants: effect on serum amino acid concentrations and protein kinetics on the first day of life.* J Pediatr, 1995. **127**(3): p. 458-65.
22. Mahaveer, A., C. Grime, and C. Morgan, *Increasing early protein intake is associated with a reduction in insulin-treated hyperglycaemia in very preterm infants.* Arch Dis Child Fetal Neonatal Ed, 2011. **96**(Suppl 1): p. Fa21.
23. Iacobelli, S., et al., *Standardized parenteral nutrition in preterm infants: early impact on fluid and electrolyte balance.* Neonatology, 2010. **98**(1): p. 84-90.
24. Ainsworth, S.B., L. Clerihew, and W. McGuire, *Percutaneous central venous catheters versus peripheral cannulae for delivery of parenteral nutrition in neonates.* Cochrane Database Syst Rev, 2007(3): p. CD004219.
25. Lucas, A. and T.J. Cole, *Breast milk and neonatal necrotising enterocolitis.* Lancet, 1990. **336**(8730): p. 1519-23.
26. McGuire, W. and M.Y. Anthony, *Donor human milk versus formula for preventing necrotising enterocolitis in preterm infants: systematic review.* Arch Dis Child Fetal Neonatal Ed, 2003. **88**(1): p. F11-4.
27. Schanler, R.J., et al., *Randomized trial of donor human milk versus preterm formula as substitutes for mothers' own milk in the feeding of extremely premature infants.* Pediatrics, 2005. **116**(2): p. 400-6.
28. Boyd, C.A., M.A. Quigley, and P. Brocklehurst, *Donor breast milk versus infant formula for preterm infants: systematic review and meta-analysis.* Arch Dis Child Fetal Neonatal Ed, 2007. **92**(3): p. F169-75.
29. Schanler, R.J., *Suitability of human milk for the low-birthweight infant.* Clin Perinatol, 1995. **22**(1): p. 207-22.
30. Henderson, G., M.Y. Anthony, and W. McGuire, *Formula milk versus maternal breast milk for feeding preterm or low birth weight infants.* Cochrane Database Syst Rev, 2007(4): p. CD002972.
31. Premji, S.S., T.R. Fenton, and R.S. Sauve, *Higher versus lower protein intake in formula-fed low birth weight infants.* Cochrane Database Syst Rev, 2006(1): p. CD003959.
32. Atkinson, S.A., et al., *Randomized Trial of Feeding Nutrient-Enriched vs Standard Formula to Premature Infants during the First Year of Life.* Pediatric Research. **45**(4): p. 276A.
33. Lucas, A., F. King, and N.B. Bishop, *Postdischarge formula consumption in infants born preterm.* Arch Dis Child, 1992. **67**(6): p. 691-2.
34. Ziegler, E.E., P.J. Thureen, and S.J. Carlson, *Aggressive nutrition of the very low birthweight infant.* Clin Perinatol, 2002. **29**(2): p. 225-44.
35. King, C., *What's new in enterally feeding the preterm infant?* Arch Dis Child Fetal Neonatal Ed, 2010. **95**(4): p. F304-8.
36. Bombell, S. and W. McGuire, *Early trophic feeding for very low birth weight infants.* Cochrane Database Syst Rev, 2009(3): p. CD000504.
37. Morgan, J., L. Young, and W. McGuire, *Delayed introduction of progressive enteral feeds to prevent necrotising enterocolitis in very low birth weight infants.* Cochrane Database Syst Rev, 2011(3): p. CD001970.
38. Leaf, A., et al., *Early or late enteral feeding for preterm growth-restricted infants? The abnormal Doppler enteral prescription trial.* Archives of Disease in Childhood, 2010. **95**: p. A3.
39. Morgan, J., L. Young, and W. McGuire, *Slow advancement of enteral feed volumes to prevent necrotising enterocolitis in very low birth weight infants.* Cochrane Database Syst Rev, 2011(3): p. CD001241.
40. Lucas, A., et al., *Randomized outcome trial of human milk fortification and developmental outcome in preterm infants.* Am J Clin Nutr, 1996. **64**(2): p. 142-51.
41. Pearson, F., M.J. Johnson, and A.A. Leaf, *Milk Osmolality - Does it Matter?* Arch Dis Child Fetal Neonatal Ed, 2011. **In press**.
42. Kuschel, C.A. and J.E. Harding, *Multicomponent fortified human milk for promoting growth in preterm infants.* Cochrane Database Syst Rev, 2004(1): p. CD000343.

Disclaimer: It is your responsibility to check against StaffNet that this printout is the most recent issue of this document

For peer review only - http://bmjopen.bmj.com/site/about/guidelines.xhtml

43. Aggett, P.J., et al., *Feeding preterm infants after hospital discharge: a commentary by the ESPGHAN Committee on Nutrition.* J Pediatr Gastroenterol Nutr, 2006. **42**(5): p. 596-603.
44. McCormick, F.M., et al., *Multinutrient fortification of human breast milk for preterm infants following hospital discharge.* Cochrane Database Syst Rev, 2010(7): p. CD004866.

For peer review only

For peer review only - http://bmjopen.bmj.com/site/about/guidelines.xhtml

University Hospital Southampton NHS
NHS Foundation Trust

Appendix 1- Nutritional requirements of Preterm Infants

	Extremely Low Birth Weight (<1000g)												Very Low Birth Weight (<1500g)											
	parenteral						enteral						parenteral						enteral					
	Day 0 Lower	Day 0 Upper	Trans Lower	Trans Upper	Grow Lower	Grow Upper	Day 0 Lower	Day 0 Upper	Trans Lower	Trans Upper	Grow Lower	Grow Upper	Day 0 Lower	Day 0 Upper	Trans Lower	Trans Upper	Grow Lower	Grow Upper	Day 0 Lower	Day 0 Upper	Trans Lower	Trans Upper	Grow Lower	Grow Upper
**Energy (kcal)**	40	50	75	85	105	115	50	60	90	100	130	150	40	50	60	70	90	100	50	60	75	90	110	130
**Protein (g)**	2	2	3.5	3.5	3.5	4	2	2	3.5	3.5	3.8	4.4	2	2	3.5	3.5	3.2	3.8	2	2	3.5	3.5	3.4	4.2
**Carbohydrate (g)**	7	7	8	15	13	17	7	7	8	15	9	20	7	7	5	12	9.7	15	7	7	5	12	7	17
**Fat (g)**	1	1	1	3	3	4	1	1	1	3	3.2	8.4	1	1	1	3	3	4	1	1	1	3	5.3	7.2
**Sodium (mmol)**	0	1	2	5	3	5	0	1	2	5	3	5	0	1	2	5	3	5	0	1	2	5	3	5
**Chloride (mmol)**	0	1	2	5	3	7	0	1	2	5	3	7	0	1	2	5	3	7	0	1	2	5	3	7
**Potassium (mmol)**	0	0	0	2	2	3	0	0	0	2	2	3	0	0	0	2	2	3	0	0	0	2	2	3
**Calcium (mmol)**	0.5	15	15	15	15	2	0.8	2.5	2.5	2.5	2.5	5.5	0.5	15	15	15	15	2	0.8	2.5	2.5	2.5	2.5	5.5
**Phosphorous (mmol)**	0	0	15	19	15	19	0.6	19	19	4.5	19	4.5	0	0	15	19	15	19	0.6	19	19	4.5	19	4.5
**Magnesium (mmol)**	0	0	0.2	0.3	0.2	0.3	0.1	0.3	0.3	0.6	0.3	0.6	0	0	0.2	0.3	0.2	0.3	0.1	0.3	0.3	0.6	0.3	0.6
**Iron (umol)**	0	0	0	0	1.8	3.6	0	0	0	0	35.8	71.6	0	0	0	0	1.8	3.6	0	0	0	0	35.8	71.6
**Zinc (umol)**	0	2.3	2.3	2.3	6.1	6.1	0	15.3	6.1	18.3	15.3	45.9	0	2.3	2.3	2.3	6.1	6.1	0	15.3	6.1	18.3	15.3	45.9
**Copper (umol)**	0	0	0	0.3	0.3	0.3	0	0	0	2.4	19	2.4	0	0	0	0.3	0.3	0.3	0	0	0	2.4	19	2.4
**Selenium (nmol)**	0	0	0	16.5	19	57	0	0	0	16.5	16.5	57	0	0	0	16.5	19	57	0	0	0	16.5	16.5	57
**Iodine (nmol)**	0	0	0	8	7.9	7.9	0	0	0	473	79	473	0	0	0	8	7.9	7.9	0	0	0	473	79	473
**Manganese (nmol)**	0	0	0	13.7	18.2	18.2	0	0	0	137	13	137	0	0	0	13.7	18.2	18.2	0	0	0	137	13	137
**Vitamin A (IU)**	700	1500	700	1500	700	1500	700	1500	700	1500	700	1500	700	1500	700	1500	700	1500	700	1500	700	1500	700	1500
**Vitamin D (IU)**	40	160	40	160	40	160	150	400	150	400	150	400	40	160	40	160	40	160	150	400	150	400	150	400
**Vitamin E (IU)**	2.8	3.5	2.8	3.5	2.8	3.5	6	12	6	12	6	12	2.8	3.5	2.8	3.5	2.8	3.5	6	12	6	12	6	12
**Vitamin K (ug)**	0	0	22	22	22	22	0	0	18	22	18	22	0	0	22	22	22	22	0	0	18	22	18	22
**Thiamin (ug)**	200	350	200	350	300	350	180	240	180	240	180	240	200	350	200	350	300	350	180	240	180	240	180	240
**Riboflavin (ug)**	150	200	150	200	150	200	250	360	250	360	250	360	150	200	150	200	150	200	250	360	250	360	250	360
**Vitamin B6 (ug)**	150	200	150	200	150	200	150	210	150	210	150	210	150	200	150	200	150	200	150	210	150	210	150	210
**Folate (ug)**	56	56	56	56	56	56	25	50	25	50	25	50	56	56	56	56	56	56	25	50	25	50	25	50
**Vitamin B12 (ug)**	0.3	0.3	0.3	0.3	0.3	0.3	0.3	0.3	0.3	0.3	0.3	0.3	0.3	0.3	0.3	0.3	0.3	0.3	0.3	0.3	0.3	0.3	0.3	0.3
**Biotin (ug)**	5.8	5.8	5.8	5.8	5.8	5.8	3.6	6	3.6	6	3.6	6	5.8	5.8	5.8	5.8	5.8	5.8	3.6	6	3.6	6	3.6	6
**Pantothenic Acid (mg)**	1.2	1.2	1.2	1.2	1.2	1.2	1.2	1.7	1.2	1.7	1.2	1.7	1.2	1.2	1.2	1.2	1.2	1.2	1.2	1.7	1.2	1.7	1.2	1.7
**Niacin (mg)**	4	6.8	4	6.8	4	4.6	3.6	4.8	3.6	4.8	3.6	4.8	4	6.8	4	6.8	4	4.6	3.6	4.8	3.6	4.8	3.6	4.8
**Vitamin C (mg)**	15	25	15	25	15	25	18	24	18	24	18	24	15	25	15	25	15	25	18	24	18	24	18	24
**Taurine (mg)**	0	3.75	1.88	3.75	1.88	3.75	0	9	4.5	9	4.5	9	0	3.75	1.88	3.75	1.88	3.75	0	9	4.5	9	4.5	9
**Choline (mg)**	0	28	14.4	28	14.4	28	0	28	14.4	28	14.4	28	0	28	14.4	28	14.4	28	0	28	14.4	28	14.4	28
**Carnitine (mg)**	0	2.9	2.9	2.9	2.9	2.9	0	2.9	2.9	2.9	2.9	2.9	0	2.9	2.9	2.9	2.9	2.9	0	2.9	2.9	2.9	2.9	2.9
**Inositol (mg)**	0	54	54	54	54	54	0	54	32	81	32	81	0	54	54	54	54	54	0	54	32	81	32	81

Disclaimer: It is your responsibility to check against StaffNet that this printout is the most recent issue of this document

For peer review only - http://bmjopen.bmj.com/site/about/guidelines.xhtml

Clinical Guidance

University Hospital Southampton NHS
NHS Foundation Trust

Affix Patient Label Here

Neonatal Nutritional Screening Tool

To be completed on admission and weekly (every Monday)

Gestation at birth: **Birth Weight:**

1. Assess Growth

Current Weight:		Current Centile:		Birth Centile:	
Current OFC:		Current Centile:		Birth Centile:	
Current Length:		Current Centile:		Birth Centile:	

2. Determine Risk Category

		Tick
HIGH RISK	*Any one of:*<br>• Preterm <28 weeks at birth<br>• Extremely Low Birth Weight < 1000g<br>• Severe IUGR (weight < 2nd centile and AREDFV) <35 weeks<br>• Infant establishing feeds after episode of NEC or GI perforation<br>• Infants with severe congenital GI malformation: gastroschisis<br>• Perinatal hypoxia / ischaemia with multi-organ dysfunction	
MODERATE RISK	*Any one of:*<br>• Preterm $28\text{-}31^{+6}$ weeks, otherwise well<br>• Very Low Birth Weight 1000 - 1500g<br>• Moderate IUGR (weight < 9th centile and AREDFV) <35 weeks<br>• Baby on inotropes<br>• Baby on indomethacin/ibuprofen<br>• Illness or congenital anomaly which may compromise feeding<br>• Polycythaemia	
LOW RISK	*Any one of:*<br>• Preterm $32\text{-}36^{+6}$ weeks, otherwise well<br>• AREDFV / IUGR ≥35 weeks	
NO RISK	• Well Term Infant ≥37 weeks	

3. Determine the need for nutrition team review

The nutriton team should review any infant meeting the following criteria:

	Tick
• High Risk Infants according to criteria above	
• Not regained birth weight by 2 weeks of age	
• >15% weight loss at any time	
• Weight gain <10g/kg/day from 2 weeks of age onwards	
• Drop through 2 centile lines for weight/HC/length	
• Intake <150ml/kg/day from 2 weeks of age onwards	
• NEC or GI surgery at any time	

Name of person completing assessement: ____________________ Signature: ____________________

If completing a first assessment on admission, please place this form in the plastic wallet in the baby's clear plastic nursing folder, next to the nutrition flow charts

If completing a a weekly asessment, please place this form in the box outside Room 3 once filled out

Disclaimer: It is your responsibility to check against StaffNet that this printout is the most recent issue of this document

For peer review only - http://bmjopen.bmj.com/site/about/guidelines.xhtml

Nutrition Team Review

Date: ______

Day: ______

Gestation at Birth: ______

Corrected Gestation: ______

Staff Present:

Current Clinical Issues:

Fluid Intake

Total Prescribed Fluids: ml/kg/day

Enteral Feed Type: Parenteral Feed Type:

Nutrient Intake

Enteral Feed Provides:

Milk Feeds:	ml/kg/day	kcal/kg/day	g/kg/day Protein
**Parenteral Feed Provides:**			
Aqueous PN:	ml/kg/day	kcal/kg/day	g/kg/day Protein
Lipid:	ml/kg/day	kcal/kg/day	
**Total Intake:**	ml/kg/day	kcal/kg/day	g/kg/day Protein
			g/kg/day Lipid

Comments on intake:

Bloods

Hb:	Sodium:	Creatinine:	ALP:
CRP:	Potassium:	Albumin:	ALT:
Other:	Urea:	Bili:	Magnesium:
	Calcium (corr):		Phosphate:

Assessment

Recommendations

Disclaimer: It is your responsibility to check against StaffNet that this printout is the most recent issue of this document

For peer review only - http://bmjopen.bmj.com/site/about/guidelines.xhtml

BMJ Open

Successfully implementing and embedding guidelines to improve the nutrition and growth of preterm infants in neonatal intensive care: A prospective interventional study

Journal:	*BMJ Open*
Manuscript ID	bmjopen-2017-017727.R1
Article Type:	Research
Date Submitted by the Author:	07-Aug-2017
Complete List of Authors:	Johnson, Mark; University Hospital Southampton NHS Foundation Trust and University of Southampton, National Institute for Health Research Southampton Biomedical Research Centre; University Hospital Southampton NHS Foundation Trust, Department of Neonatal Medicine<br>Leaf, Alison; University Hospital Southampton NHS Foundation Trust and University of Southampton, National Institute for Health Research Southampton Biomedical Research Centre; University Hospital Southampton NHS Foundation Trust, Department of Neonatal Medicine<br>Pearson, Freya; University Hospital Southampton NHS Foundation Trust, Department of Neonatal Medicine<br>Clark, Howard; University Hospital Southampton NHS Foundation Trust, Department of Neonatal Medicine; University of Southampton, Faculty of Medicine<br>Dimitrov, Borislav; University of Southampton, Primary Care and Population Sciences<br>Pope, Catherine; University of Southampton, Health Sciences<br>May, Carl; University of Southampton, Faculty of Health Sciences
**Primary Subject Heading**:	Health services research
Secondary Subject Heading:	Nutrition and metabolism, Paediatrics
Keywords:	Change management < HEALTH SERVICES ADMINISTRATION & MANAGEMENT, Protocols & guidelines < HEALTH SERVICES ADMINISTRATION & MANAGEMENT, Neonatal intensive & critical care < INTENSIVE & CRITICAL CARE, NEONATOLOGY, NUTRITION & DIETETICS

SCHOLARONE™
Manuscripts

Successfully implementing and embedding guidelines to improve the nutrition and growth of preterm infants in neonatal intensive care: A prospective interventional study

Mark J Johnson[1,2](m.johnson@soton.ac.uk), Alison A Leaf[1,2](a.a.leaf@soton.ac.uk), Freya Pearson[2](freya.pearson@uhs.nhs.uk), Howard W. Clark[2,3](h.w.clark@soton.ac.uk) Borislav D. Dimitrov[4], Catherine Pope[5] (c.j.pope@soton.ac.uk) & Carl R. May[5] (c.r.may@soton.ac.uk)

[1]National Institute for Health Research, Southampton Biomedical Research Centre, University Hospital Southampton NHS Foundation Trust and University of Southampton, Southampton, UK

[2]Department of Neonatal Medicine, Princess Anne Hospital, University Hospital Southampton NHS Foundation Trust, Southampton, UK

[3]University Child Health, Faculty of Medicine, University of Southampton, Southampton, Hampshire, UK

[4]Primary Care and Population Sciences, Faculty of Medicine, University of Southampton, Southampton, Hampshire, UK

[5]Faculty of Health Sciences, University of Southampton, Southampton, Hampshire, UK

Corresponding Author

Mark J. Johnson
Department of Neonatal Medicine
Mailpoint 105 Level E
University Hospital Southampton NHS Foundation Trust
Princess Anne Hospital
Coxford Road
Southampton
Hants
SO16 5YA
Tel: +442381204643
Email: m.johnson@soton.ac.uk

Keywords: Implementation, Premature Infant, Nutrition, Nutritional Assessment, Growth

Word Count: 4256

For peer review only

For peer review only - http://bmjopen.bmj.com/site/about/guidelines.xhtml

ABSTRACT

Objectives

We aimed to improve the nutritional care of preterm infants by developing a complex (multifaceted) intervention intended to translate current evidence into practice. We used the sociological framework of Normalization Process Theory (NPT), to guide implementation in order to embed the new practices into routine care,

Design

A prospective interventional study with a before and after methodology

Participants

Infants <30 weeks gestation or <1500g at birth.

Setting

Tertiary neonatal intensive care unit

Interventions

The intervention was introduced in phases: Phase 1 (Control period, Jan-Aug 2011); Phase 2 (Partial Implementation; improved parenteral and enteral nutrition solutions, nutrition team, education, Aug–Dec 2011); Phase 3 (Full implementation; guidelines, screening tool, 'nurse champions', Jan-Dec 2012); Phase 4 (Post implementation; Jan-Jun 2013). Bi-monthly audits and staff NPT questionnaires were used to measure guideline compliance and 'normalisation' respectively. NPT scores were used to guide implementation in real time. Data on nutrient intakes and growth were collected continuously.

Results

There were 52, 36, 75 and 35 infants in phases 1, 2, 3 and 4 respectively. Mean guideline compliance exceeded 75% throughout the intervention period, peaking at 85%. Guideline compliance and NPT scores both increased over time, ($r=0.92$ and 0.15, $p<0.03$ for both), with a significant linear association between the two ($r=0.21$, $p<0.01$). There were significant improvements in daily protein intake and weight gain between birth and discharge in phases 2 and 3 compared to phase 1 ($p<0.01$ for all), which were sustained into phase 4.

Conclusions

NPT and audit results suggest that the intervention was rapidly incorporated into practice, with high guideline compliance and accompanying improvements in protein intake and weight gain. NPT appears to offer an effective way of implementing new practices such that they lead to sustained changes in care. Complex interventions based on current evidence can improve both practice and clinical outcomes.

For peer review only

For peer review only - http://bmjopen.bmj.com/site/about/guidelines.xhtml

ARTICLE SUMMARY

Strengths and Limitations of the this study

- This study was novel in using a sociological theory (Normalisation Process Theory) to both guide and measure the process of implementation
- This study shows that complex interventions, when properly implemented, can change practice in a sustained fashion
- The before and after methodology used in this study is a limitation and means result should be interpreted with caution, but allowed the implementation process to be studied more closely and in 'real world' conditions.

For peer review only

For peer review only - http://bmjopen.bmj.com/site/about/guidelines.xhtml

MAIN MANUSCRIPT TEXT

BACKGROUND

Attempts to span translational gaps and implement evidence-based practice into routine clinical practice often fail [1, 2]. This can mean that patients fail to receive optimal treatment, or conversely may mean they receive unnecessary or potentially harmful care. Neonatal intensive care offers important opportunities for professional behaviour change and practice implementation but is a complex and demanding environment. The Neonatal Intensive Care Unit (NICU) has very vulnerable patients with complex and multiple medical problems, and a large multidisciplinary healthcare team working variable shift patterns. It is also a highly technological and information rich environment. Staff must manage and assimilate a constantly changing array of clinical information from a variety of sources, including monitoring equipment and computerised results systems. It is an interaction rich environment too: with complex interactions between different professionals, parents and patients themselves. It is a demanding environment to work in, with priorities constantly changing across the unit as new patients are admitted or others become clinically unstable.

The nutritional care and growth of preterm infants managed in the NICU is an important example of the problem of translating evidence into practice. Recommendations for nutrient intakes have been published [3, 4], however there is evidence that these are not effectively integrated into clinical practice [5]. There is also evidence that inconsistent and variable nutritional care may be partly responsible for sub-optimal growth. Neonatal units offering the same level of care have reported significant variations in rates of postnatal growth restriction and in length of stay, with differences in feeding practices shown to be one of the factors responsible for this variation [6]. Taking this together with the complexity of the NICU environment, it is understandable that current evidence and recommendations for practice fail to be consistently assimilated. We have recently discussed the issues surrounding context and complexity,

For peer review only - http://bmjopen.bmj.com/site/about/guidelines.xhtml

For peer review only

and it is clear that context has a profound effect on the extent to which new practices can be successfully implemented [7].

In this paper we describe the successful implementation of a nutrition guideline for preterm infants in a UK NICU leading to sustained change in practice. We show how integrating this guideline into patient care effectively required a carefully designed programme of translational work that facilitated both professional behaviour change (when professionals work differently) and practice implementation (when they embed new ways of conceptualizing, enacting and organizing practice into their workflow). We explain the operation of this programme of translational work using Normalization Process Theory (NPT) [8, 9], a conceptual tool-kit that helped us both to plan guideline implementation and to understand its dynamics [10]. More than 250 studies have now been reported that employ NPT. It offers a rigorous and transferable explanatory model of the mechanisms that promote implementation processes and fits well with the MRC Framework for Evaluating Complex Interventions [11, 12]. NPT has four main constructs; Coherence (whether people understand the need for change), Cognitive Participation (whether people understand the change itself and what they need to do to enact new practices), Collective action (whether people actually do the work needed for the new practices) and Reflexive monitoring (whether people see the benefit of the new practices in their daily work). In **Figure 1**, we show how the mechanisms that drive implementation processes are characterised in NPT. Whilst NPT provides a robust model of implementation that has often been used retrospectively to explain these process, it has less frequently been used to develop, guide and drive implementation prospectively as it was in the present study.

METHODS

For peer review only

Aims. We hypothesized that (i) the implementation of an evidence-based nutrition guideline for preterm infants would improve nutrient intakes and growth; and (ii) that the use of NPT to monitor and guide implementation of the guideline would result in its successful integration into practice. We anticipated that improvements in nutrient intake and growth that would follow from successful implementation would have important health benefits.

Setting and sample. The study was conducted in a NICU in the South of England. Inborn infants with a gestational age less than 30 weeks or birth weight less than 1501g were eligible for inclusion in the study, and were automatically included from birth to receive the newly implemented service for the provision and monitoring of nutrition for preterm infants. Staff were eligible for inclusion in the study if they were qualified clinicians (nurses, doctors, dietitians) rostered to NICU during the phase 2, 3 and 4 of the implementation study. They took part in individual structured (questionnaire) data collection using an online tool, and semi-structured (qualitative) interviews and focus groups facilitated by MJJ. The study was approved by an NHS Research Ethics Committee, ('Oxford 'B'' Reference 11/sc/0365). **Figure 2** shows a flow chart of the study.

Intervention development. A complex intervention was developed with the aim of translating evidence about the nutritional care of preterm infants into practice. It was based on current literature and practice recommendations available at the time (see additional file 1). To improve the likelihood of implementation and embedding in practice, each component of the intervention also aimed to target implementation mechanisms identified by NPT[13]. The implementation intervention had seven major components:

- A comprehensive nutrition guideline (see additional file 1).
- A screening tool to identify nutritional risk, linked to specific guideline pathways and nutrition review [14].

For peer review only - http://bmjopen.bmj.com/site/about/guidelines.xhtml

- Improved nutritional products: Stock PN solutions were revised to provide more nutrition in a smaller volume and new formula milks and breast milk fortifier introduced with higher nutritional content.
- A multidisciplinary nutrition support team, (consultant neonatologist with an interest in nutrition, a neonatal dietitian, a neonatal pharmacist and nurse champions).
- Nurse champions seconded one day in five to the nutrition team to improve their knowledge and skills nutritional care, and four days in five working clinically, supporting their colleagues in the new ways of working[15].
- A weekly nutrition ward round to review infants at the highest nutritional risk and provide additional management plans for nutrition

Once developed, the clinical guidelines were circulated to staff and two focus groups held in order to both raise awareness of the changes in practice and to gain insight into potential barriers or facilitators to the implementation process, enabling tailoring of the guidelines to the local setting.

Guideline implementation. This was an observational study. Data were collected in discrete periods between January 2011 and June 2013:

a. ***Control period*** (1st January 2011 and 31st July 2011). Nutrient intake and growth data on infants born during this period were collected retrospectively after the study had finished in order to provide a contemporaneous 'control' group.

b. ***Intervention planning and introduction of improved nutrition products*** (August 1st – December 31st 2011). Nutrient intake and growth data on infants were collected prospectively during this period, during which some elements of the intervention (including improved nutritional solutions) were introduced, and staff were consulted about guideline intervention and its associated changes

For peer review only - http://bmjopen.bmj.com/site/about/guidelines.xhtml

in organization and practice. In addition, the work with staff carried out during this period to develop the intervention would also be likely to begin to affect practice.

c. ***Facilitated guideline implementation*** (January 1st- December 31st 2012) during which the full complex intervention was implemented. Nutrient intake and growth data on infants were collected prospectively and audits of guideline compliance and staff NPT Toolkit questionnaires were carried out bi-monthly.

d. ***Post-implementation phase*** (January 1st- June 30th 2013). Nutrient intake and growth data on infants were collected prospectively during this period, and one final audit of guideline compliance was carried out to assess the degree to which the new practices remained in place after the main intervention period.

Patient outcomes. Infant outcomes of primary interest were (i) differences in mean daily energy and protein intakes during stay on NICU between pre-implementation and intervention periods, and (ii) differences in the change in weight and head circumference standard deviation scores (SDS) between birth and discharge. These data were collected by entering infant chart data on fluid and feed intake into a specially designed spreadsheet, which was pre-programmed with the nutrient content of feeds and fluids available on the NICU, and automatically calculated daily energy and protein intakes for each infant. Intakes of energy and protein were calculated as raw values but also as a percentages of the Recommended Range of Intake (RRI) according to Tsang et al 2005, which were the recommendations for the nutritional intake of preterm infants at that time[3]. Of note, these have since been updated by Koletzko et al in 2014, which recommends a slightly higher range of energy intake (110-30kcal/kg/d compared to Tsang's 110-120kcal/kg/day) and higher range of protein intake (3.5-4.5g/kg/day compared to Tsang's 3.0-3.6g/kg/d)[16]. Growth data were collected in a similar manner and converted to SDS using the LMS growth add in for Microsoft Excel using reference data from the UK-WHO Newborn Infant

For peer review only

For peer review only - http://bmjopen.bmj.com/site/about/guidelines.xhtml

192 Close Monitoring growth chart. Growth was measured as the change in SDS between birth and discharge. Differences in patient outcomes were also detected by monitoring routinely collected data on mortality, morbidity (e.g. necrotising enterocolitis; chronic lung disease; retinopathy of prematurity; severe Intraventricular haemorrhage; late onset sepsis) and length of stay.

Guideline normalization and compliance. Measures of nutritional processes were extracted from patient charts at the time of nutritional data entry: time of starting enteral feeds, time of starting PN, time of starting breast milk fortifier and type of feed at discharge. Audits of compliance with the nutrition guideline were carried out throughout the full implementation period, and again at the end of the post-implementation period [17]. Audits were carried out every two months in the implementation phase, and once in the post-implementation phase. Measures of the normalization of guideline compliance were made using a questionnaire based on the NPT online toolkit (www.normalizationprocess.org). This was adapted to ensure that questions related to implementing and embedding the nutrition guideline in practice. This was made available to staff online using www.freeonlinesurveys.com. Respondents were asked to score their level of agreement with each of the 16 items between one and ten. This provided overall scores for each of the four domains of NPT (sense-making, participation, action and monitoring). Staff completed questionnaires anonymously.

Statistical analysis. Descriptive statistics was used to summarise the demographic and outcome variables. The outcome variables were tested for normality using the Kolmogorov–Smirnov test in order to help determine the nature of the analysis methods used, with $p<0.05$ indicating that the tested variable distribution differed from a normal distribution. For normally distributed continuous variables, the mean and standard deviation were calculated, with the median and interquartile range calculated for other continuous variables. Distribution of categorical variables was presented as frequency and percentage. Comparison of daily nutrient intake and growth data between periods was carried out using

For peer review only - http://bmjopen.bmj.com/site/about/guidelines.xhtml

general linear models with mixed effects. This statistical technique accounts for repeated measures in the same infant, allowing the addition of other potentially confounding variables (sex, gestational age at birth and birth weight) and subsequent adjustment of the model. Post-hoc Tukey's test was used to adjust significance values in view of multiple comparisons. For normally distributed data, a type of general linear model was used, whilst for non-normally distributed data a type of generalized linear model was used in which repeated effects are considered random effects. Missing data were left as missing and not imputed.

Mortality and morbidity data and other dichotomous outcomes were compared across study periods using X^2 tests (or Fishers Exact test where numbers were low). Continuous process outcome measures were compared across study periods using either a two-way ANOVA (for normally distributed data) or the Kruskal-Wallis test (for non-normally distributed data). If significant differences were found then comparisons between pairs of groups were further analysed with post hoc adjustment by Tukey's test (normally distributed data) or multiple Mann-Whitney-U tests (non-normally distributed data).

Guideline compliance audit results and measures of the 'normalisation' of practice (using scores from the online NPT questionnaire) were summarised as mean scores and plotted over time. Multiple linear regression was used to describe the nature of the relationship between mean percentage audit compliance and NPT scores over time. A similar approach was then used to relate mean percentage audit compliance and NPT scores to the primary infant outcome measures. Plots of mean percentage audit compliance and NPT scores were overlaid with plots of energy intakes, protein intakes and the differences in weight and head circumference SDS between birth and discharge over time during the intervention period. The analyses were carried out using Stata IC v12.3 (Stata Corp) and SAS 9.3 (SAS Institute Inc.).

For peer review only

For peer review only - http://bmjopen.bmj.com/site/about/guidelines.xhtml

RESULTS

Measures of Infant Outcomes. **Table 1** summarises the sex, gestational age at birth and birth weight of infants in each study period. CRIB II[18] scores are also shown as in indication of illness severity. CRIB II scores were not available for all infants and the numbers available with CRIB scores are also shown in **Table 2**. There were no significant differences in sex, birth weight or gestational age between groups. There was a significant difference in CRIB II scores between groups (p=0.008), with post hoc pairwise testing using Tukey's method revealing that only group D was significantly different (higher) from all the others. This suggests an increased level of illness severity in group D when interpreting results.

Period	n	Male (%)	Mean Birth weight (SD)	Mean Gestational Age (SD)	Mean CRIB II (SD), n
A. Pre-implementation period (Jan 2011 - Jul 2011	52	23 (44.2)	1.084 (0.270)	29.2 (2.6)	7.0 (3.6), 30
B. Partial implementation period (Aug - Dec 2011)	36	18 (50)	1.029 (0.311)	29.2 (2.9)	6.4 (3.9), 20
C. Main Intervention Period (Jan - Dec 2012)	75	37 (49.3)	0.998 (0.269)	28.7 (3.0)	6.9 (2.5), 44
D. Post-implementation period (Jan - Jun 2013)	35	22 (62.9)	0.924 (0.261)	28.1 (2.8)	9.7 (3.2), 18
p value for difference between groups (ANOVA)		0.392*	0.066	0.290	**0.008**

Table 1: Infant Characteristics in each study group (SD-Standard Deviation) *p value is for Chi^2

For peer review only - http://bmjopen.bmj.com/site/about/guidelines.xhtml

Outcome	Mean nutritional process audit compliance	
	Model with Time Excluded	Model with Time Included
**Mean NPT Score Coefficient (p value)**	0.95 (0.002)	0.40 (0.031)
**Time coefficient (p value)**	Omitted	0.72 (<0.0001)
**p value for model**	0.0018	<0.0001
**r for model**	0.2098	0.8076
**r^2 for model**	0.044	0.6522

Table 2: Results of linear regression for mean audit compliance measures and mean NPT scores over time.

Nutrient Intakes over time. When compared with baseline data, progressive increases in protein intake were observed over the course of the study. **Figures 3a-d** show the results of the generalised linear modelling analysis for median daily nutrient intakes for each of energy (kcal/kg/day), protein (g/kg/day), energy (as a percentage of RRI) and protein (as a percentage of RRI) respectively, and data tables showing the intake and differences between periods are given in additional file 2. Using Tukey's test to compare the difference between each period, there were significant improvements in protein intake in period B and C compared to period A (both p<0.001), and this was sustained beyond the intervention into period D (p<0.01 vs periods A and B). Although there was no significant difference between the partial intervention period (B) and the main intervention period (C) in terms of protein intake, there was a significant increase in protein intake between the partial intervention period (B) and the post implementation period (D).

Growth over time The results of the general linear model using mixed effects for the changes in weight and head circumference SDS in each study period are shown in **Figure 4**, and data tables showing the intake and differences between periods are given in additional file 2. Using Tukey's test to compare the difference between each period, there was a significant and sequential improvement in the change in standard deviation score from birth (cSDS) for weight in period B and C compared to period A (both p<0.01), which again were sustained post implementation in period D (p<0.001 vs periods A and B).

For peer review only - http://bmjopen.bmj.com/site/about/guidelines.xhtml

There was also a significant improvement in weight between the partial intervention period (B) and the main intervention period (C), suggesting full implementation further added to the intervention effect. This demonstrates that there was a sequential improvement in the difference in weight SDS between birth and discharge in each period during the study. There was a non-significant improvement in the cSDS for head circumference (HC) across the study.

Mortality and Morbidity. No significant differences were detected in the rates of mortality, chronic lung disease, necrotising enterocolitis, severe intraventricular haemorrhage, retinopathy of prematurity and late onset infection.

Professional behaviour change and practice implementation

Timing of commencement of feeds and types of feed. There were no significant differences in the number of babies receiving breast milk, preterm formula, term formula or mixed feeding at discharge between phases of the study. There were no significant changes in the proportion of breast milk fed infants receiving fortifier, nor were there differences in the time to start enteral feeds or the time of starting fortifier in infants receiving breast milk between study periods. However, there were differences in the median time to starting parenteral nutrition between the phases of the study. In the baseline or control phase of the study this was 15 hrs. Over the pre-implementation and implementation phases of the study this reduced to nine hours. In the post implementation phase this rose to 12 hours. A significant difference between study phases was detected using the Kruskal-Wallis test (p=0.013).

For peer review only - http://bmjopen.bmj.com/site/about/guidelines.xhtml

Adherence to Guideline. Bimonthly guideline compliance audits – described in Figure 2 – during the intervention phase and at the end of the post-implementation phase showed that mean compliance improved incrementally across the implementation phase, but there was a slight decrease in compliance at the final audit in July 2013. Linear regression of mean nutritional audit compliance during the 12 months of the intervention period demonstrated a significant linear increase over time, with a regression coefficient of 1.1 (r=0.92, p=0.009).

Normalisation Process Theory Scores. Taking into account participant dropout due to staff turnover, response rates to the NPT Toolkit questionnaire peaked at 74% in May 2012, falling to 27% in the final questionnaire in July 2013. Details regarding the number and type of respondents can be seen in **table 3**. **Figure 5** shows NPT scores as radar plots for each time period ; in general, the fuller the radar plot, the greater extent to which staff felt that the practices were part of 'normal practice' at that time. Radar plots generally become fuller over time, though some key areas of the plots were less full at different time points, indicating areas for improvement. The items relating to collective action and reflexive monitoring were scoring lower early in the intervention period, indicating that staff could not see the benefit of the intervention in their work. In order to address this, the results of the study to date were displayed around the staff areas of NICU in August 2012, with a subsequent improvement in the related NPT scores. There was a significant linear increase in mean NPT score over time (coefficient=0.031, r=0.15, p=0.023), though this dropped off during the post-implementation phase. **Figure 6** shows that global NPT scores and guideline compliance increased together over time and then flattened out in the post implementation phase. Linear regression analysis showed that there was a significant association between mean global NPT scores and audit compliance through the intervention development, implementation and post-implementation phases of the study with a coefficient of 0.95 (r=0.21,

For peer review only

For peer review only - http://bmjopen.bmj.com/site/about/guidelines.xhtml

p=0.002, see table 2). The addition of time as a variable into the linear regression models (to account for the repeated measures nature of the data) is also shown in table 2. The addition of time significantly contributed to the increases in compliance over the study and increased the predictive value of the model, though despite this the mean NPT scores remained a significant predictor, showing that the measures of normalisation using NPT are associated with measures of clinical practice. Linear regression using the mean individual construct scores for NPT showed a significant association with the mean audit scores and participants' capacity to monitor the effects of their actions (*reflexive monitoring*), both before and after adjustment for the effect of time (coefficients of 0.89 and 0.51, p=0.034 and p=0.044 with and without adjustment for time respectively).

Time Period	Mar-12	May-12	Jul-12	Sep-12	Nov-12	Jan-13	Jul-13
Number of Respondents	44	52	39	26	24	18	16
Percentage Response Rate	57.9	74.3	58.2	41.3	40.7	31	27
Number (%) Consultants	4 (9.1)	4 (7.7)	4 (10.3)	4 (15.4)	4 (16.7)	3 (16.7)	4 (25)
Number (%) Junior Doctors/ANNPs	1 (2.3)	3 (5.8)	3 (7.7)	0 (0)	0 (0)	0 (0)	0 (0)
Number (%) Pharmacists	1 (2.3)	1 (1.9)	1 (2.6)	0 (0)	0 (0)	0 (0)	0 (0)
Number (%) Band 7 Nurses	4 (9.1)	4 (7.7)	2 (5.1)	3 (11.5)	5 (20.8)	2 (11.1)	2 (12.5)
Number (%) Band 6 Nurses	10 (22.7)	9 (17.3)	6 (15.4)	7 (26.9)	6 (25.0)	5 (27.8)	4 (25.0)
Number (%) Band 5 Nurses	19 (43.1)	23 (44.2)	18 (46.2)	10 (38.5)	6 (25.0)	5 (27.8)	4 (25)
Number (%) Band 4 Nurses	2 (4.6)	4 (7.7)	2 (5.1)	1 (3.9)	1 (4.2)	0 (0)	2 (12.5)
Number (%) Band 3 Nurses or lower	3 (6.8)	4 (7.7)	3 (7.7)	1 (3.85)	2 (8.3)	1 (5.6)	1 (6.3)

Table 3: Number of respondents and percentage response rate for each NPT questionnaire

For peer review only - http://bmjopen.bmj.com/site/about/guidelines.xhtml

For peer review only

DISCUSSION

We evaluated the effects of guideline implementation by measuring objective changes in nutrition intake. These data are important in their own right, but can also be used to corroborate subjective self-reports of behaviour change and practice implementation by staff. Objective improvements in nutrient intake and weight gain were detected in infants across the four data collection periods. Against this background, mean audit guideline compliance and NPT scores both increased in a linear fashion over time. Impressively, mean guideline compliance was in excess of 75% throughout the intervention period, peaking at 85%. The headline result of this study is that implementation of the guideline was successfully achieved, and that activities associated with specific intervention components were routinely embedded in workflow within the NICU. This paper has described the successful implementation of a nutrition guideline for preterm infants in NICU, leading to sustained change in practice and improved nutritional outcomes. During the time this study was active, other groups have used similar approaches in the preterm population in order to try and improve infant growth in NICU [19, 20]. They also used before and after study designs, but did not include a process evaluation.

Our study has shown that implementing a facilitated nutrition guideline in NICU using a multifaceted intervention improved protein intake and weight gain in preterm infants. Our process evaluation demonstrates that using NPT to develop and guide the implementation process can lead to high compliance with guidelines and changes in practice that are sustained beyond the initial intervention period. The results also show that measures of normalisation using the NPT toolkit correlate well with measures of clinical practice in real life, and suggest that NPT may therefore offer an effective way of measuring and guiding the implementation process. Effectively implementing the components of this intervention significantly improved both protein intake and weight gain, and appeared to prevent the 'expected' fall of around 1.5-2 SDS for weight between birth and discharge reported in other studies [21,

For peer review only - http://bmjopen.bmj.com/site/about/guidelines.xhtml

22]. This may be clinically relevant; for example, it may lead to improved neurodevelopmental outcomes [23-25] and so follow up of the infants in this study will be important. Improvement in weight gain and protein intake appears to continue into the post implementation period, suggesting that improvements were sustained beyond the main intervention period. It is of interest however, that despite the improvements seen, infants did still fall 0.39 standard deviations for weight between birth and discharge. Whilst such a fall may be considered normal fluctuation around a centile line, it is relevant that even the end of the study infants still only received around 3.34g/kg/day of protein (86.8% of RRI) on average across stay, so were still not receiving recommended amounts of protein. This may explain why they still displayed some negative growth. Suboptimal intake of other nutrients such as electrolytes, vitamins and trace elements may also have contributed. Similarly, this may also have contributed to the lack of significant improvements in head growth, although this may in part have been due to poor collection of head circumference data in the earlier phases of the study (as staff did not begin measuring it consistently until the first intervention period) meaning there were insufficient numbers for a statistically significant result despite a trend towards improvement across the study.

In the present study, audits of guideline compliance were used in combination with the NPT toolkit. The audits measured how well the guideline was put into practice, and the toolkit provided insight into how well the intervention was being integrated into routine care by staff and identified areas where more work was needed to aid implementation. NPT was used prospectively for the first time in this study to develop and drive the intervention, rather than retrospectively assessing the implementation process. In particular, the guidelines were aimed at encouraging *coherence* and *cognitive participation* by being clear about the reasoning behind the approaches used and how to use them. Similarly, the nutrition team, nurse champions and nutrition ward round aimed to provide feedback to aid *reflexive monitoring*. Audit compliance generally improved over the course of the intervention period, and was around 80%,

For peer review only - http://bmjopen.bmj.com/site/about/guidelines.xhtml

which is exceptionally high for studies of implementation. NPT scores generally increased over time, suggesting the intervention was becoming 'normalised' into practice. While the use of the NPT Toolkit to measure normalisation in this study was novel and experimental, it seems that the measure of 'normalisation' provided by the NPT toolkit does relate to practice changes in the 'real world'. Here, subjective self-reports by staff related well to objective measures of guideline compliance. Global NPT scores were high even at the start of the intervention, suggesting that staff felt the intervention became embedded into routine care rapidly. Importantly, in this study, the use of NPT provided a framework to think through the implementation process, with the NPT toolkit measures allowing the implementing team to see where the implementation process could be improved by highlighting how to better engage staff or alter the intervention in areas where NPT scores were low. This unique way of driving, measuring and adjusting the intervention to enhance uptake meant that the use of NPT in this study contributed to the success of the intervention.

A notable result of this study is the importance of reflexive monitoring of implementation progress by staff. This was significantly associated with audit compliance (r = 0.25). However, it accounted for 6% of the variation in audit compliance and it had an effect size of an improvement of 0.9% audit compliance for every point in global NPT scores. Seeing the impact of personal action functions as a feedback mechanism, and such 'feedback loops' are likely to be responsible for the efficacy of professional interventions such as 'audit and feedback' and 'educational outreach' from other health professionals [10]. Both of these were central components of the intervention. These findings are also consistent with those of a previous theory-led overview of systematic reviews of professional interventions using NPT by our group, which showed that those interventions that emphasised *reflexive monitoring* were more likely to be successful [10]. Showing staff the results of the study to date during the main implementation period in response to low *reflexive monitoring* scores demonstrates the utility of NPT to

For peer review only - http://bmjopen.bmj.com/site/about/guidelines.xhtml

identify issues and make implementation a more dynamic process. It also illustrates how addressing such issues results in responsive changes that can be seen in subsequent NPT scores, suggesting that NPT offers a way to both *measure* and *guide* change.

We have previously discussed the importance of context in relation to implementation, suggesting that NPT is also able to provide a lens through which to consider the interactions between context and complex interventions [7]. We proposed that the *plasticity* of interventions and the *elasticity* of the context into which they were introduced played a significant part in the degree of implementation success. Using NPT in the present study to both develop and guide the implementation process, perhaps helped overcome the issues with the complex context of the NICU, providing contemporaneous feedback on the barriers to implementation and allowing a degree of plasticity of the intervention itself. This process was also facilitated by the focus groups prior to implementation, allowing potential barriers to be overcome by alterations in the intervention components and the way in which they were delivered. In addition, the focus groups suggested a desire from staff for more consistency in nutritional care, and this in turn is likely to have improved the elasticity of the host context, facilitating *normative restructuring* around the intervention and aiding implementation. This may explain the high degree of compliance and normalisation seen in the present study.

There were some limitations to this study. As a controlled before and after study, it is not possible to be sure if any of the changes seen during the study are a direct result of the intervention. As this was not a randomised controlled trial, it cannot control for causal mechanisms and confounders, and as such it is subject to limits of interpretation. Whilst the statistical analyses show associations between the progressive implementation of the intervention and changes in outcomes, it cannot prove causation. A further limitation relates to having adequate patient numbers and statistical power to detect important differences, which may possibly account for the failure to detect a clinically significant improvement in

For peer review only - http://bmjopen.bmj.com/site/about/guidelines.xhtml

head circumference. The study was also not powered to detect differences in mortality and morbidity data. An important limitation of the NPT toolkit questionnaires used in this study is that staff responses may have been biased by their beliefs about the expectations of the study team, which is a common problem in such studies. In addition, the specific interventions used in this study required some additional resources (in terms of the nutrition team) and investment by staff, which may not be available in all units. Several studies have used single interventions such as the introduction of a dietitian or guidelines, and shown improvements in nutrient intakes and growth, without the multifaceted and complex process used in this study[26-28]. Whilst such simple approaches may be more straightforward and require less resource, they are dependent on the expertise of the individuals and their ongoing availability. Our approach employing multiple methods and using sociological theory (NPT) to tailor the intervention to the specific context aimed to embed the changes in nutritional practice into routine care. This enabled it to account for locally available resources, and other units could use a similar approach to develop a multifaceted intervention based on their resources and needs.

CONCLUSION

This study used nutrition in the NICU as a vehicle to understand implementation in a complex environment. It has demonstrated that the implementation of the facilitated guideline was associated with improvements in infant protein intakes and weight. The use of NPT to guide and monitor the implementation of the intervention resulted in high guideline compliance and a degree of 'normalisation' of the complex intervention into routine care. Measures of normalisation using NPT appear to relate to objective measures of practice, suggesting that NPT could provide a useful way of understanding the dynamics of implementation processes in complex clinical environments.

For peer review only - http://bmjopen.bmj.com/site/about/guidelines.xhtml

For peer review only

For peer review only - http://bmjopen.bmj.com/site/about/guidelines.xhtml

LIST OF ABBREVIATIONS:

ALP – Alkaline Phosphatase; ESPGHAN - European Society for Pediatric Gastroenterology; Hepatology and Nutrition; NICU - Neonatal Intensive Care Unit; NPT – Normalization Process Theory; PN – Parenteral Nutrition; RNI – Reference Nutrient Intake; RRI – Reasonable Range of Intake; WHO – World Health Organisation.

DECLARATIONS

Ethics approval and consent to participate

The study was approved by an NHS Research Ethics Committee, ('Oxford 'B'' Reference 11/sc/0365)

Consent for publication

Not applicable

Availability of data and material

The datasets generated and/or analysed during the current study are not publicly available due to further pending publications and current approvals, but may be available from the corresponding author on reasonable request. An implementation toolkit and a validated instrument to measure implementation processes using Normalisation Process Theory are available at www.normalizationprocess.org.

Competing interests

For peer review only - http://bmjopen.bmj.com/site/about/guidelines.xhtml

For peer review only

All authors have completed the ICMJE uniform disclosure form at www.icmje.org/coi_disclosure.pdf and declare: no support from any organisation for the submitted work; CRM is an original author of Normalization Process Theory; no other relationships or activities that could appear to have influenced the submitted work.

Funding

Work leading to this paper was funded by the National Institute for Health Research (NIHR) Biomedical Research Centre, Southampton. MJ's contribution was partly supported by an NIHR Doctoral Research Fellowship DRF-2012-05-272, and CRM's contribution was partly supported by NIHR CLAHRC Wessex and partly by ESRC Grant ES-062-23-3274. We gratefully acknowledge the financial support from these agencies. Funders had no role in the design of the study and collection, analysis and interpretation of the data or in writing the manuscript, and the paper does not necessarily represent their views.

Authors' contributions

MJJ contributed to the design of the study, carried out data analysis and interpreted all data. He was responsible for drafting the article and revising it critically for important intellectual content. He is guarantor. AAL, FP, HWC contributed to the conception and design of the study and interpretation of data. They revised the article critically for important intellectual content. BDD supervised the statistical analysis and developed the statistical model used for longitudinal data analysis. He contributed to the interpretation of data and revised the article critically for important intellectual content. CJP and CRM contributed to the design of the study, the use of NPT in the study and interpretation of data. They revised the article critically for important intellectual content.

Acknowledgements

For peer review only - http://bmjopen.bmj.com/site/about/guidelines.xhtml

We would like to further acknowledge the work of Amanda Beedham and Karen Hayllar at Co.Efficient Consultancy, who provided expertise in the development phase and were contracted to develop and build the electronic tool for calculating and storing daily nutrient intake and growth data. We would also like to acknowledge Miss Zoe Lansdowne and Dr Amanda Bevan (Neonatal and Paediatric Pharmacists, University Hospital Southampton NHS Foundation Trust) for their help with the parenteral nutrition solution data used in the electronic tool.

We would also like to acknowledge Jenny Pond, Jane Rhodes-Kitson, Charlotte Oates, Jenny Weddell and, Linda Anderson, Christina Humphrey and Liz Blake for their help in data collection and in their roles as nurse 'Champions for Nutrition'.

Finally, we would like to dedicate this paper to Borislav D. Dimitrov, who sadly passed away during the publication of this work.

FIGURE TITLES AND LEGENDS

Figure 1: The Model of Normalization Process Theory
The four main constructs of NPT are shown in bold. Reproduced with permission [8]

Figure 2: Study process flow chart

Figure 3: Bar graphs showing mean daily nutrient intakes across the four study periods
Bars show mean daily Energy in kcal/kg/day (A), protein in g/kg/day (B), energy as a percentage of RRI (C) and protein as a percentage of RRI (D).Error bars represent 95% confidence intervals. Blue bars represent unadjusted data, while red bars are adjusted for sex, gestational age and weight at birth. *$p<0.05$ for difference vs period A, †$p<0.05$ for difference vs period B, +$p<0.05$ for difference vs period C. (RRI- reasonable range of intake)

Figure 4: Bar graphs showing mean change in standard deviation score (SDS) across the four study periods
Changes are from birth until discharge for weight (A) and head circumference (B). Error bars represent 95% confidence intervals. Blue bars represent unadjusted data, whilst red bars are adjusted for sex, gestational age and weight at birth *$p<0.05$ for difference vs period A, †$p<0.05$ for difference vs period B, +$p<0.05$ for difference vs period C

For peer review only

For peer review only - http://bmjopen.bmj.com/site/about/guidelines.xhtml

Figure 5: Radar plots showing the mean score for each sub-construct of NPT

Results from the NPT questionnaire taken throughout the course of the study.

Figure 6: Relationship over time between mean NPT scores and percentage guideline compliance

ADDITIONAL FILES

Additional File 1.pdf: Nutrition guideline used in this study

Additional File 2.pdf: Data tables to accompany Figure 3 (mean daily nutrient intakes across stay) and Figure 4 (growth over stay)

For peer review only

For peer review only - http://bmjopen.bmj.com/site/about/guidelines.xhtml

REFERENCES

1. Grimshaw JM, Eccles MP, Lavis JN, et al. Knowledge translation of research findings. Implement Sci 2012;**7**:50 doi: 10.1186/1748-5908-7-50

1748-5908-7-50 [pii][published Online First: Epub Date]|.
2. Eccles M, Mittman B. Welcome to Implementation Science. Implementation Science 2006;**1**(1):1 doi: 10.1186/1748-5908-1-1[published Online First: Epub Date]|.
3. Tsang RC. *Nutrition of the preterm infant*. 2nd ed. ed. Cincinnati: Digital Educational Publishing, 2005.
4. Agostoni C, Buonocore G, Carnielli VP, et al. Enteral nutrient supply for preterm infants: commentary from the European Society of Paediatric Gastroenterology, Hepatology and Nutrition Committee on Nutrition. J Pediatr Gastroenterol Nutr 2010;**50**(1):85-91 doi: 10.1097/MPG.0b013e3181adaee0[published Online First: Epub Date]|.
5. Embleton NE, Pang N, Cooke RJ. Postnatal malnutrition and growth retardation: an inevitable consequence of current recommendations in preterm infants? Pediatrics 2001;**107**(2):270-3
6. Cooke RJ, Ainsworth SB, Fenton AC. Postnatal growth retardation: a universal problem in preterm infants. Arch Dis Child Fetal Neonatal Ed 2004;**89**(5):F428-30 doi: 10.1136/adc.2001.004044

89/5/F428 [pii][published Online First: Epub Date]|.
7. May CR, Johnson M, Finch T. Implementation, context and complexity. Implement Sci 2016;**11**(1):141 doi: 10.1186/s13012-016-0506-3

10.1186/s13012-016-0506-3 [pii][published Online First: Epub Date]|.
8. May C, Finch T. Implementing, Embedding, and Integrating Practices: An Outline of Normalization Process Theory. Sociology 2009;**43**(3):535-54 doi: 10.1177/0038038509103208[published Online First: Epub Date]|.
9. May CR, Mair F, Finch T, et al. Development of a theory of implementation and integration: Normalization Process Theory. Implementation Science 2009;**4**(1):29 doi: 10.1186/1748-5908-4-29[published Online First: Epub Date]|.
10. Johnson MJ, May CR. Promoting professional behaviour change in healthcare: what interventions work, and why? A theory-led overview of systematic reviews. BMJ Open 2015;**5**(9):e008592 doi: 10.1136/bmjopen-2015-008592

bmjopen-2015-008592 [pii][published Online First: Epub Date]|.
11. Craig P, Dieppe P, Macintyre S, et al. Developing and Evaluating Complex Interventions: New Guidance: Medical Research Council, 2008.
12. Tabak RG, Khoong EC, Chambers DA, et al. Bridging research and practice: models for dissemination and implementation research. Am J Prev Med 2012;**43**(3):337-50 doi: 10.1016/j.amepre.2012.05.024

S0749-3797(12)00389-3 [pii][published Online First: Epub Date]|.
13. Murray E, Treweek S, Pope C, et al. Normalisation process theory: a framework for developing, evaluating and implementing complex interventions. BMC medicine 2010;**8**:63 doi: 10.1186/1741-7015-8-63[published Online First: Epub Date]|.
14. Johnson MJ, Pearson F, Emm A, et al. Developing a new screening tool for nutritional risk in neonatal intensive care. Acta Paediatr 2015;**104**(2):e90-3 doi: 10.1111/apa.12855[published Online First: Epub Date]|.

For peer review only - http://bmjopen.bmj.com/site/about/guidelines.xhtml

15. Westbury JA, Johnson MJ, Pond JP, et al. Developing the role of the nurse as a link advisor for research and a champion for nutrition in the neonatal intensive care unit. Journal of Neonatal Nursing 2013;**19**:198 doi: http://dx.doi.org/10.1016/j.jnn.2013.01.003[published Online First: Epub Date]|.
16. Koletzko B, Poindexter B, Uauy R. *Nutritional care of preterm infants : scientific basis and practical guidelines*. Basel: S. Karger AG, 2014.
17. Lee L, Girish S, van den Berg E, et al. Random safety audits in the neonatal unit. Arch Dis Child Fetal Neonatal Ed 2009;**94**(2):F116-9 doi: adc.2007.131052 [pii]

10.1136/adc.2007.131052[published Online First: Epub Date]|.
18. Parry G, Tucker J, Tarnow-Mordi W. CRIB II: an update of the clinical risk index for babies score. Lancet 2003;**361**(9371):1789-91 doi: S0140-6736(03)13397-1 [pii]

10.1016/S0140-6736(03)13397-1[published Online First: Epub Date]|.
19. Rochow N, Fusch G, Muhlinghaus A, et al. A nutritional program to improve outcome of very low birth weight infants. Clin Nutr 2012;**31**(1):124-31 doi: 10.1016/j.clnu.2011.07.004

S0261-5614(11)00131-2 [pii][published Online First: Epub Date]|.
20. Roggero P, Gianni ML, Orsi A, et al. Implementation of nutritional strategies decreases postnatal growth restriction in preterm infants. PLoS One 2012;**7**(12):e51166 doi: 10.1371/journal.pone.0051166

PONE-D-12-22771 [pii][published Online First: Epub Date]|.
21. Ehrenkranz RA, Younes N, Lemons JA, et al. Longitudinal growth of hospitalized very low birth weight infants. Pediatrics 1999;**104**(2 Pt 1):280-9
22. Cole TJ, Statnikov Y, Santhakumaran S, et al. Birth weight and longitudinal growth in infants born below 32 weeks' gestation: a UK population study. Arch Dis Child Fetal Neonatal Ed 2013 doi: 10.1136/archdischild-2012-303536

archdischild-2012-303536 [pii][published Online First: Epub Date]|.
23. Ehrenkranz RA, Dusick AM, Vohr BR, et al. Growth in the neonatal intensive care unit influences neurodevelopmental and growth outcomes of extremely low birth weight infants. Pediatrics 2006;**117**(4):1253-61 doi: 117/4/1253 [pii]

10.1542/peds.2005-1368[published Online First: Epub Date]|.
24. Stephens BE, Walden RV, Gargus RA, et al. First-week protein and energy intakes are associated with 18-month developmental outcomes in extremely low birth weight infants. Pediatrics 2009;**123**(5):1337-43 doi: 123/5/1337 [pii]

10.1542/peds.2008-0211[published Online First: Epub Date]|.
25. Chan SH, Johnson MJ, Leaf AA, et al. Nutrition and neurodevelopmental outcomes in preterm infants: a systematic review. Acta Paediatr 2016;**105**(6):587-99 doi: 10.1111/apa.13344[published Online First: Epub Date]|.
26. Donovan R, Puppala B, Angst D, et al. Outcomes of early nutrition support in extremely low-birth-weight infants. Nutr Clin Pract 2006;**21**(4):395-400 doi: 21/4/395 [pii][published Online First: Epub Date]|.
27. Sneve J, Kattelmann K, Ren C, et al. Implementation of a multidisciplinary team that includes a registered dietitian in a neonatal intensive care unit improved nutrition outcomes. Nutr Clin Pract 2008;**23**(6):630-4 doi: 23/6/630 [pii]

For peer review only - http://bmjopen.bmj.com/site/about/guidelines.xhtml

10.1177/0884533608326140[published Online First: Epub Date]|.
28. Olsen IE, Richardson DK, Schmid CH, et al. Dietitian involvement in the neonatal intensive care unit: more is better. J Am Diet Assoc 2005;**105**(8):1224-30 doi: S0002-8223(05)00645-0 [pii] 10.1016/j.jada.2005.05.012[published Online First: Epub Date]|.

For peer review only

For peer review only - http://bmjopen.bmj.com/site/about/guidelines.xhtml

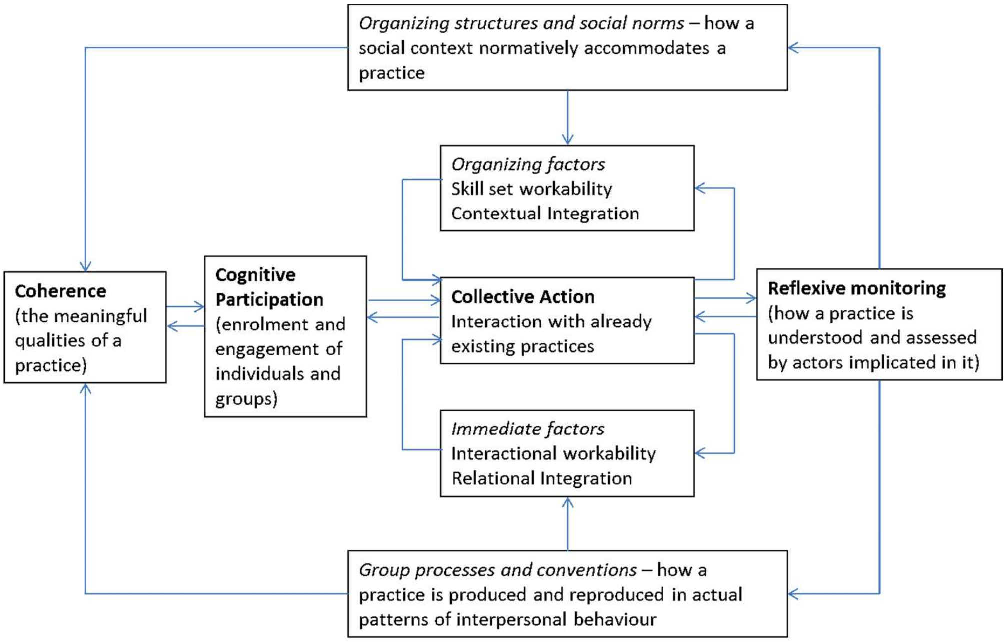

Figure 1: The Model of Normalization Process Theory
The four main constructs of NPT are shown in bold. Reproduced with permission [8]

119x76mm (300 x 300 DPI)

view only

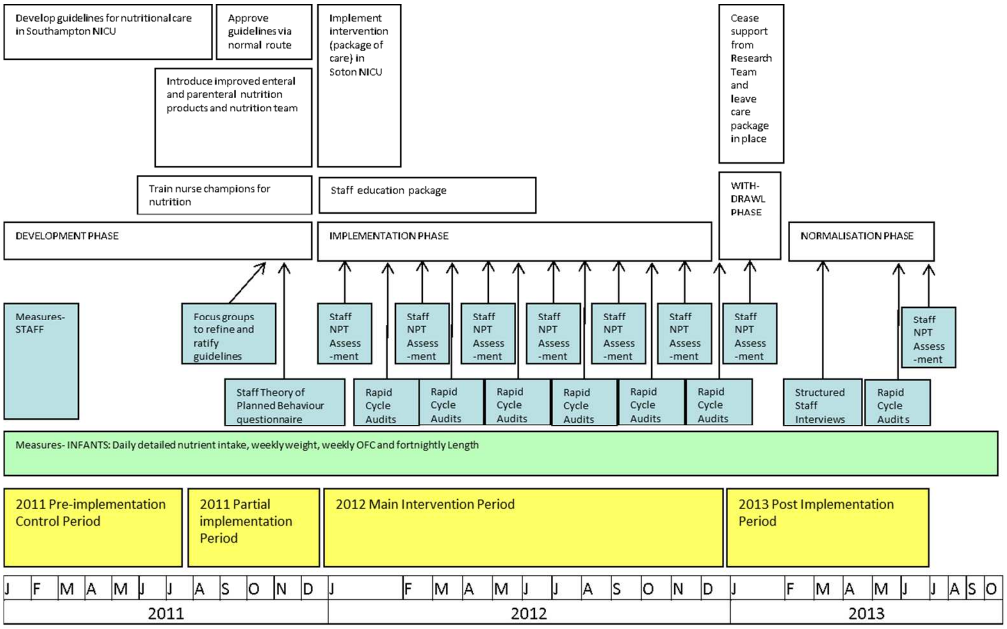

Figure 2: Study process flow chart

110x69mm (300 x 300 DPI)

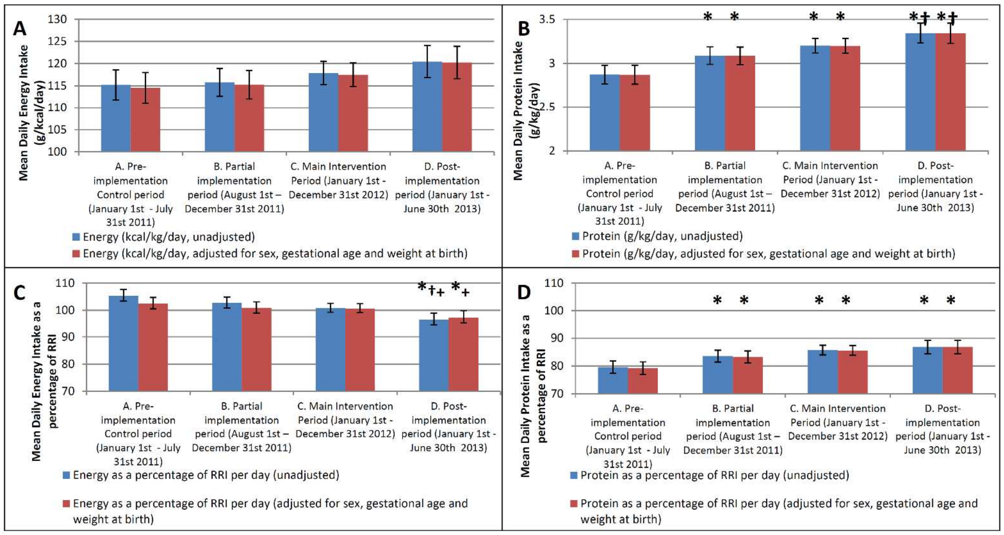

Figure 3: Bar graphs showing mean daily nutrient intakes across the four study periods
Bars show mean daily Energy in kcal/kg/day (A), protein in g/kg/day (B), energy as a percentage of RRI (C) and protein as a percentage of RRI (D).Error bars represent 95% confidence intervals. Blue bars represent unadjusted data, while red bars are adjusted for sex, gestational age and weight at birth. ∗p<0.05 for difference vs period A, †p<0.05 for difference vs period B, +p<0.05 for difference vs period C. (RRI-reasonable range of intake)

370x196mm (300 x 300 DPI)

view only

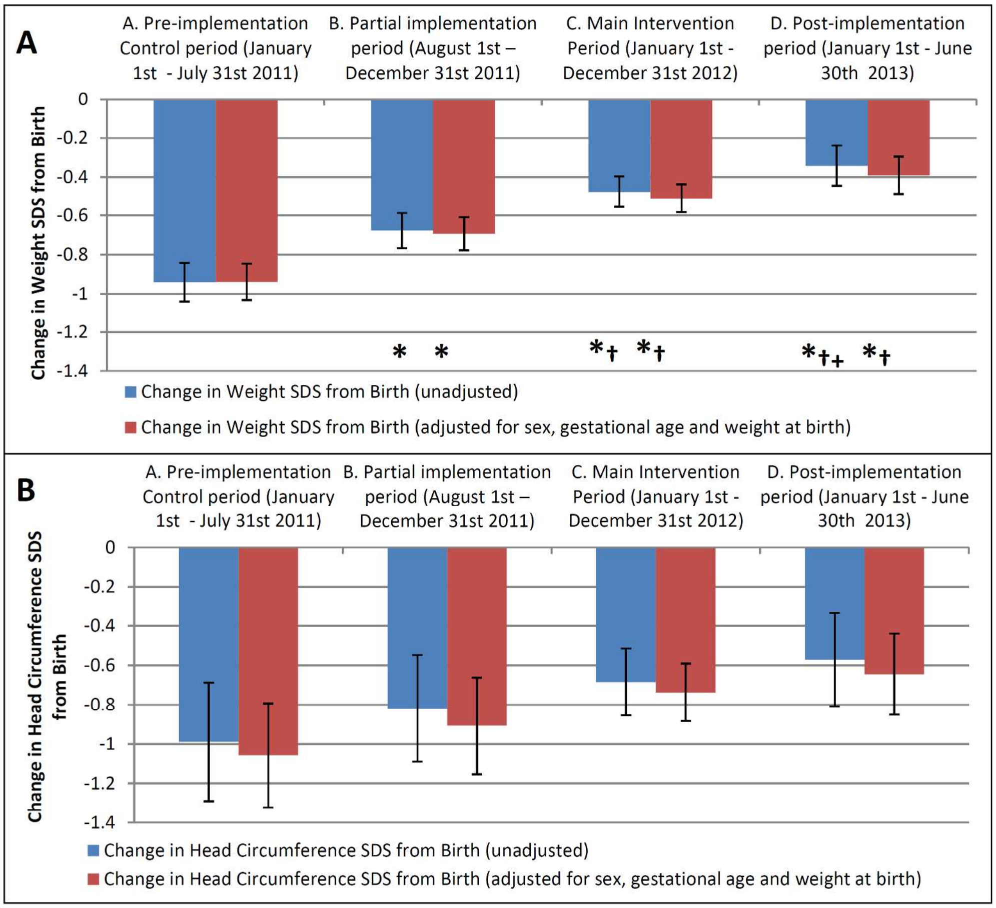

Figure 4: Bar graphs showing mean change in standard deviation score (SDS) across the four study periods Changes are from birth until discharge for weight (A) and head circumference (B). Error bars represent 95% confidence intervals. Blue bars represent unadjusted data, whilst red bars are adjusted for sex, gestational age and weight at birth ∗p<0.05 for difference vs period A, †p<0.05 for difference vs period B, +p<0.05 for difference vs period C

236x215mm (300 x 300 DPI)

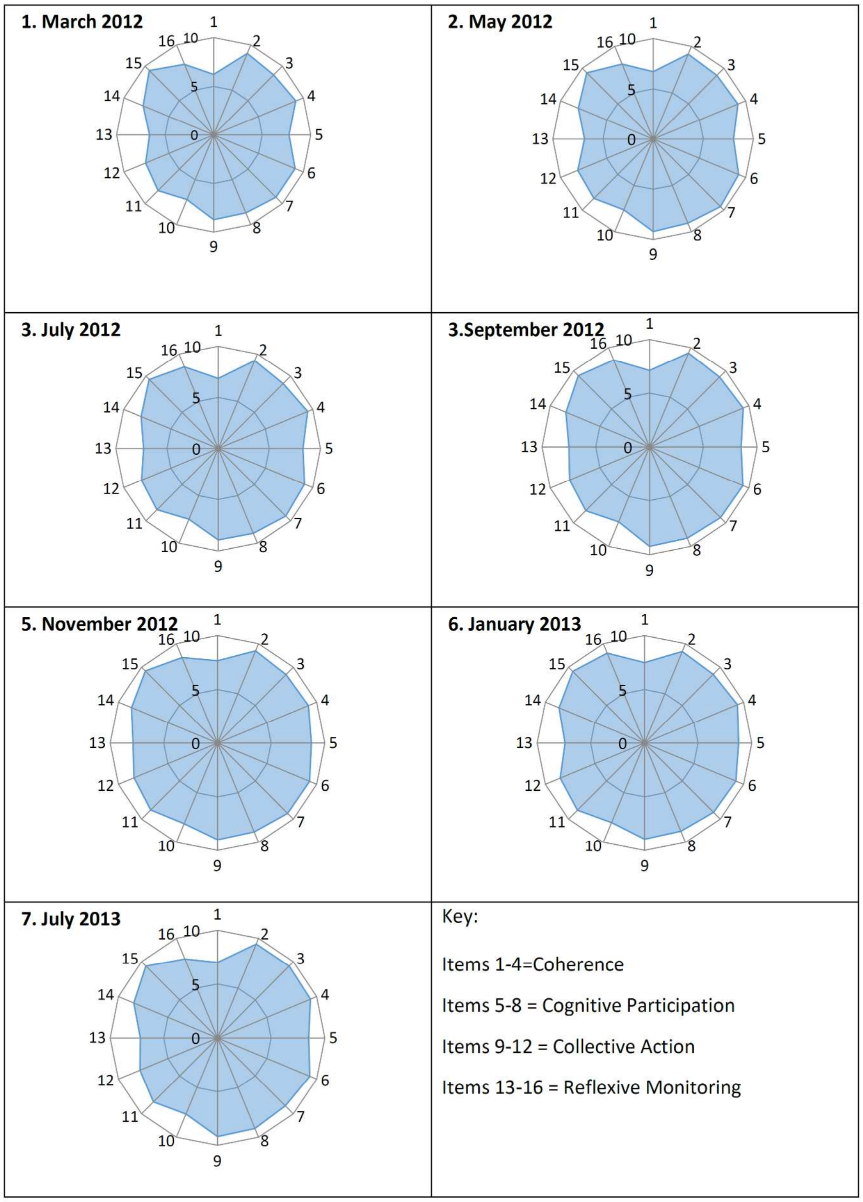

Figure 5: Radar plots showing the mean score for each sub-construct of NPT
Results from the NPT questionnaire taken throughout the course of the study.

239x332mm (300 x 300 DPI)

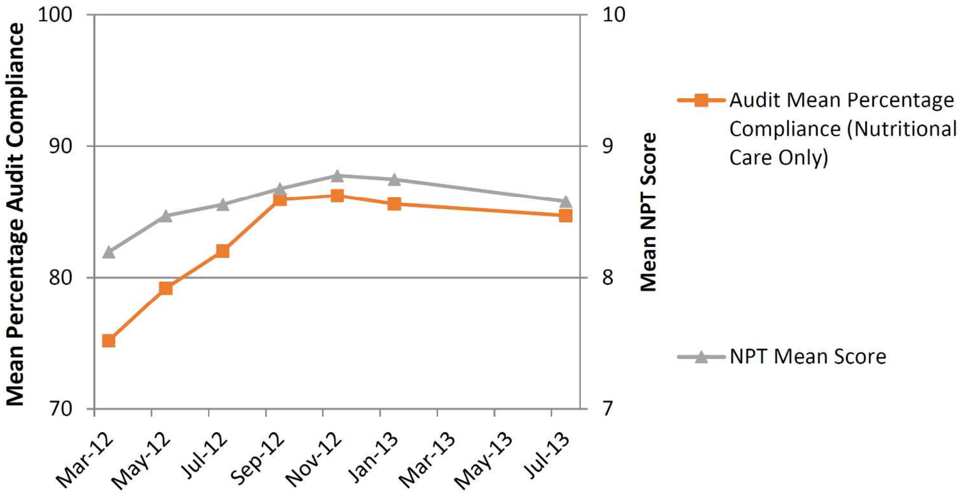

Figure 6: Relationship over time between mean NPT scores and percentage guideline compliance

224x117mm (300 x 300 DPI)

For peer review only

University Hospital Southampton NHS

Guidelines for the Nutritional Care of Infants in the Neonatal Unit

For peer review only

Version: 1.0

Issued: December 2011

Review date: December 2014

Author: Dr Alison Leaf

The procedural aspects of this guideline can be found in the document entitled:-

Guideline Proforma **- Guidelines for the Nutritional Care of Infants in the Neonatal Unit**

Disclaimer: It is your responsibility to check against StaffNet that this printout is the most recent issue of this document

For peer review only - http://bmjopen.bmj.com/site/about/guidelines.xhtml

Clinical Guidance

University Hospital Southampton NHS
NHS Foundation Trust

Contents

Paragraph		Page
	**Executive Summary**	3
1.	Introduction	4
2.	Definitions	5
3.	Roles and Responsibilities	6
4.	Related Trust Documents	7
5.	Guideline	
	**1. Aims and objectives; audit points**	8
	**2. Assessment and monitoring**	
	i. Initial assessment: growth / risk category / screen	9
	ii. On-going assessment: growth / biochemistry / nutrition screening / nutrition team	10
	**3. Nutrient requirements**	11
	**4. Standard nutritional support of preterm and sick infants**	12
	a. Overview by risk group	12
	b. Parenteral nutrition	13
	i. Indications for PN	13
	ii. Starting PN	13
	iii. Stock PN solutions	13
	iv. Pharmacy-manufactured (‘bespoke’) PN	13
	v. Reducing PN as feeds increase	14
	vi. Peripheral PN	14
	vii. Cautions	14
	c. Enteral nutrition	15
	i. Starting feeds	15
	ii. Choice of milk	15
	iii. Advancing feeds	15
	iv. Routine nutritional supplements	16
	v. Nutrition at discharge	16
	**5. Management of common gut and feeding problems**	17
	a. Feed intolerance / gastric residuals	17
	b. Abdominal distension	17
	c. Suspected Necrotising Enterocolitis (NEC)	17
	d. Suspected Gastro-oesophageal reflux (GOR)	17
	e. Suspected Food Protein Intolerance	17
	**6. Management of babies with surgical bowel problems**	18
	**7. Flow Charts**	20
	a. Starting and Increasing Feeds – High Risk Infants	20
	b. Starting and Increasing Feeds – Moderate Risk Infants	21
	c. Management of common feed related problems	22
	d. Choice of milk	23
	**8. Tables**	24
	a. Starting and increasing feeds	24
	b. Nutrient content of commonly used products	25
	**9. Supporting Information**	26
6.	References	32
**Appendices**		
Appendix 1	Nutrient Requirements of preterm infants	35
Appendix 2	Neonatal Nutrition Screening Tool	36

Disclaimer: It is your responsibility to check against StaffNet that this printout is the most recent issue of this document

For peer review only - http://bmjopen.bmj.com/site/about/guidelines.xhtml

University Hospital Southampton NHS
NHS Foundation Trust

Executive Summary

Good nutrition is important at all stages of life. Babies are born at a time of rapid growth and formation of body tissues and organs, yet immature metabolism means they are unable to cope with either excess or lack of nutrients. Detail in both the quantity and quality of nutrients is critically important.

There is good evidence that mother's breast milk confers many advantages to baby, mother and to the formation of the parental bond. As well as containing just the right nutrients for human development, breast milk contains many factors which promote immune function and enable healthy intestinal development. Breast milk and breast-feeding should be encouraged in almost all situations.

Preterm infants and those with congenital abnormalities or metabolic disorders may require nutrient supplements or special feeds, and may require a period of intravenous nutrition until the gut is able to support their needs.

Measuring growth and monitoring biochemical well-being is crucial to optimising nutrition in high risk individuals.

These guidelines aim to provide both practical and theoretical guidance for the optimal nutrition of sick and preterm infants in the NNU at Southampton.

For peer review only

Disclaimer: It is your responsibility to check against StaffNet that this printout is the most recent issue of this document

For peer review only - http://bmjopen.bmj.com/site/about/guidelines.xhtml

University Hospital Southampton NHS
NHS Foundation Trust

1. Introduction

- Good early growth is essential for long term health and well-being of all babies.
- Achieving recommended nutrient intake in very low birth-weight and sick infants is difficult particularly in the first weeks of life and development of a significant nutrient deficit is common. It is then very difficult to 'catch up'.
- Protein intake is particularly difficult to achieve.
- These guidelines aim to support decision-making such that nutrient delivery can be optimised. Close monitoring of intakes, biochemical status and growth is essential to monitor how well this is achieved.
- ***Every feed and every day is important – being aware of daily intake of key nutrients is the first step to improving growth and development***
- SENNAT (Southampton Electronic Neonatal Nutrition Assessment Tool) has been developed to help us all measure and monitor nutrient intakes and growth

These guidelines are based on recommendations of:

- Enteral nutrient supply for preterm infants: commentary from the European Society of Paediatric Gastroenterology, Journal of Pediatric Gastroenterology and Nutrition 2010[1]

- Nutrition of the Preterm Infant: Scientific basis and Practical Guidelines (second edition). Tsang RC, Uauy R, Koletzko B, Zlotkin S. Digital Educational Publishing 2005[2]

- Guidelines on Paediatric Parenteral Nutrition of the European Society of Paediatric Gastroenterology, Hepatology and Nutrition (ESPGHAN) and the European Society for Clinical Nutrition and Metabolism (ESPEN), Supported by the European Society of Paediatric Research (ESPR), Journal of Pediatric Gastroenterology and Nutrition 2005[3]

- Vermont Oxford Network 'Potentially Better Practices (PBPs) for Nutrition' as laid out in Pediatrics, 2003[4]

- Management and support of infant feeding in maternity facilities. Infant and young child feeding : model chapter for textbooks for medical students and allied health professionals., World Health Organisation 2009[5]

- Optimal feeding of low-birth-weight infants, World Health Organisation, 2006[6]

- UNICEF Baby Friendly Initiative, http://www.unicef.org.uk/babyfriendly

Disclaimer: It is your responsibility to check against StaffNet that this printout is the most recent issue of this document

For peer review only - http://bmjopen.bmj.com/site/about/guidelines.xhtml

2. Definitions

AREDF	Absent or Reversed End Diastolic Flow (in umbilical artery, seen on antenatal scans)
AXR	Abdominal X-Ray
BMF	Breast Milk Fortifier
CPAP	Continuous Positive Airways Pressure
D/C	Discharge
DBM	Donor Breast Milk
DH	Department of Health
ELBW	Extremely Low Birth Weight (birth weight <1000g)
FBC	Full Blood Count
g	grams
IU	International Units
IUGR	Intrauterine Growth Restriction
IV	Intravenous
kcal	kilocalories
kg	kilogram
LBW	Low Birth Weight (birth weight <2500g)
LFT	Liver Function Tests
MBM	Maternal Breast Milk
mg	milligram
ml	millilitre
mmol	millimole
NBM	Nil By Mouth
NEC	Necrotising Enterocolitis
NICU	Neonatal Intensive Care Unit
NNU	Neonatal Unit
PBP	Potentially Better Practice
PDA	Patent Ductus Arteriosus
PDF	Post Discharge Formula
PN	Parenteral Nutrition
RCT	Randomised Controlled Trial
SD	Standard Deviation
TAT	Trans-anastamotic Tube
TPN	Total Parenteral Nutrition
U&E	Urea and Electrolytes
VLBW	Very Low Birth Weight (birth weight <1500g)
VON	Vermont Oxford Network

For peer review only

For peer review only - http://bmjopen.bmj.com/site/about/guidelines.xhtml

3. Roles and Responsibilities

BREAST-FEEDING AND LACTATION SUPPORT

- All staff: awareness of Trust Policy and NNU Guidelines
- 'Breast-feeding babes' – Lead Sandy Jackson: expert guidance for mothers breast-feeding on the post-natal wards
- NNU lactation support team – Lead Jess Macfarlane: expert guidance for mothers breast-feeding and/or expressing milk in NNU

PARENTERAL NUTRITION

- All staff: awareness of need for PN in high risk infants
- Nursing staff: awareness of location of 'stock' PN in NNU and knowledge and skills for PN administration appropriate to nursing skill level
- Medical staff: awareness of PN supplies available and how to prescribe; awareness of potential complications of PN and how to avoid
- Pharmacists: Amanda Bevan and Zoe Lansdowne: expertise in detailed composition of PN solutions and provision of PN in different situations on NNU

ENTERAL NUTRITION

- All staff: support for mothers in choice of feeding
- All staff: awareness of choices for enteral nutrition: maternal breast milk / breast-feeding; donor breast milk / milk bank; standard infant formula; formulas for preterm infants; special formulas for infants with specific gut or feeding problems
- Neonatal Dietitian (Anita Emm): expert knowledge of composition of breast milk and alternatives and guidance on making appropriate choices
- Surgical team: expert knowledge on potential feeding challenges in infants with congenital or acquired abnormalities of the gut, particularly following surgery.

FEEDING DIFFICULTIES

- All staff: awareness of common feeding difficulties of preterm infants and those with neurological complications
- Speech and language therapist: expert knowledge of structure and function of upper gastro- intestinal tract and how to optimise feeding potential of vulnerable babies

GROWTH MONITORING

- All staff: Awareness of importance of making accurate and regular measurements and plotting them on appropriate charts to monitor growth
- Nursing staff: Weigh babies at intervals as indicated by clinical condition (ideally three times per week)
- Medical and Nursing staff: Measure head circumference and length at intervals as indicated by clinical conditions (ideally head circumference at least weekly and length at least fortnightly)
- Medical and Nursing staff: Plot growth measurements on appropriate chart weekly (provided competent to do so)

SPECIAL CASES

- Neonatal Nutrition Team: Will review high risk or complex patients on weekly nutrition ward round

Disclaimer: It is your responsibility to check against StaffNet that this printout is the most recent issue of this document

For peer review only - http://bmjopen.bmj.com/site/about/guidelines.xhtml

4. Related Trust Documents

Donor Breast Milk Guideline (to be found at: http://staffnet/TrustDocsMedia/DeptDivSpecific/DivC/WomenNewborn/NeonatalUnit/NeonatalGuidelines/DonerBreastMilkGuideline/DonorBreastMilkGuideline.doc)

Breastfeeding care pathway (on Neonatal Unit Guidelines on Unit Desktop PCs)

Vitamins and supplements guideline (on Neonatal Unit Guidelines on Unit Desktop PCs)

Parenteral Nutrition Guidebook, 4th edition (Hard copies in nurseries on Neonatal Unit)

Princess Anne Breastfeeding Policy (to be found at http://staffnet/TrustDocsMedia/DeptDivSpecific/DivC/WomenNewborn/Obstetrics/ObstetricClinicalGuidelines/BreastfeedingTermInfantsGuideline/BreastfeedingTermInfantsGuideline.doc)

Neonatal Unit Breastfeeding and Formula Feeding Guideline (currently being written)

Neonatal Surgical Clinical Aids (to be found at: http://staffnet/Departments/DivisionC/Womenandnewborn/Neonatalservices/Neonatalsurgery/Neonatalsurgeryclinicalaids/Neonatalsurgeryclinicalaids.aspx)

Central Venous Access Guideline (currently being written)

Naso/Orogastric Tubes in Neonates - the safe placement of: Guidelines (to be found at:http://staffnet/TrustDocsMedia/DeptDivSpecific/DivC/WomenNewborn/NeonatalUnit/NeonatalGuidelines/NasoOrogastricTubesinNeonates-thesafeplace/NasoOrogastricTubesinNeonates-thesafeplacementofGuidelines.DOC)

Disclaimer: It is your responsibility to check against StaffNet that this printout is the most recent issue of this document

For peer review only - http://bmjopen.bmj.com/site/about/guidelines.xhtml

University Hospital Southampton NHS
NHS Foundation Trust

5. Guideline Information

1. AIMS AND OBJECTIVES

- To optimise use of breast milk and breast-feeding
- To achieve recommended nutrient intakes
- To achieve postnatal growth and body composition approximating fetal growth.
- To reduce the risk of nutritional deficiency states such as late anaemia of prematurity or metabolic bone disease.
- To reduce the risk of feeding related morbidities such as NEC or cholestasis
- To optimise long term neurodevelopmental outcome.

KEY PRINCIPLES

- All babies should be measured and have nutritional risk assessment on admission, and weekly during their stay
- Nutrition support should be started early: PN for high risk; enteral feeds for lower risk
- Mother's breast milk is the feed of first choice
- Feed tolerance should be assessed regularly and managed according to algorithms
- Protein intake should be documented and optimised in preterm infants
- High risk babies should be seen each week by the Nutrition Team
- Nutrition and feeding should be discussed in Discharge Planning and documented in the notes

AUDIT POINTS

- Use of Nutrition Screening Tool, on all NNU admissions (100%)
- Use of growth charts on all NNU admissions (100%)
  - Weight and Head Circumference plot weekly; length plot 2-weekly
- Lactation advice and support by 6 hours for all mothers of VLBW infants
  - 100% - unless mother too ill
- Breastfeeding rates at discharge
- Protein and energy intakes as recommended by Tsang 2005
- Use of nutritional supplements according to Guidelines
- Documentation of Nutrition Plan at discharge (100%)

Disclaimer: It is your responsibility to check against StaffNet that this printout is the most recent issue of this document

For peer review only - http://bmjopen.bmj.com/site/about/guidelines.xhtml

2. ASSESSMENT AND MONITORING

(i) INITIAL ASSESSMENT

a. <u>Growth Measurement</u>

All infants should have weight, length and head circumference measured and plotted on the appropriate growth chart at admission. This information, together with other risk factors detailed below, will identify the degree of 'nutritional risk' – ie risk of becoming malnourished or developing nutrition and feeding related problems. Infants with multiple risk factors should be classified according to their highest individual risk factor. This will guide nutritional care and allow subsequent progress to be monitored.

b. <u>Risk assessment</u> – identify level of risk for nutrition and / or feeding-related problems

High risk

- Preterm <28 weeks
- ELBW < 1000g
- Severe IUGR (weight < 2nd centile with AREDF) <35 weeks
- Infant establishing feeds after episode of NEC or GI perforation
- Infants with severe congenital GI malformation: e.g. gastroschisis
- Severe Perinatal hypoxia / ischaemia

Moderate risk

- Preterm 28-31^{+6} weeks, otherwise well
- VLBW 1000 – 1500g
- Moderate IUGR (weight < 9th centile with AREDF) <35 weeks
- Baby on inotropes
- Baby on indomethacin/ibuprofen (NB avoid concomitant treatment with steroids)
- Baby >1500g with illness or congenital anomaly which may compromise feeding
- Symptomatic polycythaemia, with PCV ≥ 70%

Low risk

- Preterm 32-36^{+6} weeks, otherwise well
- AREDF / IUGR ≥35 weeks
- Term Infants >37 weeks

Disclaimer: It is your responsibility to check against StaffNet that this printout is the most recent issue of this document

For peer review only - http://bmjopen.bmj.com/site/about/guidelines.xhtml

University Hospital Southampton NHS
NHS Foundation Trust

(ii) ON-GOING ASSESSMENT AND MONITORING

a. GROWTH

i. Weight should be measured at least twice a week, and plotted on CLOSE MONITORING WHO growth chart weekly. More frequent weights required for some babies should be plotted on a daily weight chart

ii. Head circumference should be measured and plotted weekly

iii. Length should be measured and plotted within the first week, and every 2 weeks thereafter.

iv. If a baby is too sick to be weighed and measured so cannot be plotted, mark the bottom of the growth chart at date with a triangle (△) at the day's date.

v. Targets for weight – changes in weight in the early days of life usually reflect fluid balance: aim for weight loss of no more than 10% from birth weight. Once baby is stable and growing, aim for gain of 15-20 grams/kg/day

vi. Head circumference and length: normally expect increase of 0.75 cm/week

b. BIOCHEMISTRY

i. **First week of PN**:
- Full TPN Profile daily (FULL IP MG on eQuest, this includes U&E's, Calcium, magnesium phosphate and LFTs)
- FBC twice weekly

ii. **Second and subsequent week of PN**:
- Full TPN Profile and FBC twice weekly if stable (daily if still unstable)

iii. **Triglycerides** should be measured weekly (ideally Mondays)when on IV lipid

iv. **If on PN for longer than 1 month, then Trace elements (Zn, Cu, Se, Mn – use special blood bottle in Dr's Office) and Vitamins (A, D and E) should be measured monthly. Consider measuring Iron status and clotting**

v. **When on enteral feeds:**
- Infants in the High and Medium risk categories need weekly FBC, U&Es, LFTs and Bone profiles once they are off PN and fully enterally fed. This can be extended to once fortnightly when babies are moved into Special Care.

c. SCREENING

i. A Neonatal Nutrition Screening form should be completed on admission and on Sunday/Monday when the baby has been weighed and measured each week on all babies to identify those requiring nutrition team review

d. NUTRITION TEAM REVIEW

i. Nutrition ward rounds take place on Tuesday mornings from 0900-1100. Nutrition team will see all 'high-risk' babies, and any others identified by nutritional screening on Sunday/Mondays.

Disclaimer: It is your responsibility to check against StaffNet that this printout is the most recent issue of this document

For peer review only - http://bmjopen.bmj.com/site/about/guidelines.xhtml

3. NUTRIENT REQUIREMENTS

Nutrient requirement for Term and Preterm infants in the first weeks of life are summarised below. The figures shown below are based on the parenteral requirements for the first week, and the enteral requirements for the subsequent weeks (for a full list of parenteral and enteral requirements see Appendix 1).

Term infants – based on intake in 150 ml/kg breast milk; preterm infants based on recommendations in Tsang 2005 unless otherwise stated.

There are no specific guidelines for those babies born over 1.5kg and under term weight (2.5 kg) but it can be anticipated that their nutritional needs will be between those of preterm infants and term infants. Nutritional support should therefore aim to deliver nutrient intakes in this area.

It should be noted that these are just recommendations, and some infants may require more of certain nutrients such as Sodium and Potassium as dictated by the results of blood tests.

**Nutrient Unit/kg/day**	**Term infant**	**Preterm VLBW 1000-1500g 1st week (parenteral)**	**Preterm VLBW 1000-1500g After 1st week (enteral)**	**Preterm ELBW < 1000g 1st week (parenteral)**	**Preterm ELBW < 1000g After 1st week (enteral)**
Energy (kcal)	100	60-70	110-130	75-85	130-150
Protein (g)	1.5-2.1	3.5	3.4-4.2	3.5	3.8-4.4
Nitrogen (g)	0.24-0.34	0.56	0.54-0.61	0.56	0.61-0.70
Sodium (mmol)	1.4	2.0-5.0	3.0-7.0	2.0-5.0	3.0-7.0
Potassium (mmol)	2.0	0-2.0	2.0-3.0	0-2.0	2.0-3.0
Calcium (mmol)	1.25	1.5	2.5-5.5	1.5	2.5-5.5
Phosphate (mmol)	1.3	1.5-1.9	1.9-4.5	1.5-1.9	1.9-4.5
Vitamin D IU*	340	40-160	800-1000	40-160	800-1000
Vitamin A IU**	1150	700-1500	700-1500	700-1500	700-1500
Iron (umol)	17.9	0	35.8-71.6	0	35.8-71.6

*Vitamin D = dose quoted is total daily dose; ESPGHAN 2010 recommendation for enteral dose for preterm infants; term infants DH Dietary Reference Values 1991 (340 IU = 8.5 mcg Vit D)

**Vitamin A = dose quoted is total daily dose; term infants DH Dietary Reference Values 1991 (1150 IU = 350 mcg of Vitamin A retinol equivalent)

Disclaimer: It is your responsibility to check against StaffNet that this printout is the most recent issue of this document

For peer review only - http://bmjopen.bmj.com/site/about/guidelines.xhtml

4. STANDARD NUTRITION SUPPORT –

(a) OVERVIEW - GETTING STARTED - EARLY TPN AND TROPHIC MILK FEEDS

HIGH RISK / MEDIUM RISK (see flow charts for high [A] and medium risk preterm infants [B])

- Aim to introduce milk feeds gradually while maintaining calorie and nutrient intake with PN
- Before starting or increasing milk ensure baby is clinically stable and abdomen soft
- Ensure mother has lactation support to start expressing (see breastfeeding care pathway)

High risk preterm (<28 weeks; <1000g; severe IUGR/AREDFV <35 weeks)

Day 1	Start Stock Preterm PN at 60-90 ml/kg/day via UVC or long line, as soon as possible unless baby very unstable. Give fresh colostrum as mouth care or as trophic feeds
Day 2-3	Start trophic feeds: MBM 1 ml/kg 2-4 hourly (if no MBM can use DBM- see choice of milk chart);
Day 3-7	Change to Stock Preterm + Sodium PN when 6% weight loss from birthweight [7], additional sodium required, or by day 5, whichever soonest Increase milk by 10-20 ml/kg/day as tolerated (see table); Aim to decrease PN flow rates with feeds only once baby on total fluids of 180ml/kg/day

Moderate risk preterm (28-31^{+6} weeks; 1000g <1500g; mod IUGR/AREDFV < 35 weeks)

Day 1	Start Stock preterm PN at 60-90 ml/kg/day via UVC or long line as soon as possible; if no central access consider peripheral PN
Day 1-2	Start colostrum/milk 1 ml/kg 2 hourly ('see choice of milk' chart)
Day 3-7	Change to Stock Preterm + Sodium PN when 6% weight loss, or by day 5, whichever is sooner. Aim to decrease PN flow rates with feeds only once baby on total fluids of 180ml/kg/day Increase milk by 20-30 ml/kg/day according to clinical condition and tolerance;

High / moderate risk term or near-term infants

All high/moderate risk babies should have a plan for nutrition support on admission and periods greater than 48 hours without protein and micronutrients should be unusual

Low risk

Day 1	Commence milk feeds 30-60 ml/kg/day, supplemented by IV fluids if necessary
Day 2-7	Increase milk feeds by 30 ml/kg/day as tolerated

NOTES

- If severely unwell or acidotic, PN may need to be delayed (though contains acetate)
- Babies with HIE undergoing therapeutic hypothermia, may tolerate trophic milk feeds
- For babies with surgical problems , see 'surgical guidelines' – section 6

Disclaimer: It is your responsibility to check against StaffNet that this printout is the most recent issue of this document

For peer review only - http://bmjopen.bmj.com/site/about/guidelines.xhtml

University Hospital Southampton NHS
NHS Foundation Trust

4. (b) PARENTERAL NUTRITION

i) Indications for PN

- High or Moderate risk infants as described above
- Infants who are NBM and unlikely to achieve adequate milk intake in the next 5 days
- Infants who are not tolerating feeds such that they cannot take full feed volumes for 5 consecutive days

ii) Starting PN

- In high and moderate risk infants PN should be started as soon as possible as delay can result in significant and cumulative nutrient deficits.
- Birth weight ≤1500g – start as soon as possible after birth
  - Ideally within 6 hours
- Birth weight >1500g – if enteral feeding contra-indicated, start PN by
  - 48 hours in 1500-2500g
  - 72 hours in 2500-3500g if NBM
- Central line insertion (UVC or peripherally inserted central venous line) should be a priority for high and moderate risk infants
- If feeds are stopped on high or medium risk infant for any reason, re-stat PN

iii) Stock PN

- Infants should be started on Stock PN in the first instance as detailed below:
  - Preterm PN – For preterm infants (<37/40 gestation) where additional sodium is not indicated (ie until 6% weight loss, or day 5 of life)
  - Preterm + Sodium PN- For preterm infants (<37/40 gestation) requiring maintenance sodium. **This should be the PN of choice for the majority of preterm infants after the first few days following birth**, as it contains more protein.
  - Term PN – for Term infants (≥37 weeks gestation) at any point after birth.
- Stock PN comprises an aqueous solution (glucose, amino acids, electrolytes and trace elements) and a lipid solution (**which contains both fat- and water-soluble vitamins**). For adequate nutrition it is **important that the lipid is always given with the aqueous solution** at all times (except when well advanced on enteral feeds - see below).

iv) Pharmacy made ('bespoke') PN

- Neither PN alone nor unfortified full breast milk feeds fully meet the nutritional needs of preterm infants, so the period when a preterm infant transitions from PN to milk feeds is when they are at highest risk of poor nutrient intakes.
- Stock PN is designed to give the maximum possible nutrition at 130ml/kg/day. **Therefore, pharmacy can make bespoke PN, which provides more nutrition in a smaller volume, should be used whenever a preterm infant is receiving less than 130ml/kg/day of Stock PN**. This will occur whenever a preterm infant is increasing on enteral feeds, is fluid restricted, or receiving other infusions
- Bespoke PN may also be appropriate where infants have electrolyte requirements than cannot be met with Stock PN

Disclaimer: It is your responsibility to check against StaffNet that this printout is the most recent issue of this document

For peer review only - http://bmjopen.bmj.com/site/about/guidelines.xhtml

v) Reducing PN as enteral feeds increase

- **Only once the infant is receiving 180ml/kg/day total fluids should the PN solution be decreased as enteral feeds increase** (unless there is a clinical decision to restrict fluids).
- Once the infant is on 90ml/kg/day enteral feeds, the rate of lipid infusion should be halved, and then stopped when the infant reaches 135ml/kg/day enteral feeds (beware with pharmacy made TPN as this reduction in lipid may have already been done as part of the prescription). Any shortfall in total fluid volume due to the reduction in lipid should be made up by increasing the aqueous PN solution, to allow maximum protein to be delivered to the infant (though do not go above the maximum prescribed rate). This is important when infants are on Stock PN, but for those on bespoke PN, the reduction in lipid may have already been done/accounted for by the pharmacists when the PN was prescribed so may not be necessary (check with the pharmacists first). **Remember that once the lipid is stopped, vitamin intake will be inadequate until Abidec is started.**

vi) Peripheral PN

- PN should ideally be given via a central line. However, there are occasions in high nutritional risk infants with difficult access where the benefits of giving PN peripherally may outweigh the risks. Such decisions should be made by the Consultant responsible for the patient.

vii) Cautions on PN

SEPSIS - may affect lipid metabolism; measure triglycerides and if >2.8mmol/L consider reducing or stopping IV lipid for 12-24 hours in severely septicaemic baby (remember to restart/increase lipid when sepsis has resolved)

THROMBOCYTOPENIA – high concentration of polyunsaturated fats may impair platelet adhesion: reduce lipid to 1-2 g/kg/day if platelets <50.

CHOLESTATIC JAUNDICE – total and prolonged PN increases the risk, so try to give some enteral feed if at all possible; other risk factors include IUGR, sepsis and short bowel syndrome. Lipid solutions containing fish oil (eg SMOF) can reduce or reverse cholestasis, and should be considered in high risk babies if on PN for 4 weeks or more. Alternate day lipid may also be indicated in this situation, or if altered liver function - discuss with the pharmacists.

Disclaimer: It is your responsibility to check against StaffNet that this printout is the most recent issue of this document

For peer review only - http://bmjopen.bmj.com/site/about/guidelines.xhtml

University Hospital Southampton NHS
NHS Foundation Trust

4 (c) ENTERAL NUTRITION

i. **Starting feeds** – see section 4(a) for guidance. Before starting feeds ensure baby is clinically stable and abdomen soft. In high-risk infants trophic feeding should be started within the first 72 hours if at all possible to minimise intestinal mucosal atrophy, and continued until ready to progress.

ii. **Choice of milk** – Mother's breast milk is almost always the feed of first choice, unless contraindicated by maternal illness or drugs. If no maternal milk available pasteurised donor breast may be used for high risk babies (parental consent required) in accordance with the DBM guideline. Preterm formula (LBW/Aptamil Preterm) is indicated for infants with gestation <34 weeks, or birth weight <1800 grams; Post discharge formula (Nutriprem 2) is indicated for preterm infants either as sole diet or in addition to breast-feeding from around 36 weeks (or at discharge) up to 6 months corrected. (see Flow Chart D)

iii. **Advancing feeds** – see section 4 (a) for guidance on volumes

- Before starting or increasing milk ensure baby is clinically stable and abdomen soft. Small gastric residuals can be tolerated if baby well. Passage of meconium and then changing stools is an important indication of gut motility. Glycerine suppositories may be useful if no stool passed for 48 hours.
- Feeds can be increased by 10-20ml/kg/day in high-risk, 20-30ml/kg/day in moderate risk and 30 ml/kg/day in low risk babies
- Test for residuals 4-6 hourly
- If baby vomits, or has residuals >25% of the previous 4 hours total feed volume and persisting or increasing examine and assess baby and refer to flow chart C

iv. **Nutritional supplements**

- BREAST MILK FORTIFIER (BMF, see high risk and moderate risk flow charts A and B) - 'multi-component' fortifier provides additional calories (carbohydrate), protein (cows' milk based), minerals and vitamins in a powder which is added to mother's breast milk. It should be more or less routine for babies with birth weight <1500g to receive fortifier once they have tolerated 150 mls/kg/day of MBM for 24 hours, unless significant gut or renal compromise. Blood Urea and albumin levels provide useful markers of protein status. In general, give ½ strength for 24-48 hours and then increase to full strength (2.2g sachet to 50 mls MBM), though it may be preferable to increase the fortifier by ¼s in high risk infants. For some extremely high risk infants it may be prudent to start fortifier when on 120-135 mls/kg/day of MBM and increase strength more gradually as PN is gradually reduced, in order to ensure the baby will be able to achieve enteral nutrient targets before stopping PN.

Disclaimer: It is your responsibility to check against StaffNet that this printout is the most recent issue of this document

For peer review only - http://bmjopen.bmj.com/site/about/guidelines.xhtml

- Vitamins and Iron – breast milk provides insufficient vitamins (particularly vitamin A and D) for preterm infants, and virtually no iron. Abidec (multivitamins) and Sytron (iron) should be started according to NNU guideline
- Electrolytes and minerals
  - Small doses should be given as boluses, as scheduled on drug chart
  - Sodium : aim to maintain serum sodium 135-145 mmol/L
    If on > 4 mmol/kg/day, add to daily feeds in milk kitchen; if < 4 mmol/kg/day, give as divided bolus drugs (ideally as a four times daily regimen)
  - Phosphate: content of BM is low. Aim to maintain serum inorganic phosphate levels greater than 1.8mmo/L. Usually given as Potassium Acid Phosphate 0.5-2mmol/kg/day. If required as outpatient, may be preferable to use BMF

v. Nutrition at discharge

It is important to start discharge-planning well in advance. Breast-feeding at discharge is the preferred goal for all infants. However for preterm infants nutritional supplementation will be required. For those not being breast fed advice has to be given on choice of formula, so for all infants a pre-discharge nutrition assessment should be made and plan documented.

MUM PLANNING TO BREAST FEED

- Ensure lactation support is on-going re feeding technique
- Discuss with Out-reach sister re support at home
- **All preterm infants (<35 weeks) should have Abidec (1 ml) and Sytron (1 ml) daily**
- Assess growth
  - If growth has been good and weight, length and HC are no more than 0.67 SD (ie one centile line) below birth levels, then assess weight gain after 48 hours. If satisfactory can go home breast-feeding
  - If baby has had significant post-natal growth restriction and is >1.33 SD below birth (2 centile lines), discuss with Nutrition team / Dietician and consider discharge on BMF, with Outreach Support
  - For those with modest growth restriction, i.e. between one and two centile line drop, review overall pattern of growth and consider requesting nutrition review and Outreach support.

MUM PLANNING TO FORMULA FEED

- Babies <34 weeks gestation, with birthweight <2kg can be considered for discharge on Post-Discharge Formula (PDF) – 'Nutriprem 2'. This should be continued until 3 to 6 months corrected age.
- ELBW and VLBW babies who have been on LBW formula should be changed to PDF at approximately 36 weeks corrected age, or when beginning to take most feeds by bottle. For those who have had severe extra-uterine growth restriction, continuing with LBW formula to 40 weeks corrected age may be appropriate.
- Babies discharged on PDF should have Abidec 0.6 ml, but not Sytron.
- If changing to term formula, prescribe Abidec 1 ml (continue until at least one year post term) and Sytron1ml (continue until 6 month post term)

SOLIDS **–** can be introduced at 5-8 months REAL AGE (ie not corrected for prematurity)

Disclaimer: It is your responsibility to check against StaffNet that this printout is the most recent issue of this document

For peer review only - http://bmjopen.bmj.com/site/about/guidelines.xhtml

5. **MANAGEMENT OF COMMON GUT AND FEEDING PROBLEMS** – see flow chart C

a. **Gastric aspirates / residuals** – preterm infants have immature gut motility, and aspirates/residuals and small vomits are not uncommon. Dark green bile stained aspirates, particularly in association with abdominal distension and / or tenderness are a cause for concern. However small milky / yellow aspirates up to 2-3 mls are frequently normal. They can be replaced, and feeds continued.

b. **Abdominal distension** – this is another common feature in preterm infants, due to poor gut motility. It tends to be more common in babies on nasal CPAP, with high volumes of air flowing into the upper airway and oesophagus. Tenderness, or systemic symptoms and signs such as apnoea, tachycardia or temperature instability should raise concern. If baby is otherwise well, a small glycerine suppository may help to stimulate peristalsis, and enable feeds to be continued.

c. **Suspected NEC** – classical features are blood and mucous in stools, bile stained aspirates and abdominal tenderness. Systemic signs such as tachycardia and hypotension occur in severe NEC. X-ray might show intramural gas (‘pneumatosis coli’), dilated loops of bowel, free air, or a ‘gas-less’ bowel. In suspected NEC feeds should be stopped, and urgent attention paid to supporting ventilation, circulation and fluid balance.

d. **Suspected GOR** – mild milk reflux is common in newborn babies, including those born preterm and is usually self-limiting. It is rarely the cause of significant cardio-respiratory disturbance. However, apnoea and bradycardia are common in preterm babies and may occur in association with feeds. Try to avoid using gaviscon in babies who are having fortified MBM as the milk becomes excessively thick.

e. **Suspected Food Protein Intolerance** – food protein (e.g. cow’s milk protein) intolerance can occur in young infants either breast fed or formula fed. Symptoms may include severe regurgitation, vomiting, constipation, peri-anal rash, blood in stools and iron deficiency anaemia. Non-intestinal features may include skin rash – atopic eczema, and colic. If this is thought to be the cause of symptoms, it is recommended that cow’s milk protein be excluded from diet. If breast feeding, mother should exclude both cows’ milk and egg products from her diet for two weeks, while continuing to breast feed. Formula fed infants should be tried on amino acid formula. If improvement is seen, a staged reintroduction should be carried out. If no improvement is seen on definite exclusion diet, food protein intolerance is unlikely. If exclusion diet is difficult to maintain, a trial of amino-acid formula may be breast fed infants. See review by Vandenplas et al.[8]

For peer review only - http://bmjopen.bmj.com/site/about/guidelines.xhtml

6. MANAGEMENT OF BABIES WITH SURGICAL BOWEL CONDITIONS WHICH MAY COMPROMISE NUTRITION

Information has been extracted from the NEONATAL SURGERY CLINICAL AIDS on SUHTranet:

(http://staffnet/Departments/DivisionC/Womenandnewborn/Neonatalservices/Neonatalsurgery/Neonatalsurgeryclinicalaids/Anorectalmalformations.aspx)

This website should be checked to ensure that the most up to date version of the guidance is used.

GASTROSCHISIS

All babies with gastroschisis will require TPN.

For those treated with a Medicina Silo insertion at the cot-side a percutaneous long line should be sited on the Neonatal Unit but line insertion should ideally be delayed until after gut manipulation has ceased, i.e. once the silo has been removed and the defect closed, to reduce the chance of line colonisation. The median time to closure is 4 days. If it is felt that TPN should be commenced before this time then this can be given via peripheral cannula. In babies in whom it is thought there may be a delay in defect closure it may be better to proceed with line insertion prior to closure. As some gastroschisis babies may go on to have intestinal failure and require long term central venous access, central lines should only be inserted by staff with considerable experience of line insertion so as to avoid loss of suitable veins.

If the baby is taken to theatre for primary closure or surgical silo creation a percutaneous long line can be inserted in theatre at the time if someone with the appropriate expertise is available.

Duration of TPN may vary from 10 days to 6 weeks with a mean of 3 weeks. In rare cases gut function may be impaired for many months.

DUODENAL ATRESIA

A trans-anastamotic tube (TAT) can be placed during surgery, which allows feeding into the jejunum. A naso/orogastric tube will also be required for gastric decompression. Usually a 6Fr enteral feeding tube is placed nasojejunally and an 8Fr nasogastric tube placed down the other nostril. In preterm babies this may produce problems due to obstruction to both nostrils. In this situation it may be better to pass an orogastric 8Fr tube and leave one nostril patent.

Poor duodenal contractility may delay normal oral feeding for as long as 3 weeks. This may be overcome by transanastamotic feeding although there is evidence that this may delay eventual oral feeding. It is NOT usually necessary to place a long line or commence TPN because of the use of TAT feeding. Duration of admission is about 7 - 10 days but may be longer if motility is very delayed.

EXOMPHALOS

Nutritional support: Most babies who have undergone primary closure will tolerate enteral feeding soon and not need TPN. Most babies with a silo will require a long line and TPN

For peer review only - http://bmjopen.bmj.com/site/about/guidelines.xhtml

MECONIUM ILEUS

Feeding may start when gut recovery from surgery allows. Usually start on MBM or standard formula feed grading up slowly. Feed may need to change to hydrolysed formula if weight gain inadequate on breast milk or standard formula. Occasionally TPN is needed.

80-90% of babies with MI are deficient in pancreatic enzymes, and supplementation with 'Creon®' may be required. Further details are provided in Surgical Clinical Aids and treatment will usually be guided by advice from the CF team

OESOPHAGEAL ATRESIA and TRACHEO-OESOPHAGEAL FISTULA

A trans-anastomotic tube (TAT) nasogastric tube will be placed at time of surgery and feeding usually commences via the TAT at 48hrs post-op. If the TAT falls out do not re-pass as this may perforate the anastomosis. Consult the surgical team immediately.

Oral feeding normally starts between 3 and 5 days post-op at the discretion of the surgical team.

Gastro-oesophageal reflux prophylaxis: some surgeons use ranitidine post-op for 3 - 6 months. Others do not.

Disclaimer: It is your responsibility to check against StaffNet that this printout is the most recent issue of this document

For peer review only - http://bmjopen.bmj.com/site/about/guidelines.xhtml

7. FLOW CHARTS

a. Starting and Increasing Feeds- HIGH RISK INFANTS

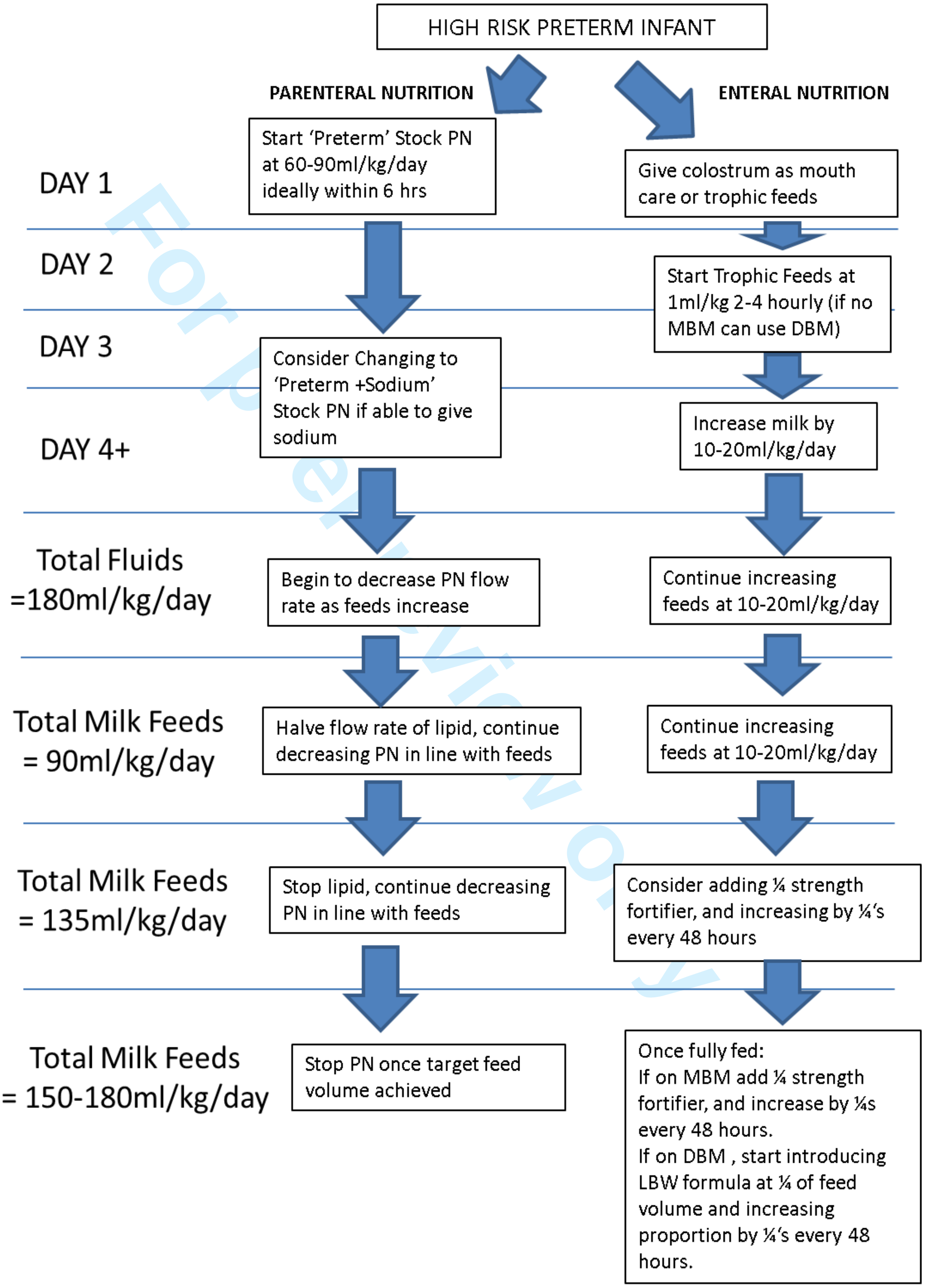

Disclaimer: It is your responsibility to check against StaffNet that this printout is the most recent issue of this document

For peer review only - http://bmjopen.bmj.com/site/about/guidelines.xhtml

Clinical Guidance

University Hospital Southampton NHS
NHS Foundation Trust

b. **Starting and Increasing Feeds- MODERATE RISK INFANTS**

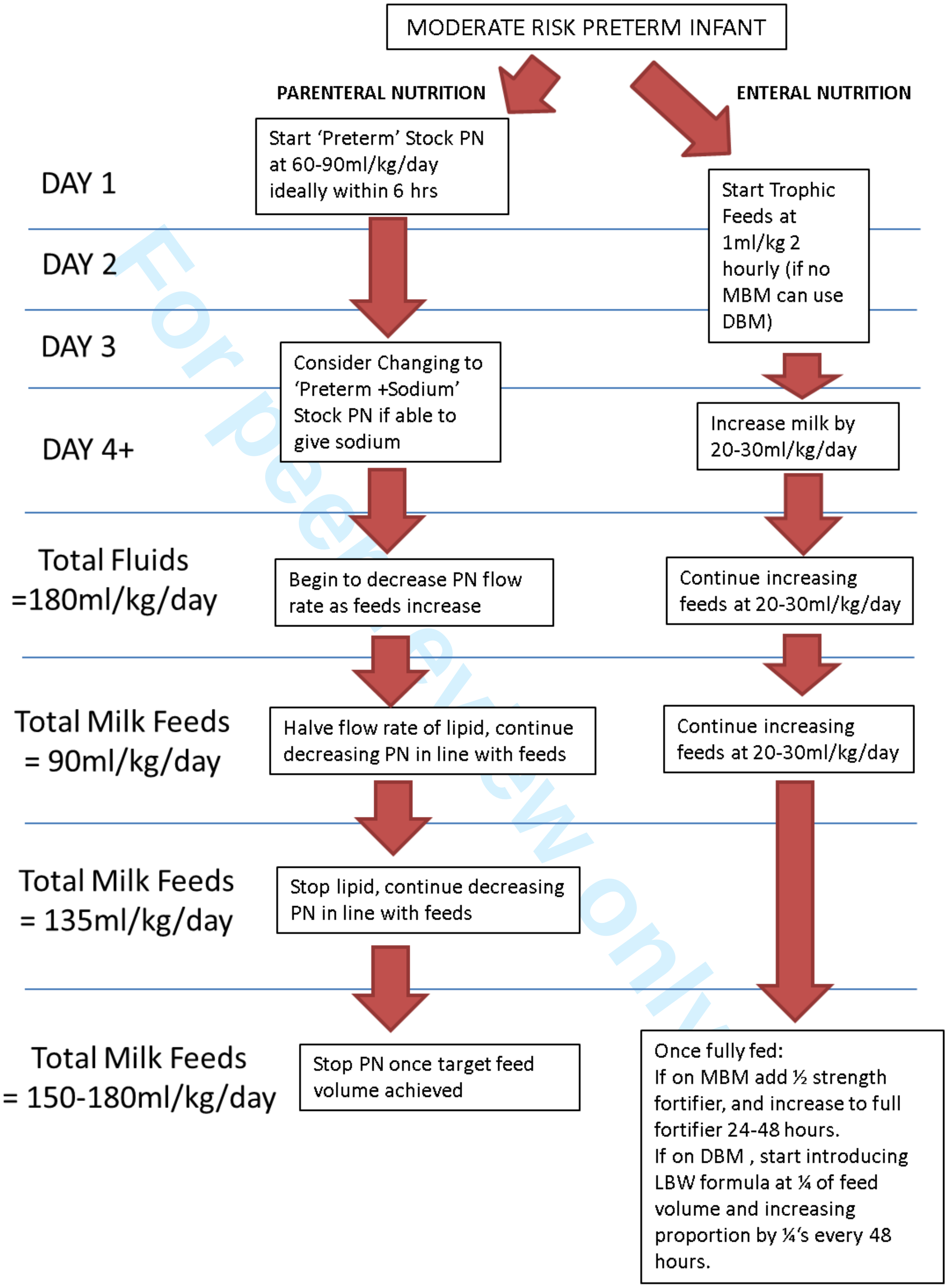

Disclaimer: It is your responsibility to check against StaffNet that this printout is the most recent issue of this document

For peer review only - http://bmjopen.bmj.com/site/about/guidelines.xhtml

University Hospital Southampton NHS
NHS Foundation Trust

b. Management of common feed-related problems

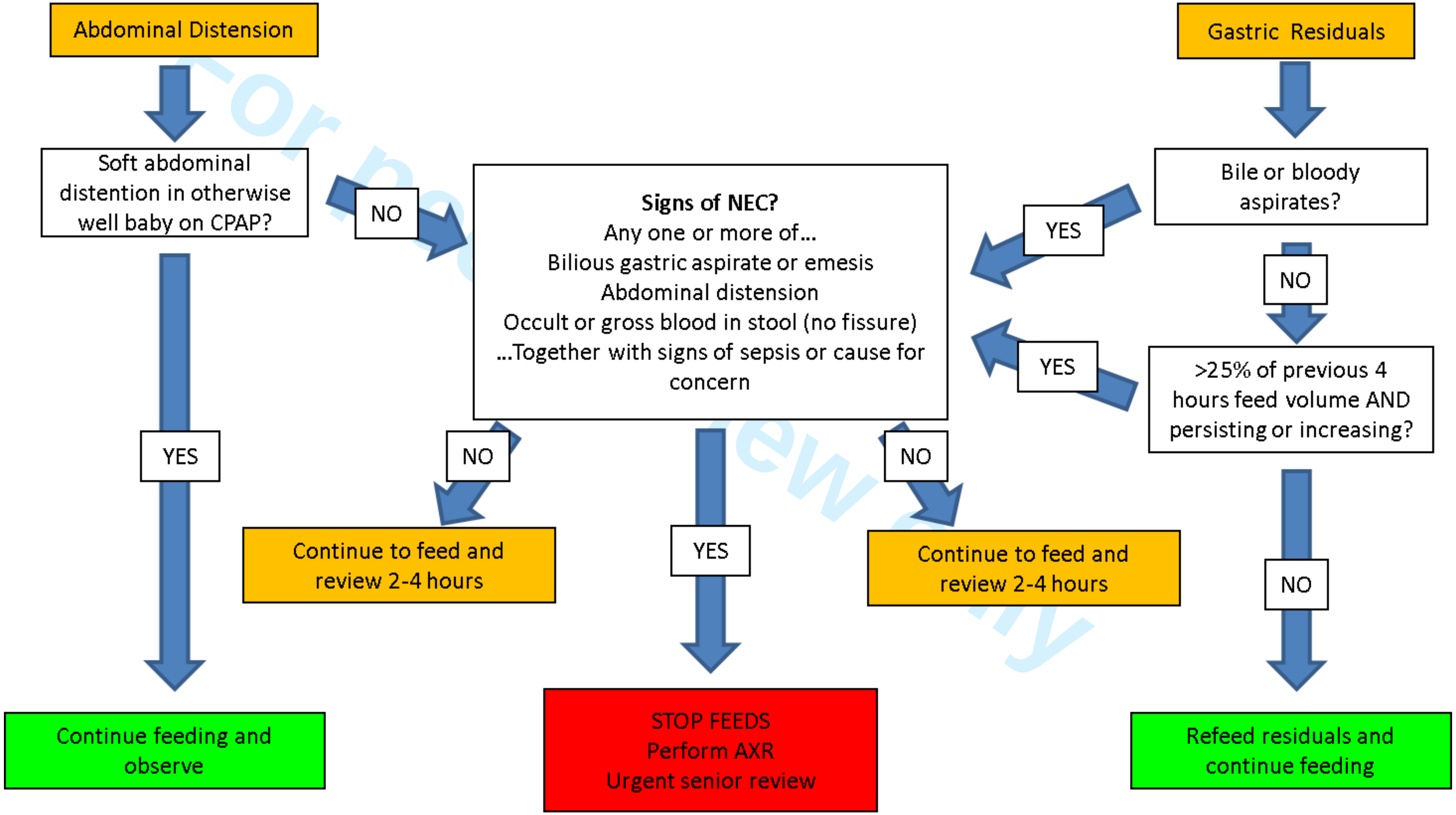

Disclaimer: It is your responsibility to check against StaffNet that this printout is the most recent issue of this document

For peer review only - http://bmjopen.bmj.com/site/about/guidelines.xhtml

University Hospital Southampton NHS
NHS Foundation Trust

c. Choice of Milk

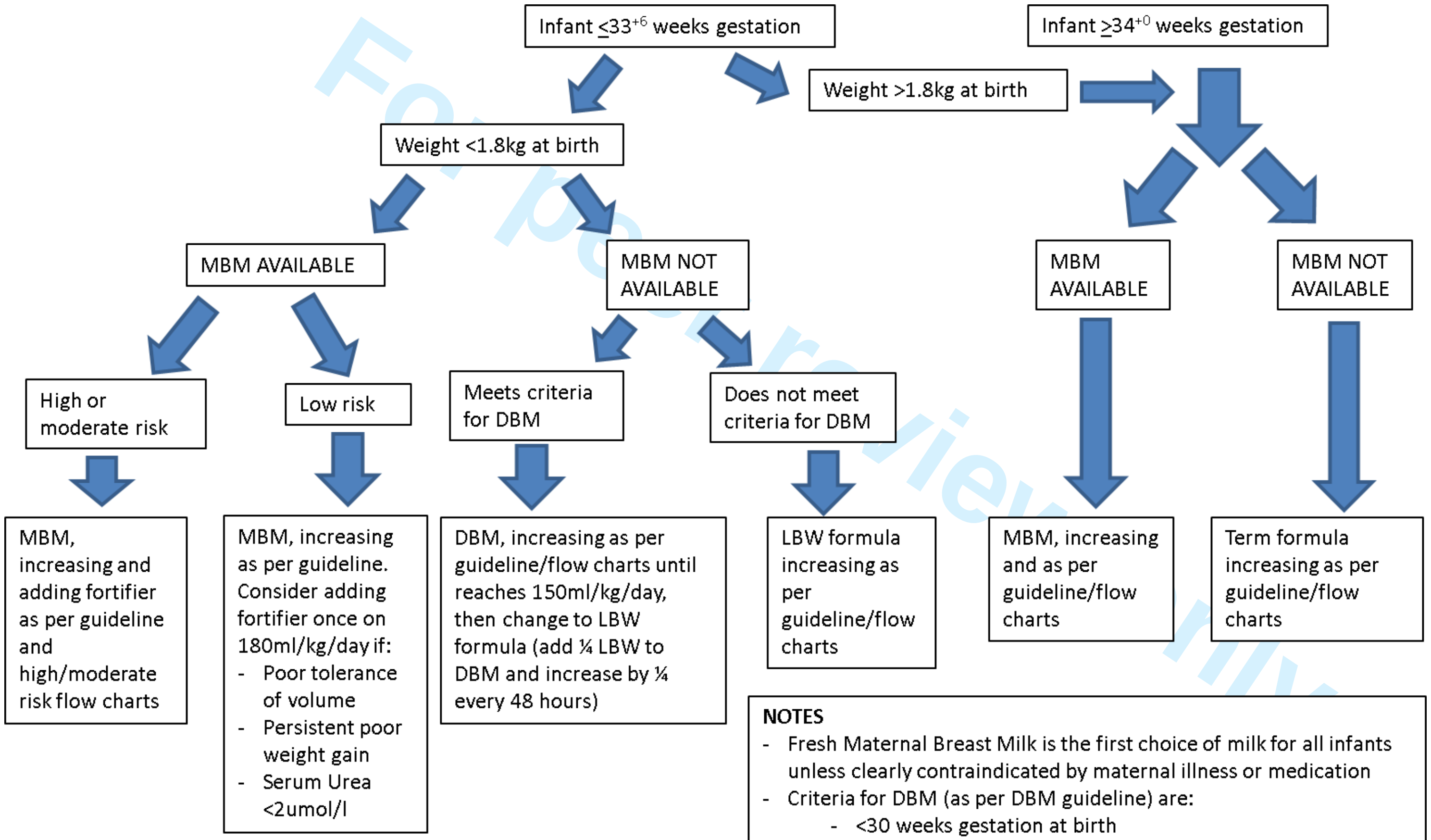

NOTES

- Fresh Maternal Breast Milk is the first choice of milk for all infants unless clearly contraindicated by maternal illness or medication
- Criteria for DBM (as per DBM guideline) are:
  - <30 weeks gestation at birth
  - Weight <1.2kg at birth
  - >30 weeks gestation at birth with IUGR or AREDF
  - Preterm infants with maternal HIV

Disclaimer: It is your responsibility to check against StaffNet that this printout is the most recent issue of this document

For peer review only - http://bmjopen.bmj.com/site/about/guidelines.xhtml

8. TABLES

a. Starting and Increasing Feeds

i. High Risk Infants (based on increases of 10-20ml/kg/day)

Weight (kg)	Start at (hourly)	Start at (2 hourly)	Increase hourly feed volume by*	Increase 2hourly feed volume by
less than 0.6	N/A	0.5	0.25ml every 24 hours	0.5ml every 24 hours
0.6-0.9	0.5	1	0.5ml every 24 hours	1ml every 24 hours
0.9-1.2	0.75	1.5	0.5ml every 12 hours	1ml every 12 hours
1.2-1.5	1	2	0.5ml every 8 hours	1ml every 8 hours
1.5-1.8	1.25	2.5	0.5ml every 6 hours	1ml every 6 hours
1.8-2	1.5	3	1ml every 12 hours	2ml every 12 hours

ii. Moderate Risk Infants (based on increases on 20-30ml/kg/day)

Weight (kg)	Start at (hourly)	Start at (2 hourly)	Increase hourly feed volume by:*	Increase 2hourl feed volume by:
1.0-1.2	1	2	0.5ml every 6 hours	1ml every 6 hours
1.2-1.6	1.5	3	1ml every 12 hours	2ml every 12 hours
1.6-2.0	2	4	1ml every 8 hours	2ml every 8 hours
2-2.4	2.5	5	1ml every 6 hours	2ml every 6 hours
2.4 and above	3	6	1.5ml every 8 hours	3ml every 8 hours

*Note that this refers to the actual feed **volume** based on 1 hourly feeds. Therefore if baby is 2 hourly fed then multiply the amount on this table by 2 to give the increase on the feed volume, if on 3 hourly feeds multiply by 3 and so on.

For peer review only - http://bmjopen.bmj.com/site/about/guidelines.xhtml

University Hospital Southampton NHS
NHS Foundation Trust

Fluid Name / Nutrient	Preterm Stock PN	Preterm + Sodium Stock PN	Term Stock PN	Stock Lipid	Dextrose 10%	MBM/DBM	MBM with Full Fortifier*	Neocate LCP	Peptijunior	LBW Formula (Aptamil Preterm)	Post D/C Formula (Nutriprem 2)	Term formula	Infantrini
**Energy (kcal)**	63.0	59.8	70.2	166.7	40.0	69.0	85.0	71.0	66.0	80.0	75.0	66.0	100.0
**Protein (g)**	2.3	2.8	2.5	0	0.0	1.3	2.5	2.0	1.8	2.6	2.0	1.3	2.6
**Carbohydrate (g)**	12.1	11.0	13.5	0	0.0	7.2	10.0	8.1	6.8	8.4	7.4	7.3	10.3
**Fat (g)**	0	0	0	16.7	0.0	4.1	4.1	3.5	3.5	3.9	4.0	3.5	5.4
**Sodium(mmol)**	0.0	4.3	2.8	0.1	0.0	0.7	2.2	0.8	0.9	3.0	1.2	0.7	1.1
**Potassium (mmol)**	2.4	1.7	1.9	0	0.0	1.5	2.1	1.6	1.7	2.1	2.0	1.6	2.4
**Calcium(mmol)**	0.8	1.0	0.9	0	0.0	0.8	2.5	1.2	1.2	2.3	2.2	1.2	2.0
**Phosphorous (mmol)**	1.0	2.2	0.9	1.5	0.0	0.5	1.7	1.1	0.9	2.0	1.5	0.9	1.3
**Iron (umol)**	0.0	0.0	0.0	0.0	0.0	1.3	1.3	18.8	13.8	25.1	17.9	9.5	21.5
**Vitamin A (IU)**	0.0	0.0	0.0	3910.0	0.0	213.0	985.6	264.0	173.2	599.4	269.7	183.2	333.0
**Vitamin D (IU)**	0.0	0.0	0.0	680.0	0.0	0.0	200.0	51.0	52.0	120.0	68.0	48.0	68.0
**Volume (ml/kg) required to reach recommended protein intake (ELBW infants)**	152	125	140	Contains no protein	Contains no protein	292	152	195	211	146	190	292	146

b. Nutrient Content of Commonly Used Products per 100ml

Typical Values are used and are correct at 18/10/2011

*Based on Cow and Gate Nutriprem Breast Milk Fortifier

For peer review only - http://bmjopen.bmj.com/site/about/guidelines.xhtml

9. SUPPORTING INFORMATION

GUIDELINES AND NUTRITIONAL CARE

There is good evidence from large epidemiological studies such as EPICure that preterm infants often fail to grow adequately, dropping to significantly lower centiles for weight and head circumference at discharge than those which they were born on[9, 10]. There is also evidence that growth failure is also associated with poorer neurodevelopmental outcomes[11]. One significant causative factor for this failure of growth is that these infants receive inadequate nutrition, and there is evidence that they fail to achieve appropriate targets for nutrient intake[12, 13]. Feeding practices across different neonatal units has been shown to be one of the factors responsible for the variability in lengths of stay and the level of postnatal growth restriction seen between different units offering the same level of care[14]. Although there is uncertainty around the definitive practice of nutritional support in preterm infants, there is evidence that standardisation of practice and the use of guidelines is beneficial. A systematic review and meta-analysis by Patole and De Clerk in 2005 showed that the use of standardised feeding regimens reduced rates of NEC, and in the context of the Vermont Oxford Network's 'Potentially Better Practices for Nutrition', the standardisation of practice was shown to reduce the time to start TPN and enteral feeds, improve use of breast milk, reduce lengths of stay and a lower rate of infants being discharges with weights below the 10th centile [4, 15]. Donovan et al studied aspects of nutrient intake and outcomes before and after the introduction of nutrition support guidelines in their NICU, showing significantly earlier initiation of both parenteral and enteral feeding, earlier achievement of full enteral feeding, and earlier regaining of birth-weight after introduction of guidelines[16].

ASSESSMENT AND MONITORING

Some babies are at higher risk than others of nutritional problems – under-nutrition, feed – related complications or both. Regular assessment of nutritional status and monitoring of growth will help identify infants with greater nutritional needs or a higher risk of poor growth or problems. Preterm infants in particular are at risk and should have their weight, head circumference and length measured at a minimum of once a week [4, 6, 17].
The following are things to consider when assessing nutritional risk

- Term babies with appropriate birth weight have good nutrient stores, designed to support them through the first few days when breast milk volumes are low. They are low risk.

Disclaimer: It is your responsibility to check against StaffNet that this printout is the most recent issue of this document

For peer review only - http://bmjopen.bmj.com/site/about/guidelines.xhtml

- Preterm babies have low nutrient stores and are born at time of rapid growth – the earlier they are then the bigger the problem and the greater their nutritional risk. This is compounded by immature gut and metabolic function. They are moderate to high risk (depending on gestation) and need early nutrition support.
- Growth restricted babies have less nutritional reserve; they may also have reduced perfusion to the gut before birth and an increased risk of NEC. These babies will therefore be at greater risk compared to babies of a similar gestation.
- Congenital abnormalities such as gastrointestinal abnormalities, facial anomalies and cardiac problems (including PDA and associated treatment) will all affect nutritional status and increase nutritional risk.
- Acquired disorders such as hypoxic-ischaemic injury, sepsis and NEC will impact on the nutrition infants receive and in turn put them at higher risk of poor nutrition.
- Combinations of the any of the above factors will result in a greater overall risk.

NUTRITIONAL REQUIREMENTS

TERM INFANTS: breast milk provides appropriate nutrients for healthy term babies and breast-feeding should be supported and encouraged. Babies who are not being breast fed should be fed on a standard cows' milk based formula.

PRETERM INFANTS: evidence-based recommendations are available to guide nutrient intakes for preterm infants. The most comprehensive is Tsang 2005 [2], which gives guidelines for parenteral and enteral nutrition support, and specifies requirements for babies <1000g and 1000-1500g birth-weight, during both 'transition' phase (days 2-7 of life) and 'growth phase' (day 7 onwards). ESPHGAN 2010 [1] gives recommendations for enteral intake of fluid and nutrients, though is largely based on the Tsang recommendations. Growth is rapid in the third trimester of fetal life; infants born preterm thus have high requirements for nutrients, but immature physiological capacity to handle them. Breast milk is the optimal first choice for preterm infants' nutrition, however even at high volumes will not provide all adequate nutrients: supplementation with breast milk fortifier or preterm formula may be necessary. The tables in this guideline refer to the Tsang recommendations for energy and protein in VLBW infants and how they compare to typical feeds used in Southampton. Note that only LBW formula milk fed at 150ml/kg/day or fully fortified breast milk fed at 180ml/kg/day is able to achieve the recommended amounts). The full Tsang recommended nutrient intakes are given in Appendix 1. Essentially, the less mature, the lower the nutrient stores/reserves, the earlier nutrient provision is required

STANDARD NUTRITIONAL SUPPORT OF PRETERM AND SICK INFANTS

Disclaimer: It is your responsibility to check against StaffNet that this printout is the most recent issue of this document

For peer review only - http://bmjopen.bmj.com/site/about/guidelines.xhtml

University Hospital Southampton NHS
NHS Foundation Trust

a. PARENTERAL NUTRITION

i. *Early use of PN*

The VON Potentially Better Practices for nutrition state that TPN should be commenced as early as possible, ideally within the first 24 hours of life [4]. This helps prevent the net nutrient loss and catabolism that occurs when an infant is born prematurely. Significant nutritional deficits have been shown to occur in the first few days (up to 2 weeks) after birth, so introduction of TPN early is a strategy to help prevent this [12]. There is also good evidence that it promotes anabolism, prevents the loss of protein mass, improves calorie intakes, can improve growth and is safe [3, 18-21].

ii. *Protein intake*

As described above, nutrient delivery in high risk groups is challenging, and the delivery of protein and energy early in life often fails to meet recommended targets. Whilst intravenous glucose given early on will meet energy needs in many cases, it contains no protein, which can only be administered using TPN or milk feeds. Therefore, in high risk infants who cannot be fully fed quickly, it is vital to give the largest amount of protein possible as TPN, as early as possible to try and prevent the accumulation of deficits. In view of this, Stock TPN in Southampton has recently been reformulated to provide higher levels of protein in a smaller volume. Using high protein TPN to deliver higher protein intakes in the first few days of life in preterm infants has recently been shown to have metabolic benefits in addition to the prevention of catabolism, including a reduction in hyperglycaemia and insulin use [22], and a significant reduction non-oliguric hyperkalaemia [23].

iii. *Peripheral vs central PN*

It is generally accepted that is preferable to given TPN via a percutaneous central venous catheter ('long line') than via a peripheral cannula, in view of the decreased risk of extravasation, the difficulty associated in obtaining repeated peripheral access in preterm infants, and the ability to give higher concentrations of glucose and potassium. Central lines on the other hand have the disadvantage of the risk of catheter related infections. A Cochrane review in 2007 concluded that central TPN was not associated with an increased risk of infection compared to peripheral TPN, and there was some evidence that central TPN resulted in a smaller number of catheters/cannulas per infant required to deliver the TPN, together with improved nutrient delivery [24]. However, it also concluded that there was no significant

Disclaimer: It is your responsibility to check against StaffNet that this printout is the most recent issue of this document

For peer review only - http://bmjopen.bmj.com/site/about/guidelines.xhtml

difference in adverse events (including extravasation) when comparing central to peripheral TPN. Therefore, whilst TPN should be given centrally wherever possible, peripheral TPN should be considered in some individual cases where there is significant nutritional risk and a delay or difficulty in obtaining central access [3].

iv. *Monitoring and Complications*

Careful monitoring of patients whilst on TPN is important to ensure appropriate and adequate nutrition, and to identify potential complications, including liver disease, metabolic bone disease and catheter-related infection. Current recommendations regarding monitoring have been laid out by ESPGHANs guidelines on paediatric parenteral nutrition[3]., and can be found in the NNU Parenteral Nutrition Guidebook

b. ENTERAL FEEDING

i. *Choice of milk*

There is good evidence that maternal breast milk (and to some extent donor breast milk) is protective against NEC, so breast milk should be the food of first choice [25-30]. Ideally this should be the mother's own fresh colostrum. All mothers of preterm infants should have lactation support, and help with expressing within 6 hours of birth (ideally within half an hour according to current WHO recommendations)[5]. If no maternal milk available by 48 hours and the baby is ready for milk, consent should be sought to use DBM. However, as DBM is a limited resource and there is evidence it contains fewer nutrients than mother's own breast milk, DBM should be reserved only for the purposes of establishing feeds in high risk infants, as laid out in the DBM guideline). Where breast milk cannot be used, preterm infants should receive a specialist high calorie and high protein formula ('LBW formula')[31-33].Preterm formulas are designed to meet the basic nutritional requirements of most preterm infants when fed between 150 and 180ml/kg. Preterm formulas can be used as soon as commencement of enteral feeding is recommended. Term formulas should not be used as they fail to meet the nutritional needs of premature infants. There is no evidence to support the use of term elemental/semi elemental formulas in the early stages of feeding unless there is a compelling clinical reason to do so.

ii. *Starting Feeds*

The objective of early feeding is to stimulate gut maturation, motility and hormone release. As starvation leads to atrophy of the gut, withholding feeds

Disclaimer: It is your responsibility to check against StaffNet that this printout is the most recent issue of this document

For peer review only - http://bmjopen.bmj.com/site/about/guidelines.xhtml

may render subsequent feeding less safe and protract the time to reach full enteral feeding [34]. No work has yet addressed whether initial feeds should be exclusively breast milk (mother's own or donor) or whether initial feeds should be delayed if only formula is available. However most evidence suggests that any enteral feed given early may be better than gut starvation [35].

Trophic feeding is defined as small volumes of enteral feeds up to 24 mls/kg/day given to promote gut function It has been shown to prevent changes of starvation in gut mucosa, but a systematic review of 9 trials of trophic feeds vs withholding feed, including 754 infants, did not find any difference in overall feed tolerance, weight gain or rates of NEC [36].

Due to concerns about NEC, commencement of enteral feeds is sometimes delayed in preterm infants. A Cochrane review of early vs delayed introduction of progressive enteral feeds did not show an increase in NEC with early feeds, but despite almost 1000 babies in 5 RCTs the conclusion was that data was insufficient [37]. The ADEPT trial randomised 404 preterm, growth-restricted babies to early feeds (start day 2) or late feeds (start day 6): the early group achieved full feeding earlier, required less PN and had less cholestasis, and no difference was seen in incidence of NEC [38]. There is thus no evidence to support delaying feeds; there is a lack of good evidence to guide feeding policy in babies on inotropes and ibuprofen.

iii. *Rate of advancing feeds*

In standard risk infants a rate of increase of 30ml/kg/day is reported safe, whereas data is more limited in the high risk infant. Evidence points towards several days of trophic feeds followed by a rate of increase of 10-20ml/kg/day. There should be a low threshold for withholding stepped increases secondary to tolerance concerns in the high risk infants. There is limited data on this. A Cochrane review [39] including 4 RCTs and 496 babies, considered increase of up to 24 mls/kg/day as slow, and 25 or greater mls/kg/day as rapid. More rapid increase was associated with earlier tolerance of full feeds and faster weight gain, and no difference in NEC, but numbers were too small to make definite conclusions. This topic is being considered by NIHR for a multi-centre UK trial at present.

iv. *Nutritional Supplements:*

As mentioned above, the nutritional needs of preterm infants are greater than infants born at term, and as such breast milk is adequate to meet those

Disclaimer: It is your responsibility to check against StaffNet that this printout is the most recent issue of this document

For peer review only - http://bmjopen.bmj.com/site/about/guidelines.xhtml

needs [2]. In order to maintain the benefits of breast milk whilst optimising the nutritional status and growth of preterm infants' single multicomponent fortifiers (BMF) have been developed.

Concerns with the use of BMFs include tolerance and their effects to increasing osmolality and in turn the risk of NEC. Most studies have found no significant problems with the tolerance of fortified EBM [40], and a recent review of published evidence found no link between the relatively small increases in osmolality caused by the addition of fortifier to breast milk and NEC [41]. A Cochrane review concluded that the use of BMFs can lead to short term improvements in weight, length and head circumference and that while it is unlikely that further comparative studies with breast milk alone are to take place it recommends further research seeks to evaluate long term outcomes of BMF therapy and identify the optimum composition of BMF products [42].

Recommendations made in 2010 by ESPGHAN stated that the feed of choice for preterm infants (<1800g) was mother's own breast milk supplemented with BMF, or special preterm formula if breast milk not available [1].

v. *Nutrition at Discharge:*

Preterm infants are often discharged home with growth below that expected according to their birth centile. A review by ESPGHAN in 2006 looking at the evidence for feeding preterm infants after discharge recommended that infants discharged with an appropriate weight for their corrected gestational age should be discharged either breast feeding (where breast fed) or on regular formula (where formula fed). However, they also concluded that preterm infants discharged with a subnormal weight for their corrected gestation age should receive fortifier in addition to breast milk (where breast fed) or on special high energy/protein preterm infant formula (where formula fed) [43]. Recently, a Cochrane review looked at this in more detail, addressing the question of whether using fortifier in breast fed preterm infants after discharge improved growth. It concluded that using fortifier after discharge improved growth in infancy, though the evidence was limited [44].

Disclaimer: It is your responsibility to check against StaffNet that this printout is the most recent issue of this document

For peer review only - http://bmjopen.bmj.com/site/about/guidelines.xhtml

6. References

1. Agostoni, C., et al., *Enteral nutrient supply for preterm infants: commentary from the European Society of Paediatric Gastroenterology, Hepatology and Nutrition Committee on Nutrition.* J Pediatr Gastroenterol Nutr, 2010. **50**(1): p. 85-91.
2. Tsang, R.C., *Nutrition of the preterm infant*. 2nd ed. ed. 2005, Cincinnati: Digital Educational Publishing. viii, 427 p.
3. Koletzko, B., et al., *1. Guidelines on Paediatric Parenteral Nutrition of the European Society of Paediatric Gastroenterology, Hepatology and Nutrition (ESPGHAN) and the European Society for Clinical Nutrition and Metabolism (ESPEN), Supported by the European Society of Paediatric Research (ESPR).* J Pediatr Gastroenterol Nutr, 2005. **41 Suppl 2**: p. S1-87.
4. Kuzma-O'Reilly, B., et al., *Evaluation, development, and implementation of potentially better practices in neonatal intensive care nutrition.* Pediatrics, 2003. **111**(4 Pt 2): p. e461-70.
5. WHO, *Management and support of infant feeding in maternity facilities*, in *Infant and young child feeding : model chapter for textbooks for medical students and allied health professionals.* 2009, World Health Organisation. p. 29-36.
6. Edmond, K. and R. Bahl, *Optimal feeding of low-birth-weight infants*. 2006, World Health Organisation.
7. Hartnoll, G., P. Betremieux, and N. Modi, *Randomised controlled trial of postnatal sodium supplementation on body composition in 25 to 30 week gestational age infants.* Arch Dis Child Fetal Neonatal Ed, 2000. **82**(1): p. F24-8.
8. Vandenplas, Y., et al., *Guidelines for the diagnosis and management of cow's milk protein allergy in infants.* Arch Dis Child, 2007. **92**(10): p. 902-8.
9. Wood, N.S., et al., *The EPICure study: growth and associated problems in children born at 25 weeks of gestational age or less.* Archives of Disease in Childhood Fetal & Neonatal Edition, 2003. **88**(6): p. F492-500.
10. Ehrenkranz, R.A., et al., *Longitudinal growth of hospitalized very low birth weight infants.* Pediatrics, 1999. **104**(2 Pt 1): p. 280-9.
11. Ehrenkranz, R.A., et al., *Growth in the neonatal intensive care unit influences neurodevelopmental and growth outcomes of extremely low birth weight infants.* Pediatrics, 2006. **117**(4): p. 1253-61.
12. Embleton, N.E., N. Pang, and R.J. Cooke, *Postnatal malnutrition and growth retardation: an inevitable consequence of current recommendations in preterm infants?* Pediatrics, 2001. **107**(2): p. 270-3.
13. Martin, C.R., et al., *Nutritional practices and growth velocity in the first month of life in extremely premature infants.* Pediatrics, 2009. **124**(2): p. 649-57.
14. Cooke, R.J., S.B. Ainsworth, and A.C. Fenton, *Postnatal growth retardation: a universal problem in preterm infants.* Arch Dis Child Fetal Neonatal Ed, 2004. **89**(5): p. F428-30.
15. Patole, S.K. and N. de Klerk, *Impact of standardised feeding regimens on incidence of neonatal necrotising enterocolitis: a systematic review and meta-analysis of observational studies.* Arch Dis Child Fetal Neonatal Ed, 2005. **90**(2): p. F147-51.
16. Donovan, R., et al., *Outcomes of early nutrition support in extremely low-birth-weight infants.* Nutr Clin Pract, 2006. **21**(4): p. 395-400.
17. *Malnutrition- What nurses working with children and young people need to know and do. An RCN position statement*. 2006, Royal College of Nursing.
18. Anderson, T.L., et al., *A controlled trial of glucose versus glucose and amino acids in premature infants.* J Pediatr, 1979. **94**(6): p. 947-51.
19. Ibrahim, H.M., et al., *Aggressive early total parental nutrition in low-birth-weight infants.* J Perinatol, 2004. **24**(8): p. 482-6.
20. Wilson, D.C., et al., *Randomised controlled trial of an aggressive nutritional regimen in sick very low birthweight infants.* Arch Dis Child Fetal Neonatal Ed, 1997. **77**(1): p. F4-11.

Disclaimer: It is your responsibility to check against StaffNet that this printout is the most recent issue of this document

For peer review only - http://bmjopen.bmj.com/site/about/guidelines.xhtml

21. Van Goudoever, J.B., et al., *Immediate commencement of amino acid supplementation in preterm infants: effect on serum amino acid concentrations and protein kinetics on the first day of life.* J Pediatr, 1995. **127**(3): p. 458-65.
22. Mahaveer, A., C. Grime, and C. Morgan, *Increasing early protein intake is associated with a reduction in insulin-treated hyperglycaemia in very preterm infants.* Arch Dis Child Fetal Neonatal Ed, 2011. **96**(Suppl 1): p. Fa21.
23. Iacobelli, S., et al., *Standardized parenteral nutrition in preterm infants: early impact on fluid and electrolyte balance.* Neonatology, 2010. **98**(1): p. 84-90.
24. Ainsworth, S.B., L. Clerihew, and W. McGuire, *Percutaneous central venous catheters versus peripheral cannulae for delivery of parenteral nutrition in neonates.* Cochrane Database Syst Rev, 2007(3): p. CD004219.
25. Lucas, A. and T.J. Cole, *Breast milk and neonatal necrotising enterocolitis.* Lancet, 1990. **336**(8730): p. 1519-23.
26. McGuire, W. and M.Y. Anthony, *Donor human milk versus formula for preventing necrotising enterocolitis in preterm infants: systematic review.* Arch Dis Child Fetal Neonatal Ed, 2003. **88**(1): p. F11-4.
27. Schanler, R.J., et al., *Randomized trial of donor human milk versus preterm formula as substitutes for mothers' own milk in the feeding of extremely premature infants.* Pediatrics, 2005. **116**(2): p. 400-6.
28. Boyd, C.A., M.A. Quigley, and P. Brocklehurst, *Donor breast milk versus infant formula for preterm infants: systematic review and meta-analysis.* Arch Dis Child Fetal Neonatal Ed, 2007. **92**(3): p. F169-75.
29. Schanler, R.J., *Suitability of human milk for the low-birthweight infant.* Clin Perinatol, 1995. **22**(1): p. 207-22.
30. Henderson, G., M.Y. Anthony, and W. McGuire, *Formula milk versus maternal breast milk for feeding preterm or low birth weight infants.* Cochrane Database Syst Rev, 2007(4): p. CD002972.
31. Premji, S.S., T.R. Fenton, and R.S. Sauve, *Higher versus lower protein intake in formula-fed low birth weight infants.* Cochrane Database Syst Rev, 2006(1): p. CD003959.
32. Atkinson, S.A., et al., *Randomized Trial of Feeding Nutrient-Enriched vs Standard Formula to Premature Infants during the First Year of Life.* Pediatric Research. **45**(4): p. 276A.
33. Lucas, A., F. King, and N.B. Bishop, *Postdischarge formula consumption in infants born preterm.* Arch Dis Child, 1992. **67**(6): p. 691-2.
34. Ziegler, E.E., P.J. Thureen, and S.J. Carlson, *Aggressive nutrition of the very low birthweight infant.* Clin Perinatol, 2002. **29**(2): p. 225-44.
35. King, C., *What's new in enterally feeding the preterm infant?* Arch Dis Child Fetal Neonatal Ed, 2010. **95**(4): p. F304-8.
36. Bombell, S. and W. McGuire, *Early trophic feeding for very low birth weight infants.* Cochrane Database Syst Rev, 2009(3): p. CD000504.
37. Morgan, J., L. Young, and W. McGuire, *Delayed introduction of progressive enteral feeds to prevent necrotising enterocolitis in very low birth weight infants.* Cochrane Database Syst Rev, 2011(3): p. CD001970.
38. Leaf, A., et al., *Early or late enteral feeding for preterm growth-restricted infants? The abnormal Doppler enteral prescription trial.* Archives of Disease in Childhood, 2010. **95**: p. A3.
39. Morgan, J., L. Young, and W. McGuire, *Slow advancement of enteral feed volumes to prevent necrotising enterocolitis in very low birth weight infants.* Cochrane Database Syst Rev, 2011(3): p. CD001241.
40. Lucas, A., et al., *Randomized outcome trial of human milk fortification and developmental outcome in preterm infants.* Am J Clin Nutr, 1996. **64**(2): p. 142-51.
41. Pearson, F., M.J. Johnson, and A.A. Leaf, *Milk Osmolality - Does it Matter?* Arch Dis Child Fetal Neonatal Ed, 2011. **In press**.
42. Kuschel, C.A. and J.E. Harding, *Multicomponent fortified human milk for promoting growth in preterm infants.* Cochrane Database Syst Rev, 2004(1): p. CD000343.

Disclaimer: It is your responsibility to check against StaffNet that this printout is the most recent issue of this document

For peer review only - http://bmjopen.bmj.com/site/about/guidelines.xhtml

43. Aggett, P.J., et al., *Feeding preterm infants after hospital discharge: a commentary by the ESPGHAN Committee on Nutrition.* J Pediatr Gastroenterol Nutr, 2006. **42**(5): p. 596-603.
44. McCormick, F.M., et al., *Multinutrient fortification of human breast milk for preterm infants following hospital discharge.* Cochrane Database Syst Rev, 2010(7): p. CD004866.

For peer review only

For peer review only - http://bmjopen.bmj.com/site/about/guidelines.xhtml

University Hospital Southampton NHS
NHS Foundation Trust

Appendix 1- Nutritional requirements of Preterm Infants

	Extremely Low Birth Weight (<1000g)											
	parenteral						enteral					
	Day 0 Lower	Day 0 Upper	Trans Lower	Trans Upper	Grow Lower	Grow Upper	Day 0 Lower	Day 0 Upper	Trans Lower	Trans Upper	Grow Lower	Grow Upper
**Energy (kcal)**	40	50	75	85	105	115	50	60	90	100	130	150
**Protein (g)**	2	2	3.5	3.5	3.5	4	2	2	3.5	3.5	3.8	4.4
**Carbohydrate (g)**	7	7	8	15	13	17	7	7	8	15	9	20
**Fat (g)**	1	1	1	3	3	4	1	1	1	3	3.2	8.4
**Sodium (mmol)**	0	1	2	5	3	5	0	1	2	5	3	5
**Chloride (mmol)**	0	1	2	5	3	7	0	1	2	5	3	7
**Potassium (mmol)**	0	0	0	2	2	3	0	0	0	2	2	3
**Calcium (mmol)**	0.5	1.5	1.5	1.5	1.5	2	0.8	2.5	2.5	2.5	2.5	5.5
**Phosphorous (mmol)**	0	0	1.5	1.9	1.5	1.9	0.6	1.9	1.9	4.5	1.9	4.5
**Magnesium (mmol)**	0	0	0.2	0.3	0.2	0.3	0.1	0.3	0.3	0.6	0.3	0.6
**Iron (umol)**	0	0	0	0	1.8	3.6	0	0	0	0	35.8	71.6
**Zinc (umol)**	0	2.3	2.3	2.3	6.1	6.1	0	15.3	6.1	18.3	15.3	45.9
**Copper (umol)**	0	0	0	0.3	0.3	0.3	0	0	0	2.4	1.9	2.4
**Selenium (nmol)**	0	0	0	16.5	19	57	0	0	0	16.5	16.5	57
**Iodine (nmol)**	0	0	0	8	7.9	7.9	0	0	0	473	79	473
**Manganese (nmol)**	0	0	0	13.7	18.2	18.2	0	0	0	137	13	137
**Vitamin A (IU)**	700	1500	700	1500	700	1500	700	1500	700	1500	700	1500
**Vitamin D (IU)**	40	160	40	160	40	160	150	400	150	400	150	400
**Vitamin E (IU)**	2.8	3.5	2.8	3.5	2.8	3.5	6	12	6	12	6	12
**Vitamin K (ug)**	0	0	22	22	22	22	0	0	18	22	18	22
**Thiamin (ug)**	200	350	200	350	300	350	180	240	180	240	180	240
**Riboflavin (ug)**	150	200	150	200	150	200	250	360	250	360	250	360
**Vitamin B6 (ug)**	150	200	150	200	150	200	150	210	150	210	150	210
**Folate (ug)**	56	56	56	56	56	56	25	50	25	50	25	50
**Vitamin B12 (ug)**	0.3	0.3	0.3	0.3	0.3	0.3	0.3	0.3	0.3	0.3	0.3	0.3
**Biotin (ug)**	5.8	5.8	5.8	5.8	5.8	5.8	3.6	6	3.6	6	3.6	6
**Pantothenic Acid (mg)**	1.2	1.2	1.2	1.2	1.2	1.2	1.2	1.7	1.2	1.7	1.2	1.7
**Niacin (mg)**	4	6.8	4	6.8	4	4.6	3.6	4.8	3.6	4.8	3.6	4.8
**Vitamin C (mg)**	15	25	15	25	15	25	18	24	18	24	18	24
**Taurine (mg)**	0	3.75	1.88	3.75	1.88	3.75	0	9	4.5	9	4.5	9
**Choline (mg)**	0	28	14.4	28	14.4	28	0	28	14.4	28	14.4	28
**Carnitine (mg)**	0	2.9	2.9	2.9	2.9	2.9	0	2.9	2.9	2.9	2.9	2.9
**Inositol (mg)**	0	54	54	54	54	54	0	54	32	81	32	81

	Very Low Birth Weight (<1500g)											
	parenteral						enteral					
	Day 0 Lower	Day 0 Upper	Trans Lower	Trans Upper	Grow Lower	Grow Upper	Day 0 Lower	Day 0 Upper	Trans Lower	Trans Upper	Grow Lower	Grow Upper
**Energy (kcal)**	40	50	60	70	90	100	50	60	75	90	110	130
**Protein (g)**	2	2	3.5	3.5	3.2	3.8	2	2	3.5	3.5	3.4	4.2
**Carbohydrate (g)**	7	7	5	12	9.7	15	7	7	5	12	7	17
**Fat (g)**	1	1	1	3	3	4	1	1	1	3	5.3	7.2
**Sodium (mmol)**	0	1	2	5	3	5	0	1	2	5	3	5
**Chloride (mmol)**	0	1	2	5	3	7	0	1	2	5	3	7
**Potassium (mmol)**	0	0	0	2	2	3	0	0	0	2	2	3
**Calcium (mmol)**	0.5	1.5	1.5	1.5	1.5	2	0.8	2.5	2.5	2.5	2.5	5.5
**Phosphorous (mmol)**	0	0	1.5	1.9	1.5	1.9	0.6	1.9	1.9	4.5	1.9	4.5
**Magnesium (mmol)**	0	0	0.2	0.3	0.2	0.3	0.1	0.3	0.3	0.6	0.3	0.6
**Iron (umol)**	0	0	0	0	1.8	3.6	0	0	0	0	35.8	71.6
**Zinc (umol)**	0	2.3	2.3	2.3	6.1	6.1	0	15.3	6.1	18.3	15.3	45.9
**Copper (umol)**	0	0	0	0.3	0.3	0.3	0	0	0	2.4	1.9	2.4
**Selenium (nmol)**	0	0	0	16.5	19	57	0	0	0	16.5	16.5	57
**Iodine (nmol)**	0	0	0	8	7.9	7.9	0	0	0	473	79	473
**Manganese (nmol)**	0	0	0	13.7	18.2	18.2	0	0	0	137	13	137
**Vitamin A (IU)**	700	1500	700	1500	700	1500	700	1500	700	1500	700	1500
**Vitamin D (IU)**	40	160	40	160	40	160	150	400	150	400	150	400
**Vitamin E (IU)**	2.8	3.5	2.8	3.5	2.8	3.5	6	12	6	12	6	12
**Vitamin K (ug)**	0	0	22	22	22	22	0	0	18	22	18	22
**Thiamin (ug)**	200	350	200	350	300	350	180	240	180	240	180	240
**Riboflavin (ug)**	150	200	150	200	150	200	250	360	250	360	250	360
**Vitamin B6 (ug)**	150	200	150	200	150	200	150	210	150	210	150	210
**Folate (ug)**	56	56	56	56	56	56	25	50	25	50	25	50
**Vitamin B12 (ug)**	0.3	0.3	0.3	0.3	0.3	0.3	0.3	0.3	0.3	0.3	0.3	0.3
**Biotin (ug)**	5.8	5.8	5.8	5.8	5.8	5.8	3.6	6	3.6	6	3.6	6
**Pantothenic Acid (mg)**	1.2	1.2	1.2	1.2	1.2	1.2	1.2	1.7	1.2	1.7	1.2	1.7
**Niacin (mg)**	4	6.8	4	6.8	4	4.6	3.6	4.8	3.6	4.8	3.6	4.8
**Vitamin C (mg)**	15	25	15	25	15	25	18	24	18	24	18	24
**Taurine (mg)**	0	3.75	1.88	3.75	1.88	3.75	0	9	4.5	9	4.5	9
**Choline (mg)**	0	28	14.4	28	14.4	28	0	28	14.4	28	14.4	28
**Carnitine (mg)**	0	2.9	2.9	2.9	2.9	2.9	0	2.9	2.9	2.9	2.9	2.9
**Inositol (mg)**	0	54	54	54	54	54	0	54	32	81	32	81

For peer review only

For peer review only - http://bmjopen.bmj.com/site/about/guidelines.xhtml

Clinical Guidance

University Hospital Southampton NHS
NHS Foundation Trust

Affix Patient Label Here

Neonatal Nutritional Screening Tool

To be completed on admission and weekly (every Monday)

Gestation at birth: **Birth Weight:**

1. Assess Growth

Current Weight:		Current Centile:		Birth Centile:	
Current OFC:		Current Centile:		Birth Centile:	
Current Length:		Current Centile:		Birth Centile:	

2. Determine Risk Category

		Tick
HIGH RISK	*Any one of:*<br>• Preterm <28 weeks at birth<br>• Extremely Low Birth Weight < 1000g<br>• Severe IUGR (weight < 2nd centile and AREDFV) <35 weeks<br>• Infant establishing feeds after episode of NEC or GI perforation<br>• Infants with severe congenital GI malformation: gastroschisis<br>• Perinatal hypoxia / ischaemia with multi-organ dysfunction	
MODERATE RISK	*Any one of:*<br>• Preterm 28-31^{+6} weeks, otherwise well<br>• Very Low Birth Weight 1000 - 1500g<br>• Moderate IUGR (weight < 9th centile and AREDFV) <35 weeks<br>• Baby on inotropes<br>• Baby on indomethacin/ibuprofen<br>• Illness or congenital anomaly which may compromise feeding<br>• Polycythaemia	
LOW RISK	*Any one of:*<br>• Preterm 32-36^{+6} weeks, otherwise well<br>• AREDFV / IUGR ≥35 weeks	
NO RISK	• Well Term Infant ≥37 weeks	

3. Determine the need for nutrition team review

The nutriton team should review any infant meeting the following criteria:

	Tick
• High Risk Infants according to criteria above	
• Not regained birth weight by 2 weeks of age	
• >15% weight loss at any time	
• Weight gain <10g/kg/day from 2 weeks of age onwards	
• Drop through 2 centile lines for weight/HC/length	
• Intake <150ml/kg/day from 2 weeks of age onwards	
• NEC or GI surgery at any time	

Name of person completing assessement: ____________ Signature: ____________

If completing a first assessment on admission, please place this form in the plastic wallet in the baby's clear plastic nursing folder, next to the nutrition flow charts

If completing a a weekly asessment, please place this form in the box outside Room 3 once filled out

Disclaimer: It is your responsibility to check against StaffNet that this printout is the most recent issue of this document

For peer review only - http://bmjopen.bmj.com/site/about/guidelines.xhtml

Clinical Guidance

University Hospital Southampton NHS
NHS Foundation Trust

Nutrition Team Review

Date: ______________ Staff Present:

Day: ______________

Gestation at Birth: ______________

Corrected Gestation: ______________

Current Clinical Issues:

Fluid Intake

Total Prescribed Fluids: ml/kg/day

Enteral Feed Type: Parenteral Feed Type:

Nutrient Intake

Enteral Feed Provides:

Milk Feeds:	ml/kg/day	kcal/kg/day	g/kg/day Protein

Parenteral Feed Provides:

Aqueous PN:	ml/kg/day	kcal/kg/day	g/kg/day Protein
Lipid:	ml/kg/day	kcal/kg/day	
**Total Intake:**	ml/kg/day	kcal/kg/day	g/kg/day Protein
			g/kg/day Lipid

Comments on intake:

Bloods

Hb:	Sodium:	Creatinine:	ALP:
CRP:	Potassium:	Albumin:	ALT:
Other:	Urea:	Bili:	Magnesium:
	Calcium (corr):		Phosphate:

Assessment

Recommendations

Disclaimer: It is your responsibility to check against StaffNet that this printout is the most recent issue of this document

For peer review only - http://bmjopen.bmj.com/site/about/guidelines.xhtml

Additional File 2: Data tables to accompany Figure 3 (mean daily nutrient intakes across stay) and Figure 4 (growth over stay)

		Mean Daily Energy Intake in kcal/kg/day (95% CI)		Mean Daily Protein Intake in g/kg/day (95% CI)		Mean Daily Energy Intake as a percentage of RRI (95% CI)		Mean Daily Protein Intake as a percentage of RRI (95% CI)	
**Period**	**Degrees of Freedom**	Unadjusted	Adjusted for sex, gestational age and weight at birth	Unadjusted	Adjusted for sex, gestational age and weight at birth	Unadjusted	Adjusted for sex, gestational age and weight at birth	Unadjusted	Adjusted for sex, gestational age and weight at birth
A. Pre-implementation period (January 1st - July 31st 2011)	10190	115.17 (111.79 to 118.54)	114.51 (111.07 to 117.96)	2.88 (2.77 to 2.98)	2.87 (2.76 to 2.98)	105.31 (103.00 to 107.61)	102.42 (100.45 to 104.39)	79.56 (77.35 to 81.77)	79.19 (76.92 to 81.45)
B. Partial implementation period (August 1st – December 31st 2011)	10190	115.77 (112.61 to 118.94)	115.21 (112.00 to 118.42)	3.09 (2.99 to 3.19)	3.09 (2.98 to 3.19)	102.69 (100.50 to 104.88)	100.86 (98.93 to 102.79)	83.53 (81.42 to 85.65)	83.25 (81.10 to 85.40)
C. Main Intervention Period (January 1st - December 31st 2012)	10190	117.87 (115.23 to 120.52)	117.49 (114.82 to 120.16)	3.20 (3.12 to 3.28)	3.20 (3.12 to 3.28)	100.75 (98.95 to 102.54)	100.58 (99.07 to 102.09)	85.70 (83.97 to 87.42)	85.53 (83.78 to 87.28)
D. Post-implementation period (January 1st - June 30th 2013)	10190	120.45 (116.83 to 124.07)	120.25 (116.61 to 123.89)	3.34 (3.23 to 3.46)	3.34 (3.23 to 3.46)	96.50 (94.03 to 98.98)	97.27 (95.18 to 99.36)	86.79 (84.42 to 89.17)	86.82 (84.42 to 89.22)

Detailed Results of the generalized linear model with mixed effects for nutrient intakes across all 4 study periods. (RRI- reasonable range of intake, CI-confidence interval)

	Mean Difference in Daily Energy Intake kcal/kg/day				Mean Difference in Daily Protein Intake g/kg/day				Mean Difference in Daily Energy Intake as a percentage of RRI				Mean Difference in Daily Protein Intake as a percentage of RRI			
**Comparison**	Unadjusted	p value	Adjusted for sex, gestational age and weight at	p value	Unadjusted	p value	Adjusted for sex, gestational age and weight at	p value	Unadjusted	p value	Adjusted for sex, gestational age and weight at	p value	Unadjusted	p value	Adjusted for sex, gestational age and weight at	p value
A vs B	-0.601	0.986	-0.698	0.979	-0.216	0.001	-0.215	0.001	2.612	0.162	1.559	0.536	-3.971	0.006	-4.066	0.005
A vs C	-2.704	0.549	-2.974	0.47	-0.33	<0.001	-0.33	<0.001	4.559	0.007	1.843	0.431	-6.136	<0.001	-6.345	<0.001
A vs D	-5.279	0.143	-5.733	0.101	-0.472	<0.001	-0.473	<0.001	8.802	<0.001	5.149	0.002	-7.232	<0.001	-7.633	<0.001
B vs C	-2.103	0.638	-2.276	0.577	-0.114	0.169	-0.115	0.169	1.947	0.409	0.283	0.994	-2.165	0.283	-2.28	0.242
B vs D	-4.678	0.19	-5.035	0.144	-0.256	0.003	-0.257	0.003	6.19	0.001	3.59	0.058	-3.262	0.163	-3.568	0.113
C vs D	-2.575	0.543	-2.759	0.489	-0.142	0.087	-0.143	0.091	4.243	0.01	3.306	0.031	-1.096	0.837	-1.288	0.766

Pairwise comparison of all study periods using the generalized linear model with mixed effects approach, showing difference between periods. P values <0.05 are highlighted in bold. Unadjusted differences are given together with differences adjusted for sex, gestational age and weight at birth. Tukey's method was used to adjust for multiple comparisons. (RRI- reasonable range of intake)

For peer review only

	Mean Change in Weight SDS from birth (95% Confidence Interval)			Mean Change in Head Circumference from birth (95% Confidence Interval)		
Period	Degrees of Freedom	Unadjusted	Adjusted for sex, gestational age and weight at birth	Degrees of Freedom	Unadjusted	Adjusted for sex, gestational age and weight at birth
A. Pre-implementation period (January 1st - July 31st 2011)	3628	-0.941 (-1.040 to -0.842)	-0.939 (-1.032 to -0.847)	745	-0.989 (-1.290 to -0.687)	-1.0574 (-1.322 to -0.793)
B. Partial implementation period (August 1st – December 31st 2011)	3628	-0.677 (-0.767 to -0.587)	-0.693 (-0.778 to -0.609)	745	-0.819 (-1.089 to -0.548)	-0.908 (-1.153 to -0.662)
C. Main Intervention Period (January 1st - December 31st 2012)	3628	-0.476 (-0.556 to -0.397)	-0.510 (-0.583 to -0.437)	745	-0.685 (-0.855 to -0.515)	-0.738 (-0.884 to -0.591)
D. Post-implementation period (January 1st - June 30th 2013)	3628	-0.342 (-0.445 to -0.239)	-0.3911 (-0.4865 to -0.2957)	745	-0.571 (-0.807 to -0.335)	-0.645 (-0.851 to -0.434)

Detailed Results of the general linear model with mixed effects for the change in standard deviation scores (SDS) during stay across all 4 study periods.

	Mean Change in Weight SDS from birth				Mean Change in Head Circumference SDS from birth			
Comparison	Unadjusted	p value	Adjusted for sex, gestational age and weight at birth	p value	Unadjusted	p value	Adjusted for sex, gestational age and weight at birth	p value
A vs B	-0.264	<0.001	-0.245	<0.001	-0.17	0.823	-0.15	0.83
A vs C	-0.465	<0.001	-0.429	<0.001	-0.304	0.305	-0.32	0.155
A vs D	-0.599	<0.001	-0.548	<0.001	-0.418	0.14	-0.413	0.077
B vs C	-0.201	<0.001	-0.184	<0.001	-0.134	0.796	-0.17	0.582
B vs D	-0.335	<0.001	-0.302	<0.001	-0.248	0.508	-0.263	0.363
C vs D	-0.134	0.028	-0.119	0.055	-0.114	0.827	-0.093	0.867

Pairwise comparison of all study periods using the general linear model with mixed effects approach, showing difference between periods. P values <0.05 are highlighted in bold. Unadjusted differences are given together with differences adjusted for sex, gestational age and weight at birth. Tukey's method was used to adjust for multiple comparisons. (SDS-standard deviation score)

For peer review only

STROBE 2007 (v4) Statement—Checklist of items that should be included in reports of *cohort studies*

Section/Topic	Item #	Recommendation	Reported on page #
**Title and abstract**	1	(*a*) Indicate the study's design with a commonly used term in the title or the abstract	1
		(*b*) Provide in the abstract an informative and balanced summary of what was done and what was found	2
**Introduction**			
Background/rationale	2	Explain the scientific background and rationale for the investigation being reported	4
Objectives	3	State specific objectives, including any prespecified hypotheses	5-6
**Methods**			
Study design	4	Present key elements of study design early in the paper	6-7
Setting	5	Describe the setting, locations, and relevant dates, including periods of recruitment, exposure, follow-up, and data collection	6-7
Participants	6	(*a*) Give the eligibility criteria, and the sources and methods of selection of participants. Describe methods of follow-up	6-8
		(*b*) For matched studies, give matching criteria and number of exposed and unexposed	
Variables	7	Clearly define all outcomes, exposures, predictors, potential confounders, and effect modifiers. Give diagnostic criteria, if applicable	8-9
Data sources/ measurement	8*	For each variable of interest, give sources of data and details of methods of assessment (measurement). Describe comparability of assessment methods if there is more than one group	8-9
Bias	9	Describe any efforts to address potential sources of bias	9-10
Study size	10	Explain how the study size was arrived at	7-8
Quantitative variables	11	Explain how quantitative variables were handled in the analyses. If applicable, describe which groupings were chosen and why	9-10
Statistical methods	12	(*a*) Describe all statistical methods, including those used to control for confounding	9-10
		(*b*) Describe any methods used to examine subgroups and interactions	9-10
		(*c*) Explain how missing data were addressed	10
		(*d*) If applicable, explain how loss to follow-up was addressed	N/A
		(*e*) Describe any sensitivity analyses	9-10
**Results**			

For peer review only

Participants	13*	(a) Report numbers of individuals at each stage of study—eg numbers potentially eligible, examined for eligibility, confirmed eligible, included in the study, completing follow-up, and analysed	11
		(b) Give reasons for non-participation at each stage	N/A
		(c) Consider use of a flow diagram	Figure 2
Descriptive data	14*	(a) Give characteristics of study participants (eg demographic, clinical, social) and information on exposures and potential confounders	11
		(b) Indicate number of participants with missing data for each variable of interest	11, additional file 2
		(c) Summarise follow-up time (eg, average and total amount)	10-14
Outcome data	15*	Report numbers of outcome events or summary measures over time	10-14, figure 3 and 4
Main results	16	(*a*) Give unadjusted estimates and, if applicable, confounder-adjusted estimates and their precision (eg, 95% confidence interval). Make clear which confounders were adjusted for and why they were included	11-13, figure 3and 4, additional file 2
		(*b*) Report category boundaries when continuous variables were categorized	11-13, figure 3and 4, additional file 2
		(*c*) If relevant, consider translating estimates of relative risk into absolute risk for a meaningful time period	N/A
Other analyses	17	Report other analyses done—eg analyses of subgroups and interactions, and sensitivity analyses	N/A
**Discussion**			
Key results	18	Summarise key results with reference to study objectives	15-16
**Limitations**			
Interpretation	20	Give a cautious overall interpretation of results considering objectives, limitations, multiplicity of analyses, results from similar studies, and other relevant evidence	15-19
Generalisability	21	Discuss the generalisability (external validity) of the study results	15-19
**Other information**			
Funding	22	Give the source of funding and the role of the funders for the present study and, if applicable, for the original study on which the present article is based	22

*Give information separately for cases and controls in case-control studies and, if applicable, for exposed and unexposed groups in cohort and cross-sectional studies.

Note: An Explanation and Elaboration article discusses each checklist item and gives methodological background and published examples of transparent reporting. The STROBE checklist is best used in conjunction with this article (freely available on the Web sites of PLoS Medicine at http://www.plosmedicine.org/, Annals of Internal Medicine at http://www.annals.org/, and Epidemiology at http://www.epidem.com/). Information on the STROBE Initiative is available at www.strobe-statement.org.

For peer review only
